# Supplementary material for: The selenocysteine-containing protein SELENOT maintains dopamine signaling in the midbrain to protect mice from hyperactivity disorder
Source: EMBO J. 2025 Apr 7;44(10):2906–27. doi: 10.1038/s44318-025-00430-3 (PMC12084338; doi:10.1038/s44318-025-00430-3)
Supplement: Supplementary file 1 — Appendix [file 44318_2025_430_MOESM1_ESM.pdf]

Appendix for:

The selenocysteine-containing protein SELENOT maintains dopamine signaling in the midbrain to protect mice from hyperactivity disorder

Table of Contents

Appendix Figure S1	Page 2
Appendix Figure S2	Pages 3-4
Appendix Figure S3	Pages 5-6
Appendix Figure S4	Pages 7-8
Appendix Figure S5	Page 9
Appendix Figure S6	Page 10
Appendix Figure S7	Page 11
Appendix Figure S8	Page 12
Appendix Figure S9	Page 13
Appendix Table S1	Page 14

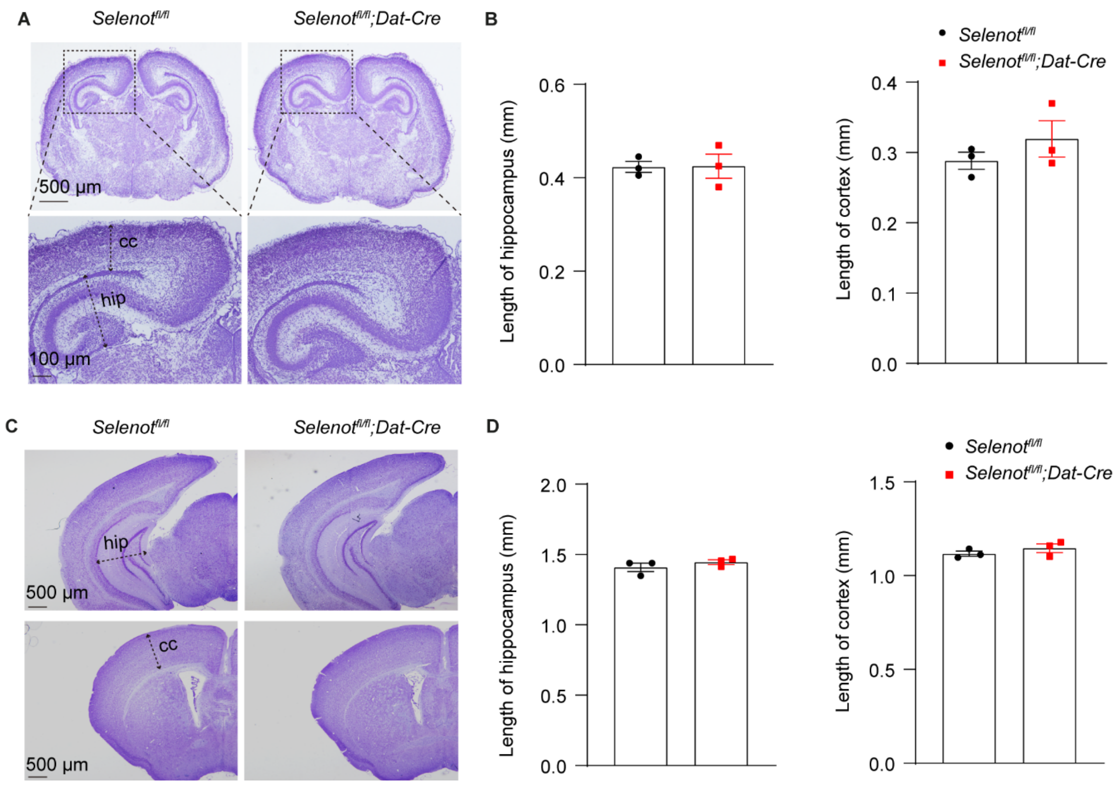

Appendix Figure S1. Brain Nissl staining. (**A** and **B**) Representative coronal sections (**A**) and volume quantifications (**B**) of the cortex (cc) and hippocampus (hip) in mice aged postnatal day 3. (**C** and **D**) Representative coronal sections (**C**) and volume quantifications (**D**) of the cortex and hippocampus in mice at 6 weeks of age. Volume is expressed as the space length indicated by the arrow. Quantification was averaged from 2-4 consecutive slices for each mouse. n = 3 *Selenot*$^{fl/fl}$ mice and n = 3 *Selenot*$^{fl/fl}$*;Dat-cre* mice. Data are presented as means ± SEM and analyzed by two-tailed unpaired *t*-test.

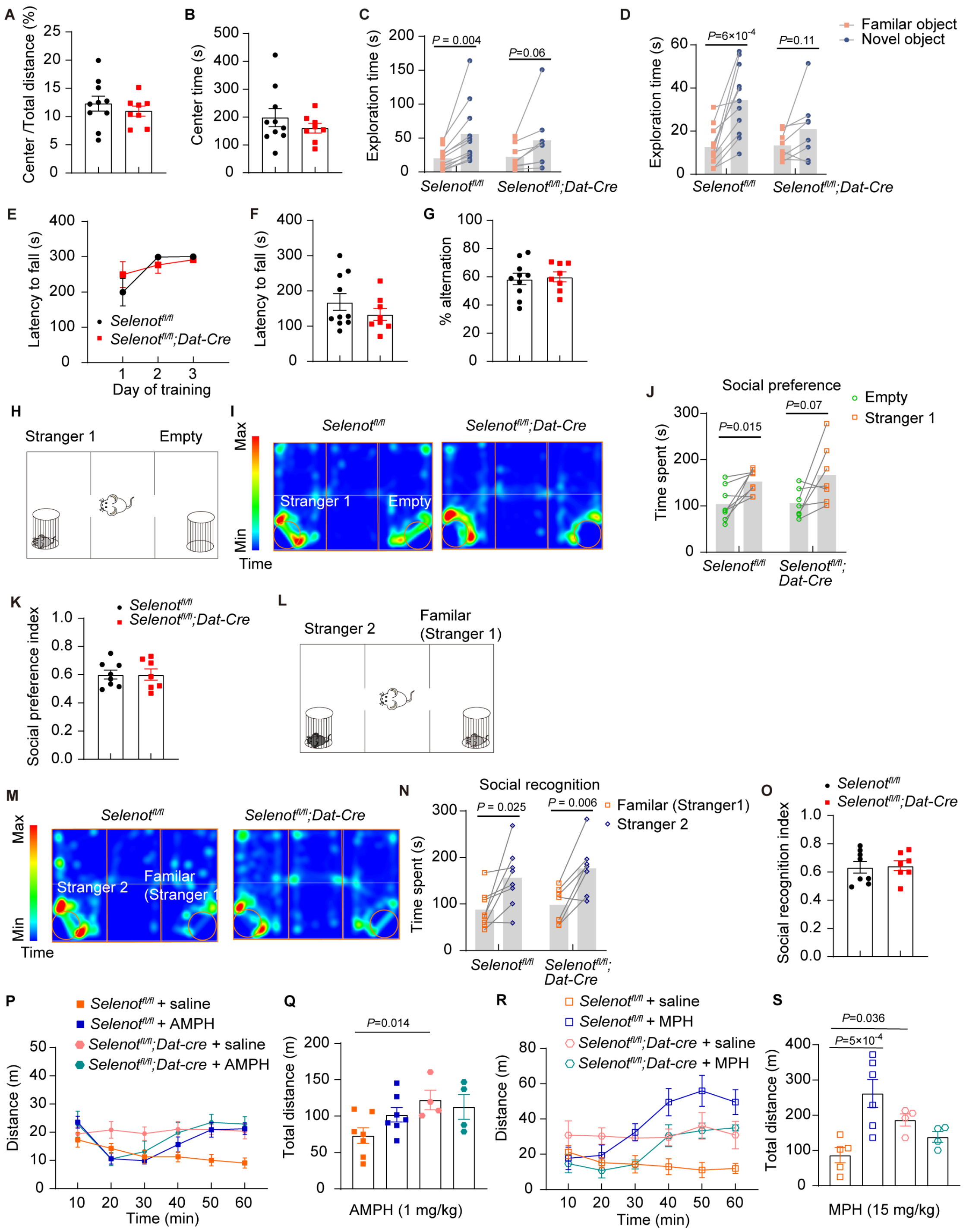

Appendix Figure S2. Additional behavioral assessments for *Selenot$^{fl/fl}$;Dat-cre* mice. (**A** and **B**) Percentage of center distance to total distance (**A**) and center time (**B**) in the open field test of *Selenot$^{fl/fl}$* (n = 10) and *Selenot$^{fl/fl}$;Dat-cre* (n = 8) mice. (**C** and **D**) Familiar or novel object exploration time in short-term memory (1 h; **C**) and long-term memory (24 h; **D**) in the novel object recognition test of *Selenot$^{fl/fl}$* (n = 11) and *Selenot$^{fl/fl}$;Dat-cre* (n = 8) mice. (**E** and **F**) Latency to fall during 3-day training (**E**) and testing (**F**) in the rotarod task. n = 10 *Selenot$^{fl/fl}$* mice and n = 8 *Selenot$^{fl/fl}$;Dat-cre* mice. (**G**) Spontaneous alternation in the Y-maze test. n = 10 *Selenot$^{fl/fl}$* mice and n = 8 *Selenot$^{fl/fl}$;Dat-cre* mice. (**H-O**) Three chamber test of

Selenot$^{fl/fl}$ (n = 8) and *Selenot*$^{fl/fl}$*;Dat-cre* (n = 7) mice. Presented are the schematic diagram (**H** and **L**), representative activity heatmap (**I** and **M**), time spent near the empty cage or the cage with a stranger mouse (stranger 1; **J**), and the calculated social preference index (**K**), as well as time spent near the cage with the familiar mouse (stranger 1) or with another stranger (stranger 2; **N**) and the calculated social recognition index (**O**). (**P** and **Q**) Open field test of mice treated with saline or 1 mg/kg AMPH. n = 7 *Selenot*$^{fl/fl}$ + saline mice, 7 *Selenot*$^{fl/fl}$ + AMPH mice, 4 *Selenot*$^{fl/fl}$*;Dat-cre* + saline mice, and 4 *Selenot*$^{fl/fl}$*;Dat-cre* + AMPH mice. (**R** and **S**) Open field test of mice treated with saline or 15 mg/kg MPH. n = 5 *Selenot*$^{fl/fl}$ + saline mice, 6 *Selenot*$^{fl/fl}$ + MPH mice, 4 *Selenot*$^{fl/fl}$*;Dat-cre* + saline mice, and 4 *Selenot*$^{fl/fl}$*;Dat-cre* + MPH mice. Presented are locomotor activity every 10 min (**P** and **R**) and total distance over 60 min (**Q** and **S**). Data are presented as means ± SEM and analyzed by two-way repeated measures ANOVA for (**E**), factorial ANOVA for (**Q** and **S**), two-tailed paired *t*-test for (**C, D, J,** and **N**), and two-tailed unpaired *t*-test for other comparisons. AMPH**,** amphetamine; MPH, methylphenidate.

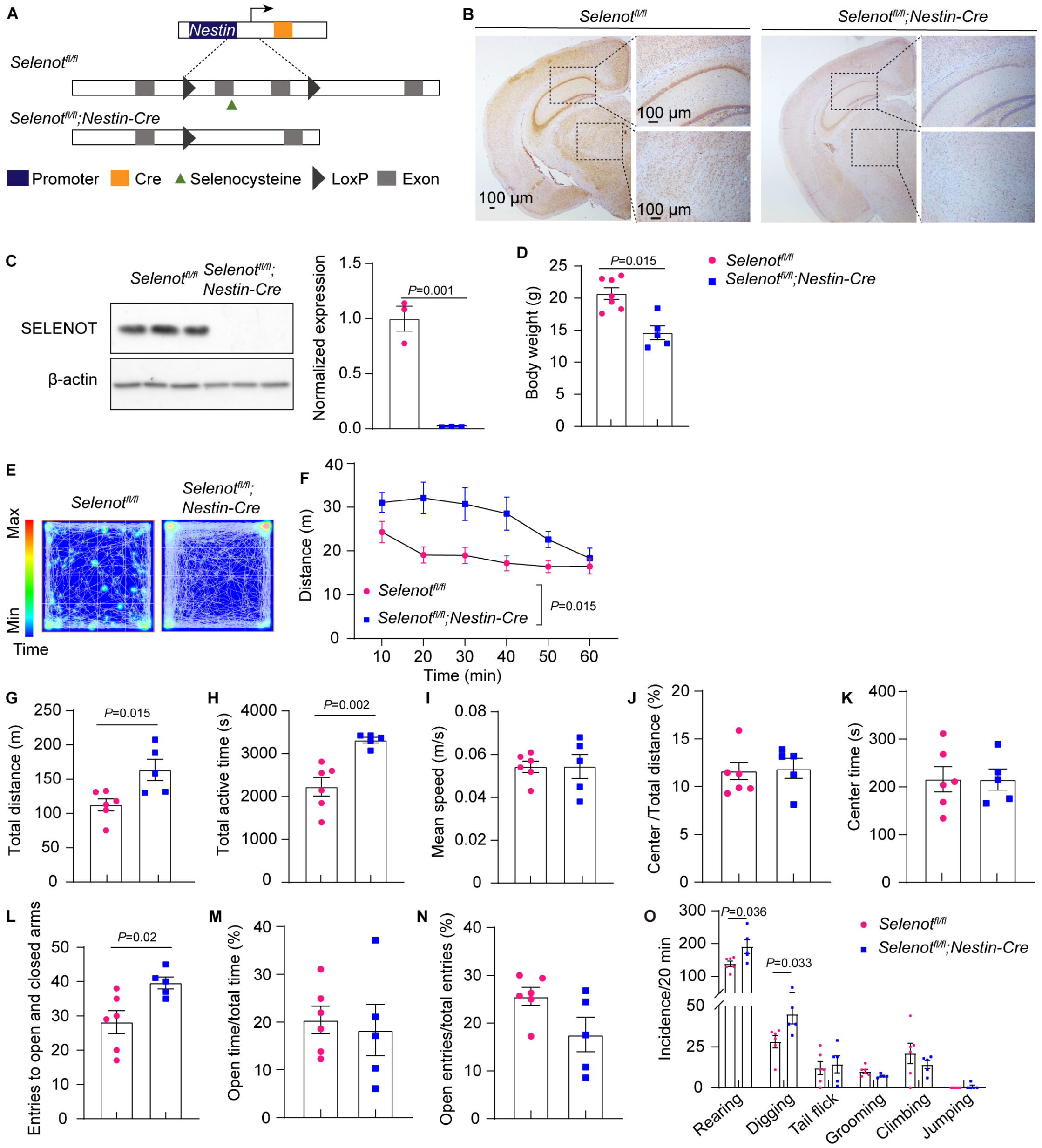

Appendix Figure S3. *Selenot$^{fl/fl}$;Nestin-cre* mice exhibit hyperactive behaviors. (**A**) Schematic diagram of *Nestin* promoter-driven excision of *Selenot* exons 2 and 3. (**B**) Immunohistochemistry analysis of SELENOT expression in the brain. (**C**) Western blot analysis and quantifications of SELENOT expression using the whole brain. Quantifications are normalized to β-actin. n = 3 mice for each genotype. (**D**) Body weight of mice at 8 weeks of age. n = 7 *Selenot$^{fl/fl}$* mice and n = 5 *Selenot$^{fl/fl}$;Nestin-cre* mice. (**E-K**) Open field test of *Selenot$^{fl/fl}$* (n = 6) and *Selenot$^{fl/fl}$;Nestin-cre* (n = 5) mice. Presented are representative activity heatmap (**E**), distance traveled every 10 min (**F**), total distance traveled (**G**), total active time (**H**), mean speed (**I**), percentage of center distance to total distance (**J**), and center time (**K**). (**L-N**) Elevated plus maze test of *Selenot$^{fl/fl}$* (n = 6) and *Selenot$^{fl/fl}$;Nestin-cre* (n = 5) mice. Presented are total entries to open and closed arms (**L**), the percentage of entries to open arms relative to total entries (**M**), and percentage of time spent in open arms relative to total time

spent in open and closed arms (**N**). (**O**) Stereotyped behaviors of *Selenot*$^{fl/fl}$ (n = 6) and *Selenot*$^{fl/fl}$*;Nestin-cre* (n = 5) mice. Data are presented as means ± SEM and analyzed by two-way repeated measures ANOVA for (**F**) and two-tailed unpaired *t*-test for other comparisons.

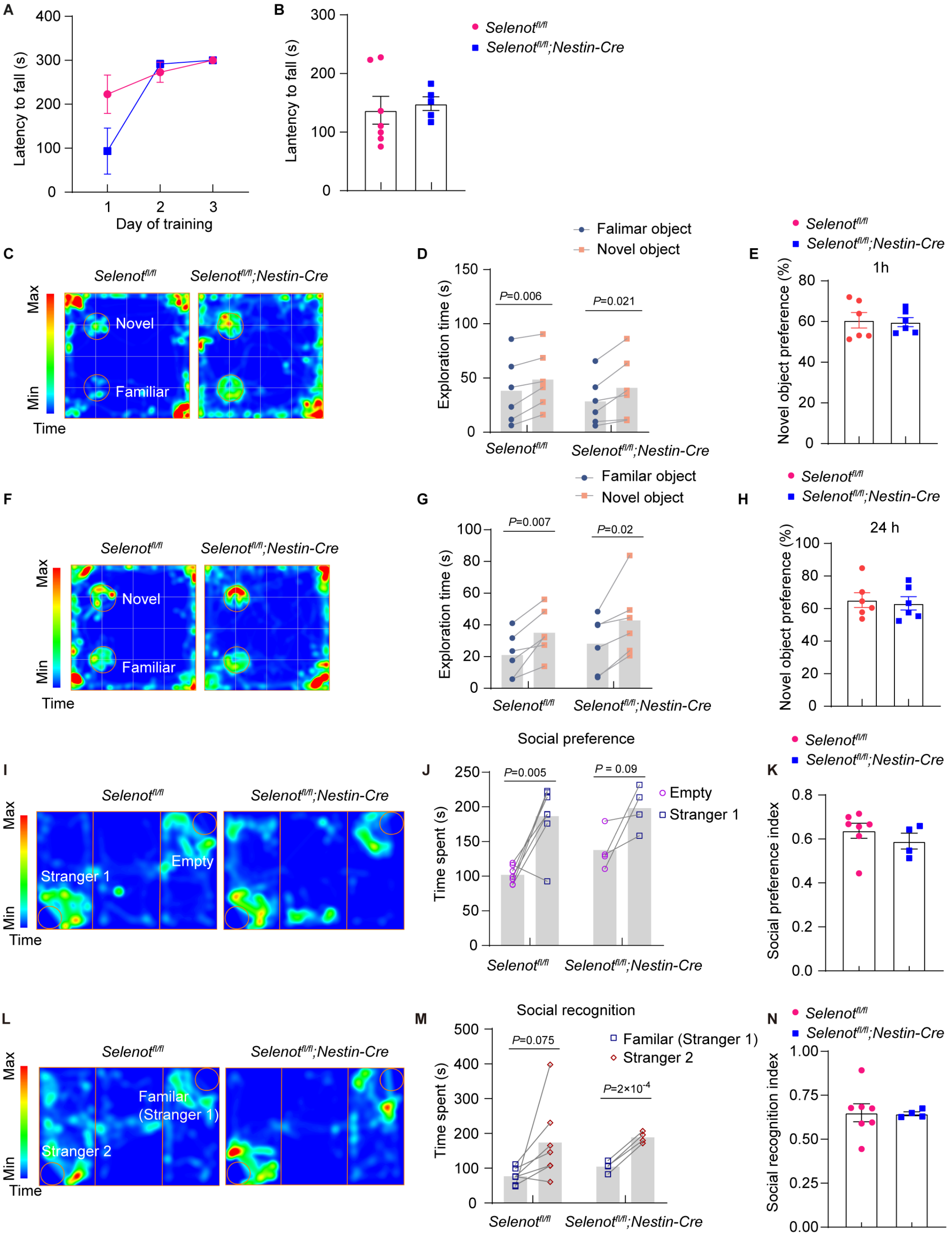

Appendix Figure S4. Additional behavioral assessments of *Selenot^fl/fl^;Nestin-cre* mice. (**A** and **B**) Latency to fall during 3-day training (**A**) and testing (**B**) in the rotarod. n = 7 *Selenot^fl/fl^* mice and n = 5 *Selenot^fl/fl^;Nestin-cre* mice. (**C-H**) Novel object recognition test of *Selenot^fl/fl^* (n = 6) and *Selenot^fl/fl^;Nestin-cre* (n = 6) mice. Presented are the representative activity heatmap, familiar or novel object exploration time, and the calculated novel object preference in short-term memory (1 h; **C-E**) and long-term memory (24 h; **F-H**). (**I-N**) Three chamber test of *Selenot^fl/fl^* (n = 7) and *Selenot^fl/fl^;Nestin-cre* (n = 4) mice. Presented are the representative activity heatmap (**I** and **L**), time spent near the cage empty or with a stranger mouse (stranger

1; **J**) and the calculated social preference index (**K**), and time spent near the cage with the familiar mouse (stranger 1) or with another stranger (stranger 2; **M**) and the calculated social recognition index (**N**). Note: the cages were unintentionally placed in contralateral corners for this genotype. Data are presented as means ± SEM and analyzed by two-way repeated measures ANOVA for (**A**), two-tailed paired *t*-test for (**D, G, J** and **M**), and two-tailed unpaired *t*-test for other comparisons.

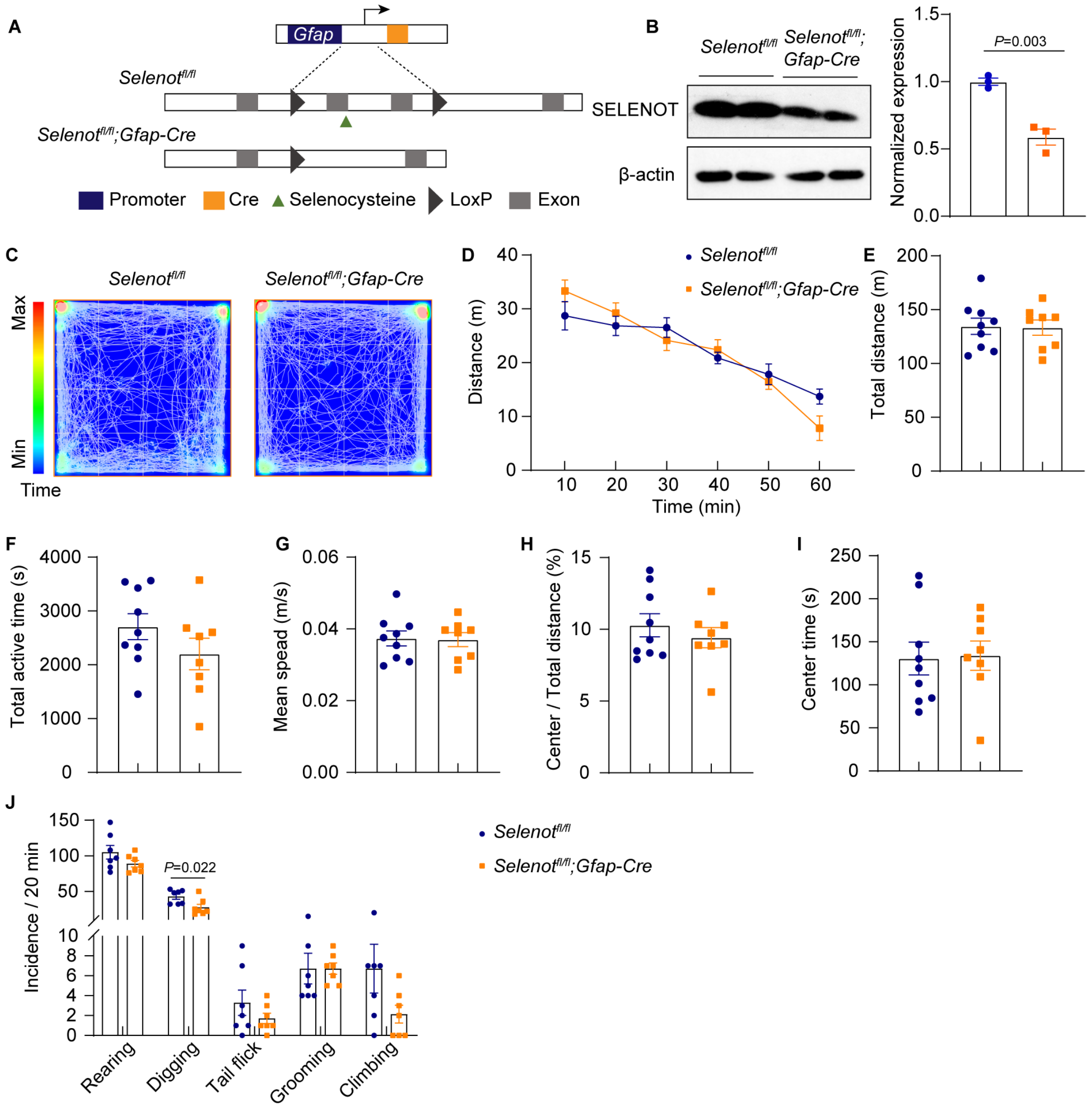

Appendix Figure S5. Generation of *Selenot$^{fl/fl}$;Gfap-cre* mice and the locomotion behavior assessments. (**A**) Schematic diagram of *Gfap* promoter-driven excision of *Selenot* exons 2 and 3. (**B**) Western blot and quantifications of SELENOT expression using the whole brain. n = 3 *Selenot$^{fl/fl}$* mice and n = 3 *Selenot$^{fl/fl}$;Gfap-cre* mice. Quantifications are normalized to β-actin. (**C-I**) Open field test of *Selenot$^{fl/fl}$* (n = 9) and *Selenot$^{fl/fl}$;Gfap-cre* (n = 8) mice. Presented are representative activity heatmap (**C**), distance traveled every 10 min (**D**), total distance traveled (**E**), total active time (**F**), mean speed (**G**), percentage of center distance to total distance (**H**), and center time (**I**). (**J**) Stereotyped behaviors of *Selenot$^{fl/fl}$* (n = 7) and *Selenot$^{fl/fl}$;Gfap-cre* (n = 7) mice. Data are presented as means ± SEM and analyzed by two-way repeated measures ANOVA for (**D**) and two-tailed unpaired *t*-test for other comparisons.

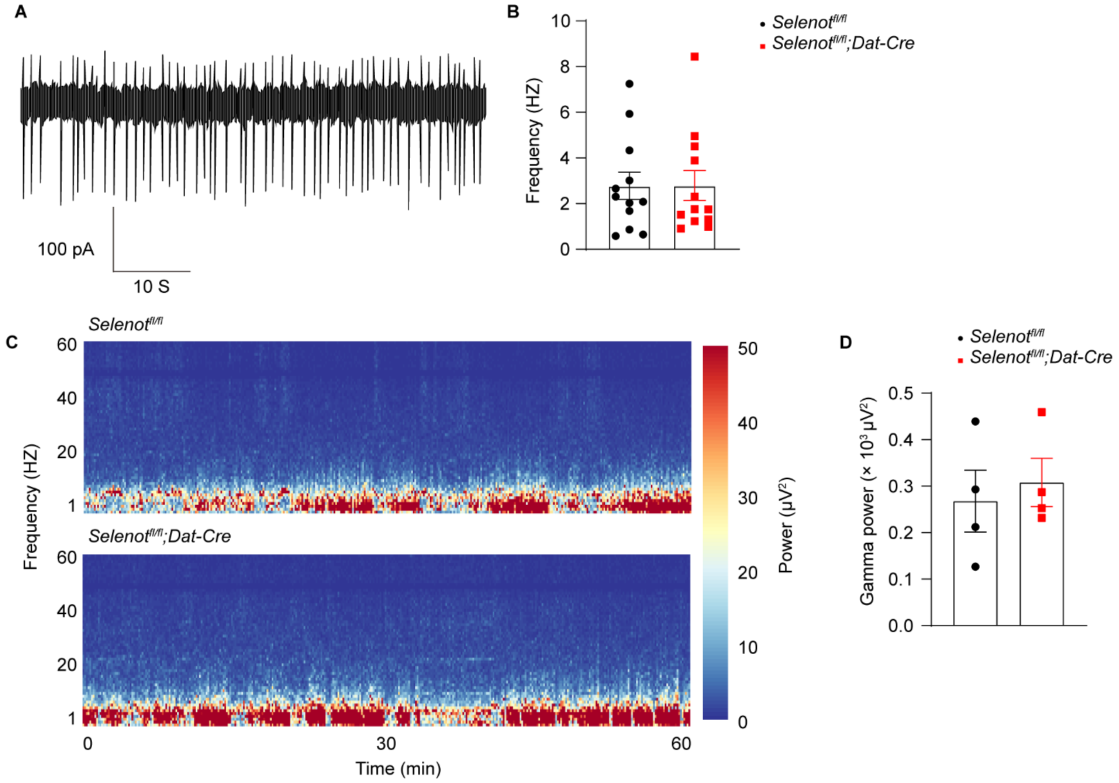

Appendix Figure S6. Pacemaker of dopaminergic neurons and higher frequency EEG power in occipital regions. (**A** and **B**) Representative traces (**A**) and pacemaker frequencies (**B**) of dopaminergic neurons from cell-attached recordings. n = 12 neurons from 3 *Selenot*$^{fl/fl}$ mice and n = 12 neurons from 3 *Selenot*$^{fl/fl}$*;Dat-cre* mice. (**C** and **D**) Representative spectrograms (**C**) and gamma power (27-44 Hz; **D**) over 60 min in occipital regions. n = 4 *Selenot*$^{fl/fl}$ mice and n = 4 *Selenot*$^{fl/fl}$*;Dat-cre* mice. Data are presented as means ± SEM and analyzed by two-tailed unpaired *t*-test. EEG, electroencephalogram.

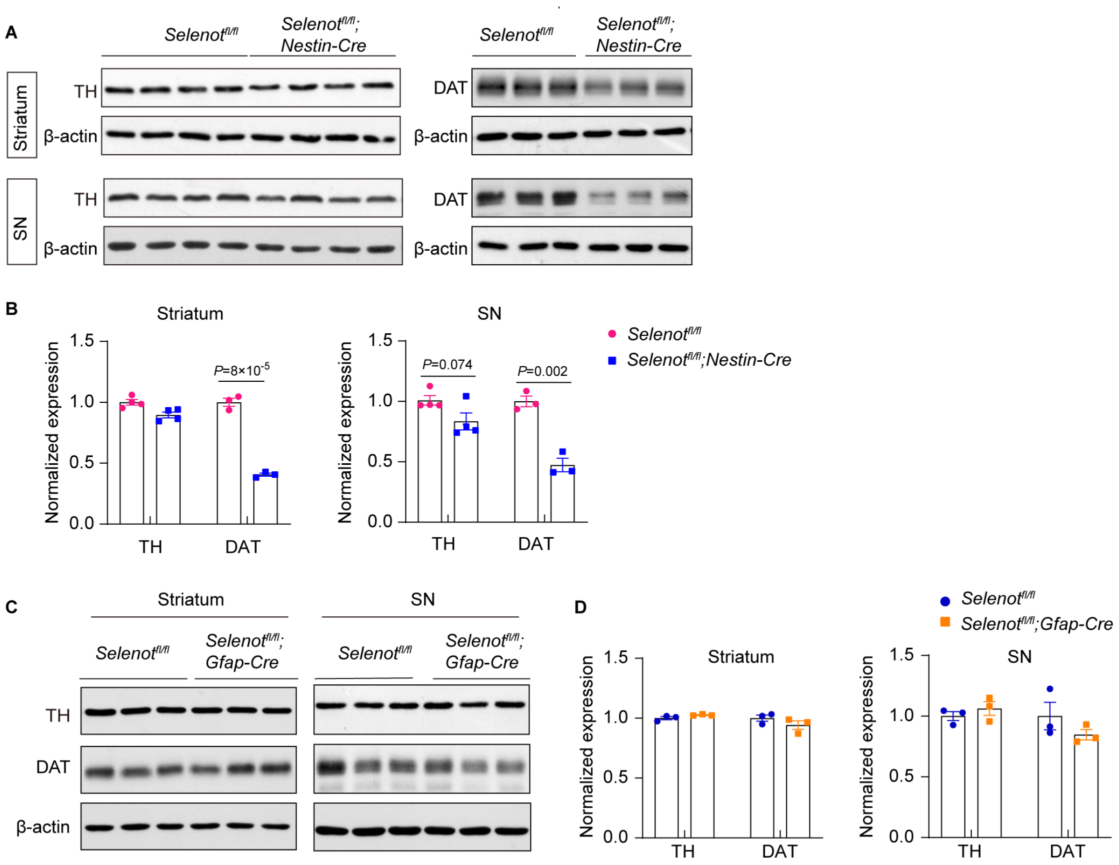

Appendix Figure S7. Analyses of TH and DAT expression between *Selenot*$^{fl/fl}$ and *Selenot*$^{fl/fl}$*;Nestin-cre* or *Selenot*$^{fl/fl}$*;Gfap-cre* mice. (**A** and **B**) Western blot analysis (**A**) and quantifications (**B**) of TH and DAT expression in the whole striatum and substantia nigra of *Selenot*$^{fl/fl}$ (n = 3-4) and *Selenot*$^{fl/fl}$*;Nestin-cre* (n = 3-4) mice. (**C** and **D**) Western blot analyses (**C**) and quantifications (**D**) of TH and DAT expression in the whole striatum and substantia nigra of *Selenot*$^{fl/fl}$ (n = 3) and *Selenot*$^{fl/fl}$*;Gfap-cre* (n = 3) mice. Quantifications for Western blot are normalized to β-actin. Data are presented as means ± SEM and analyzed by two-tailed unpaired *t*-test. DAT, dopamine transporter; SN, substantia nigra; TH, tyrosine hydroxylase.

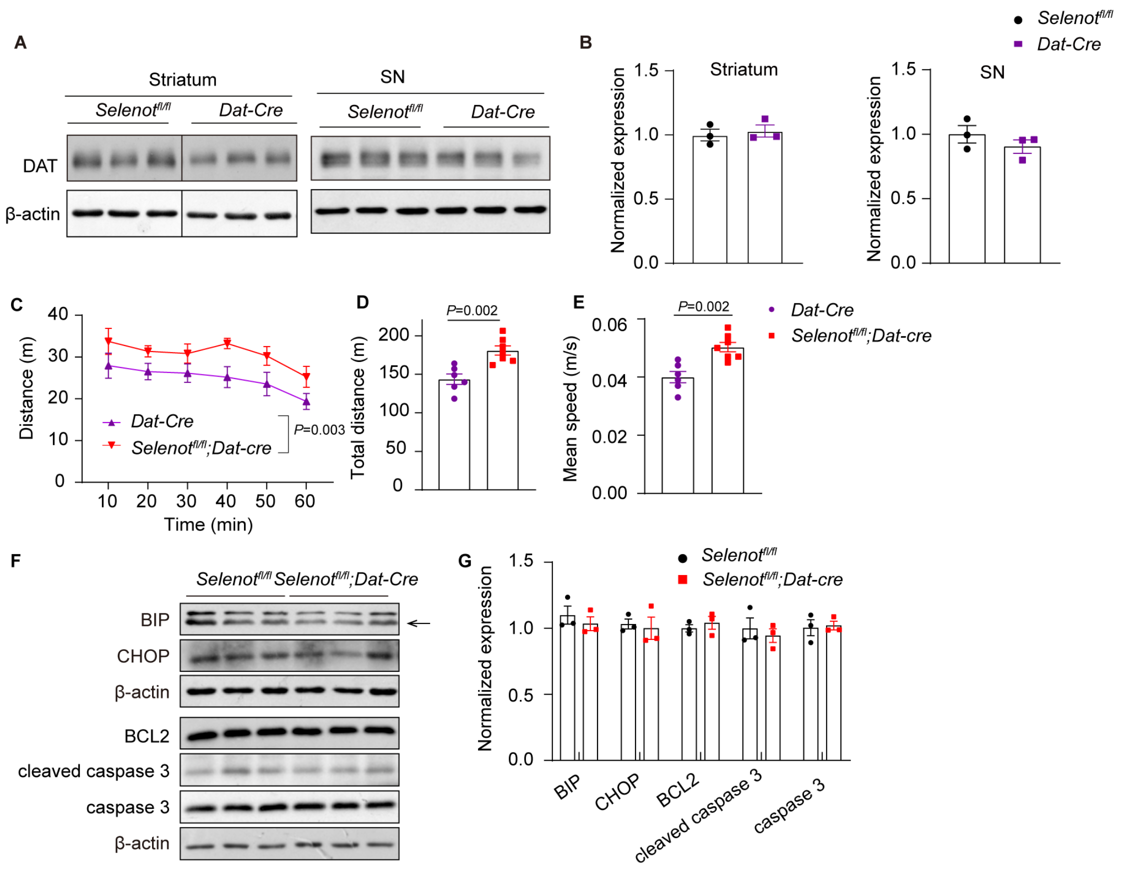

Appendix Figure S8. Analyses of DAT expression and locomotion between *Selenot$^{fl/fl}$* and *Dat-cre* mice, and apoptosis and ER stress in *Selenot$^{fl/fl}$;Dat-cre* mice. (**A** and **B**) Western blot analyses (**A**) and quantifications (**B**) of DAT expression in the whole striatum and substantia nigra of *Selenot$^{fl/fl}$* mice (n = 3) and *Dat-cre* mice (n =3). Lanes are neighbored from one blot for the striatum. (**C-E**) Open field test of *Dat-cre* (n = 6) and *Selenot$^{fl/fl}$;Dat-cre* (n = 7) mice. Presented are distance traveled every 10 min (**C**), total distance traveled (**D**), and mean speed (**E**). (**F** and **G**) Western blot analyses (**F**) and quantifications (**G**) of proteins associated with apoptosis and ER stress in the substantia nigra of *Selenot$^{fl/fl}$* mice (n = 3) and *Selenot$^{fl/fl}$;Dat-cre* mice (n = 3). The arrow indicates the band for BIP. Quantifications are normalized to β-actin. Data are presented as means ± SEM and analyzed by two-way repeated measures ANOVA for (**C**) and two-tailed unpaired *t*-test for other comparisons. BCL-2, B-cell lymphoma-2; BIP, binding-immunoglobulin protein; CHOP, CCAAT-enhancer-binding protein homologous protein; DAT, dopamine transporter; ER, endoplasmic reticulum; SN, substantia nigra.

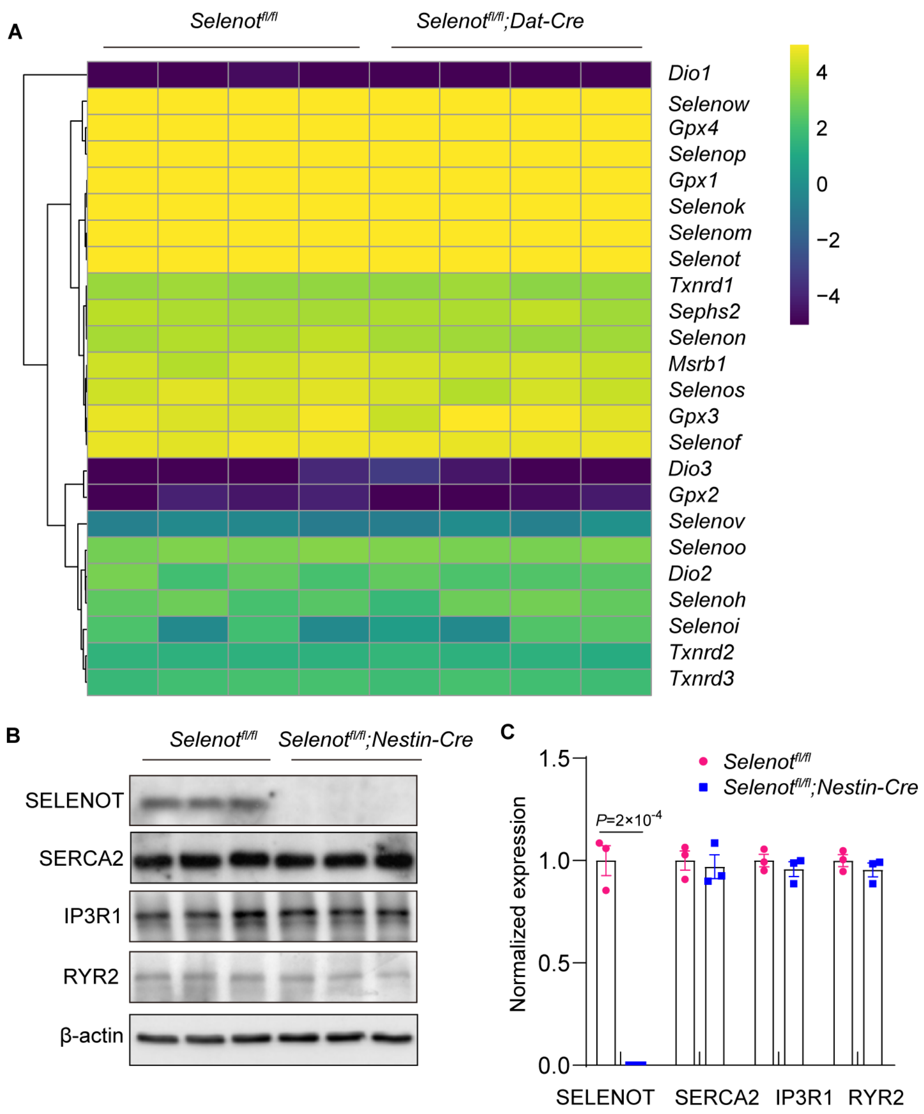

Appendix Figure S9. RNA-seq results of selenoprotein expression and Western blot analyses for SERCA2, IP3R1, and RYR2 expression. (**A**) Row-normalized RNA-seq heatmap for 24 selenoprotein genes in the substantia nigra of *Selenot*$^{fl/fl}$ and *Selenot*$^{fl/fl}$*;Dat-cre mice.* n = 4. (**B** and **C**) Western blot analyses (**B**) and quantifications (**C**) of SERCA2, IP3R1, and RYR2 in the cortex of *Selenot*$^{fl/fl}$ mice (n = 3) and *Selenot*$^{fl/fl}$*;Nestin-cre* mice (n = 3). Quantifications are normalized to β-actin. Data are presented as means ± SEM and analyzed by two-tailed unpaired *t*-test. IP3R1, inositol 1,4,5-triphosphate receptor, type 1; RYR2, ryanodine receptor 2; SERCA2, sarco-ER Ca^{2+} ATPase 2.

Appendix Table S1. Sequencing data quality

Samples	Raw reads[a]	Raw bases[a]	Valid reads[b]	Valid bases[b]	Valid ratio[c]	Q20[d]	Q30[d]	GC content[e]
Ctrl_1	49031804	7.35G	47240790	7.09G	96.35%	99.96%	98.11%	49%
Ctrl_2	45395360	6.81G	43781104	6.57G	96.44%	99.96%	98.12%	49%
Ctrl_3	48307540	7.25G	45833744	6.88G	94.88%	99.97%	98.23%	49%
Ctrl_4	48102128	7.22G	46599432	6.99G	96.88%	99.97%	98.67%	50%
cKO_1	48343630	7.25G	46445512	6.97G	96.07%	99.96%	98.05%	49%
cKO_2	46833148	7.02G	45207480	6.78G	96.53%	99.96%	98.14%	49%
cKO_3	44450204	6.67G	42823046	6.42G	96.34%	99.96%	98.03%	49%
cKO_4	47291838	7.09G	45256346	6.79G	95.70%	99.96%	98.03%	49%

[a] Number of total reads and bases (G) in the original sequencing data;

[b] Number of reads and bases (G) after quality control;

[c] Ratio of valid reads to raw reads;

[d] Proportion of bases with a quality score over 20 or 30. The error probability of each base during base calling was provided by the sequencing system. The quality score of each base was calculated by -10lgP. If the error probability of a base was 0.01, its quality score was Q20; if the error probability of one base was 0.001, its quality score was Q30.

[e] Ratio of GC to total bases in valid bases.
