# Supplementary figures and images for: The selenocysteine-containing protein SELENOT maintains dopamine signaling in the midbrain to protect mice from hyperactivity disorder
Source: EMBO J. 2025 Apr 7;44(10):2906–27. doi: 10.1038/s44318-025-00430-3 (PMC12084338; doi:10.1038/s44318-025-00430-3)

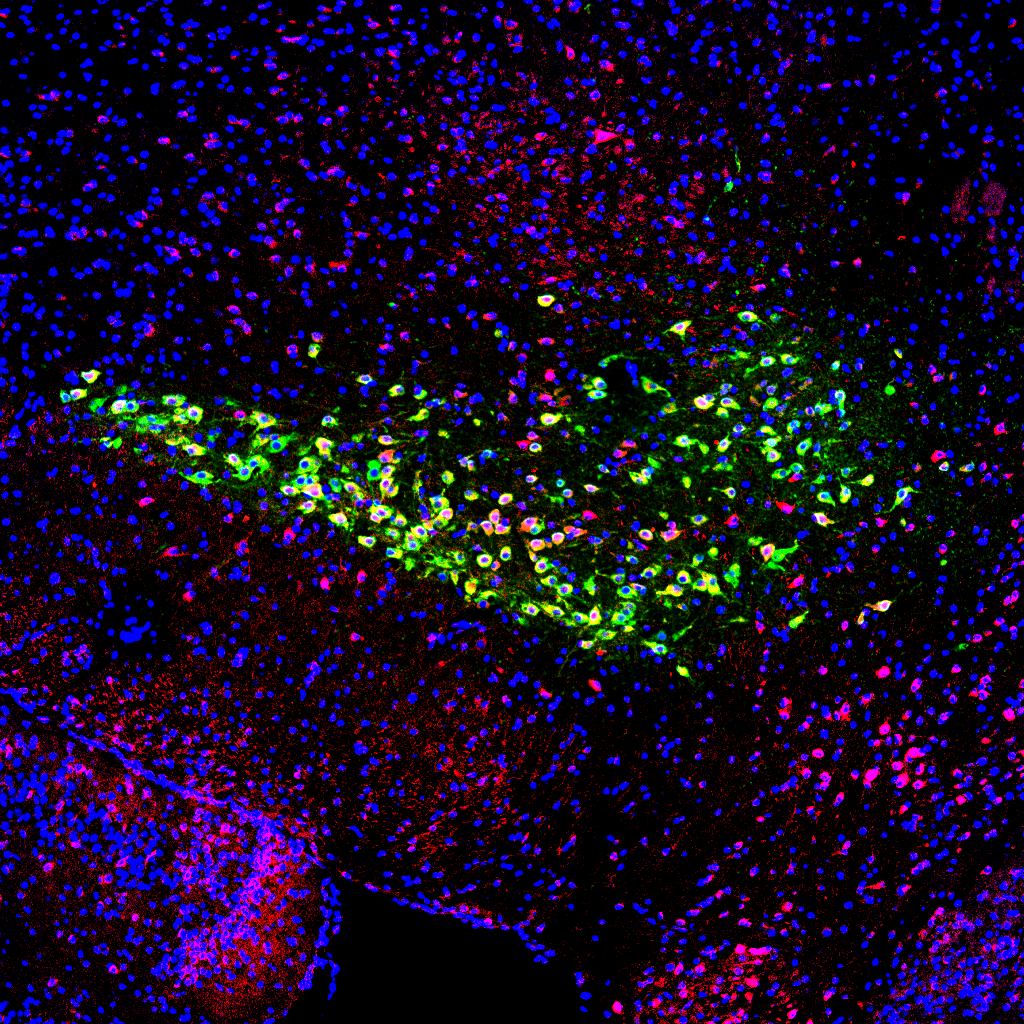

Supplement: Supplementary file 4 — Source data Fig. 1 [file 44318_2025_430_MOESM4_ESM.zip › Figure 1/Figure 1B/Selenotfl fl-Merge.tif]

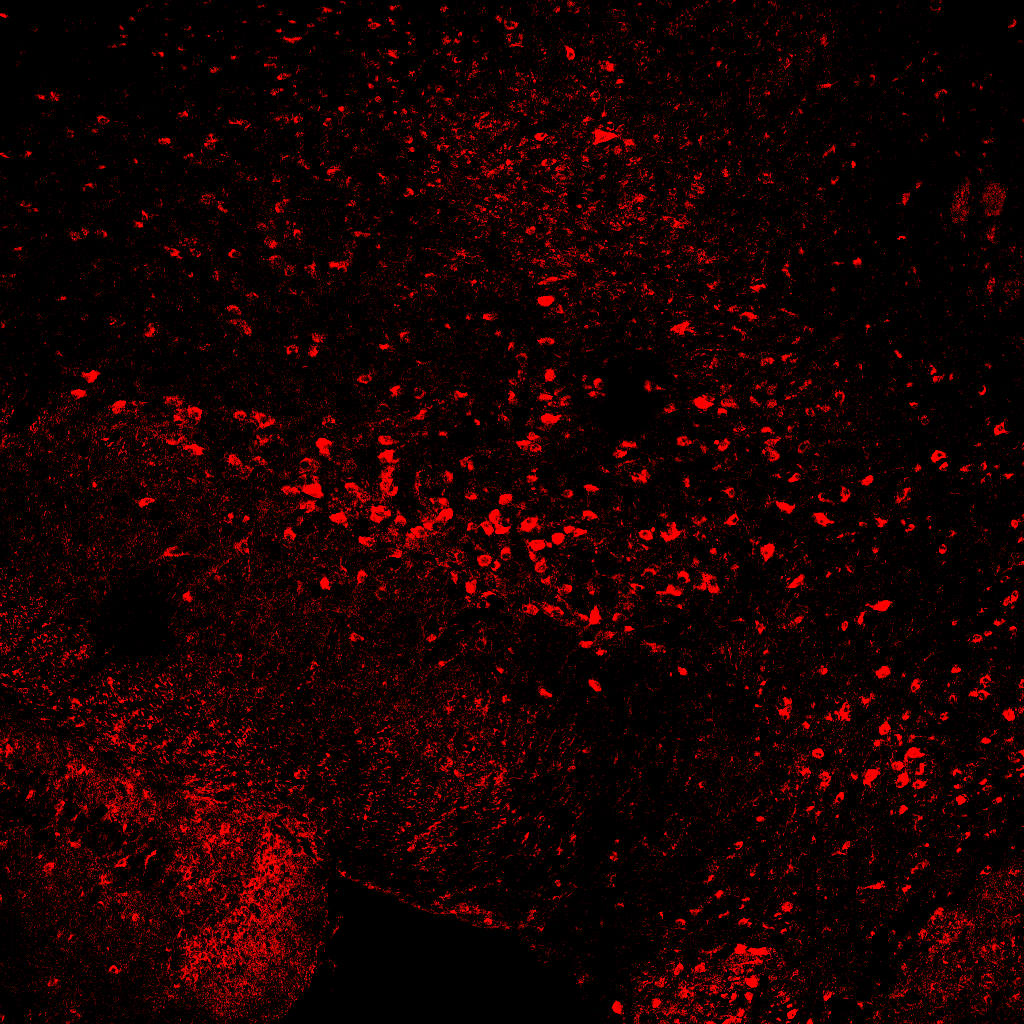

Supplement: Supplementary file 4 — Source data Fig. 1 [file 44318_2025_430_MOESM4_ESM.zip › Figure 1/Figure 1B/Selenotfl fl-SEELNOT.tif]

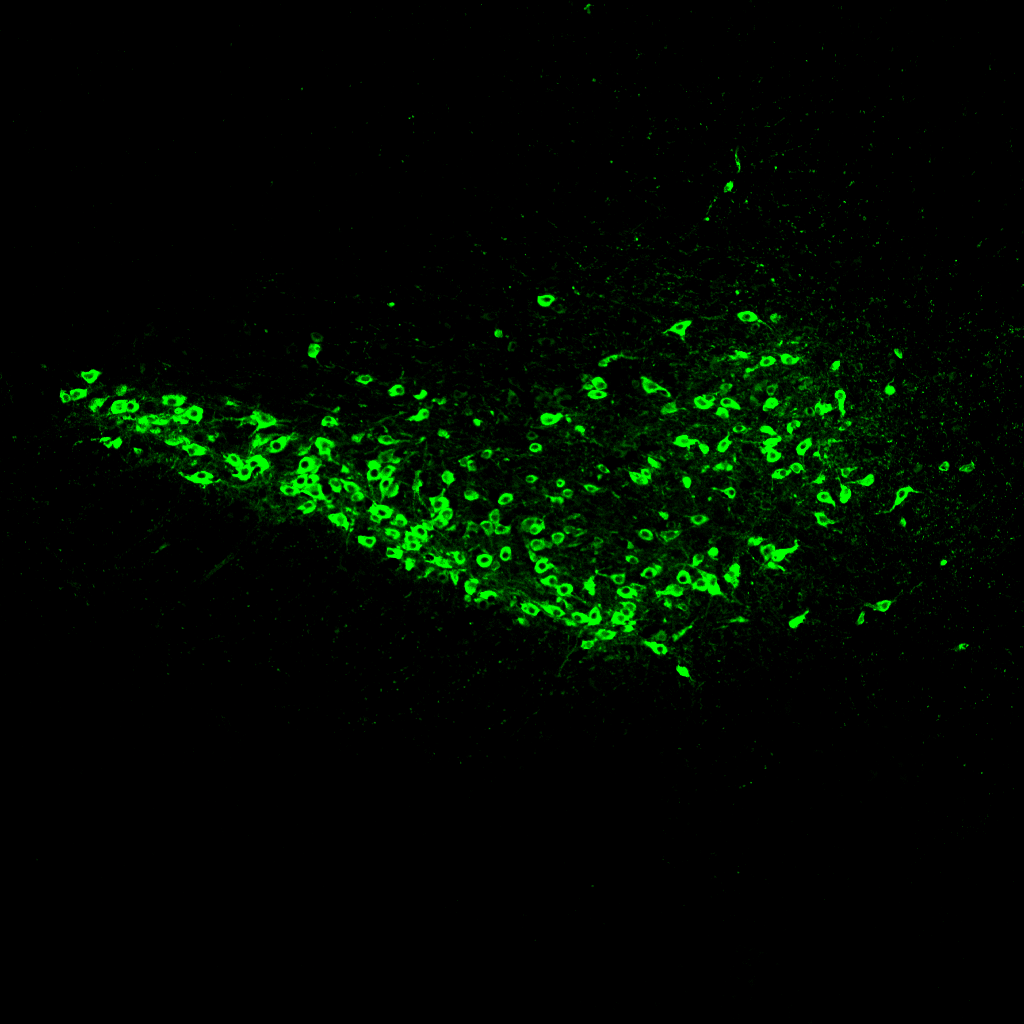

Supplement: Supplementary file 4 — Source data Fig. 1 [file 44318_2025_430_MOESM4_ESM.zip › Figure 1/Figure 1B/Selenotfl fl-TH.tif]

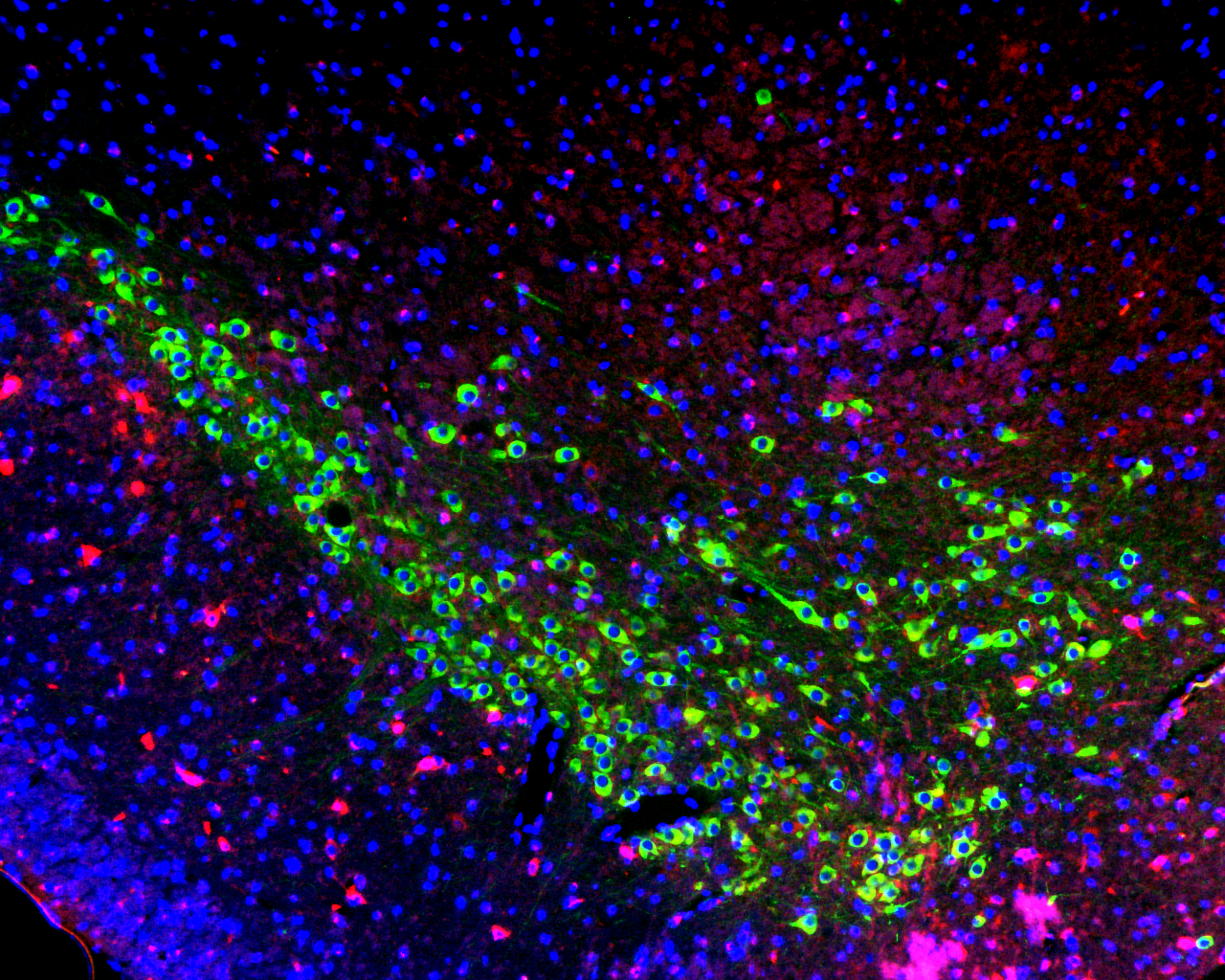

Supplement: Supplementary file 4 — Source data Fig. 1 [file 44318_2025_430_MOESM4_ESM.zip › Figure 1/Figure 1B/Selenotflfl Dat-Cre Merge.tif]

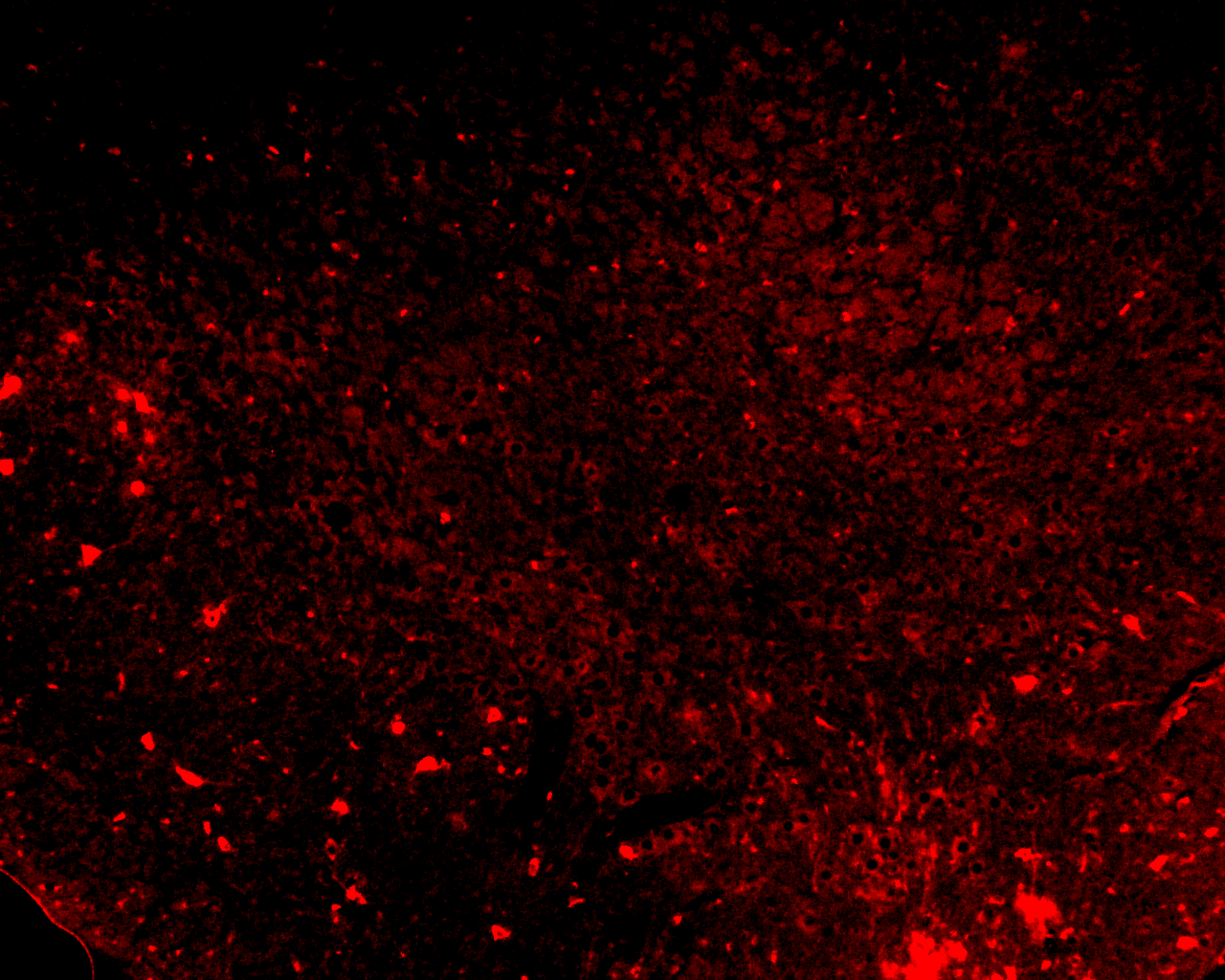

Supplement: Supplementary file 4 — Source data Fig. 1 [file 44318_2025_430_MOESM4_ESM.zip › Figure 1/Figure 1B/Selenotflfl Dat-Cre SELENOT.tif]

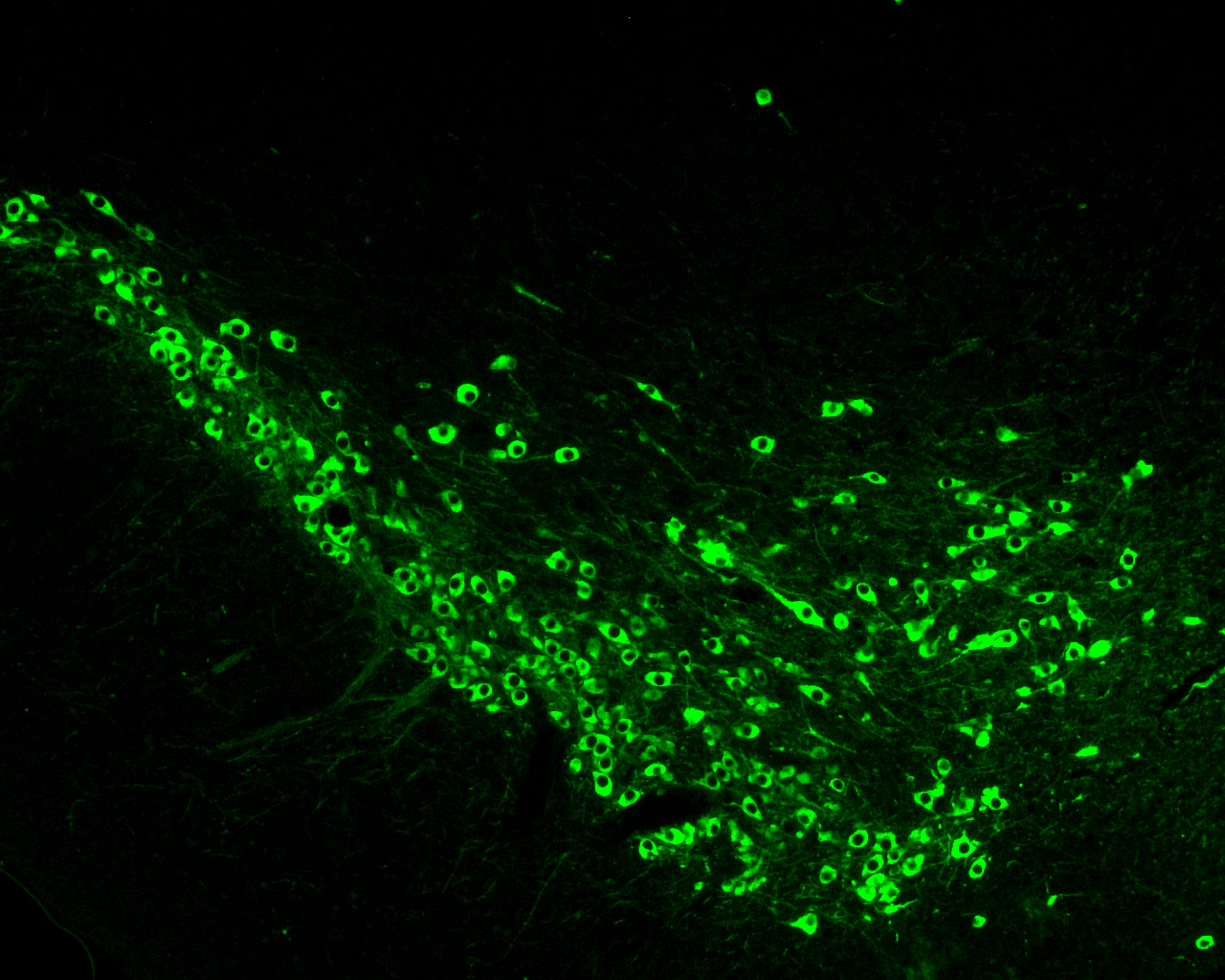

Supplement: Supplementary file 4 — Source data Fig. 1 [file 44318_2025_430_MOESM4_ESM.zip › Figure 1/Figure 1B/Selenotflfl Dat-Cre TH.tif]

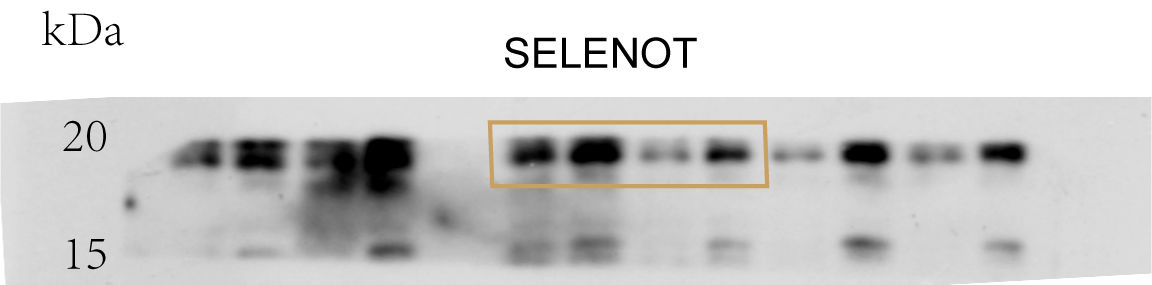

Supplement: Supplementary file 4 — Source data Fig. 1 [file 44318_2025_430_MOESM4_ESM.zip › Figure 1/Figure 1Y/SELENOT.tif]

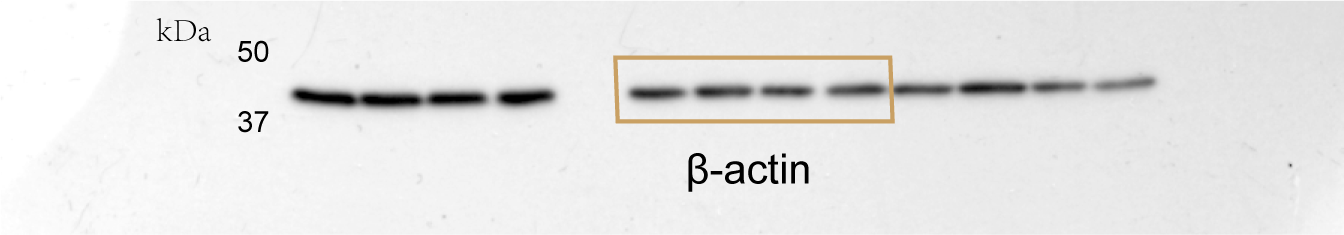

Supplement: Supplementary file 4 — Source data Fig. 1 [file 44318_2025_430_MOESM4_ESM.zip › Figure 1/Figure 1Y/a┬-actin.tif]

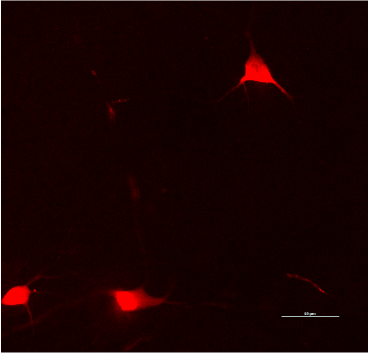

Supplement: Supplementary file 6 — Source data Fig. 3 [file 44318_2025_430_MOESM6_ESM.zip › Figure 3/Figure 3A/Biocytin.tif]

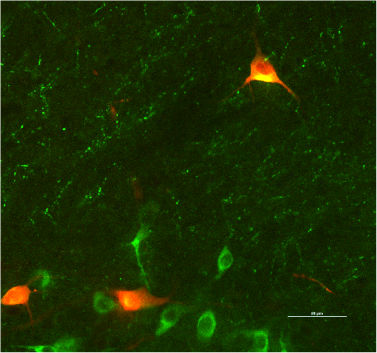

Supplement: Supplementary file 6 — Source data Fig. 3 [file 44318_2025_430_MOESM6_ESM.zip › Figure 3/Figure 3A/Merge.tif]

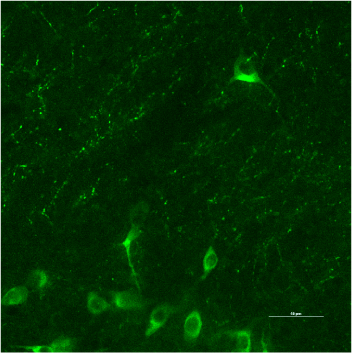

Supplement: Supplementary file 6 — Source data Fig. 3 [file 44318_2025_430_MOESM6_ESM.zip › Figure 3/Figure 3A/TH.tif]

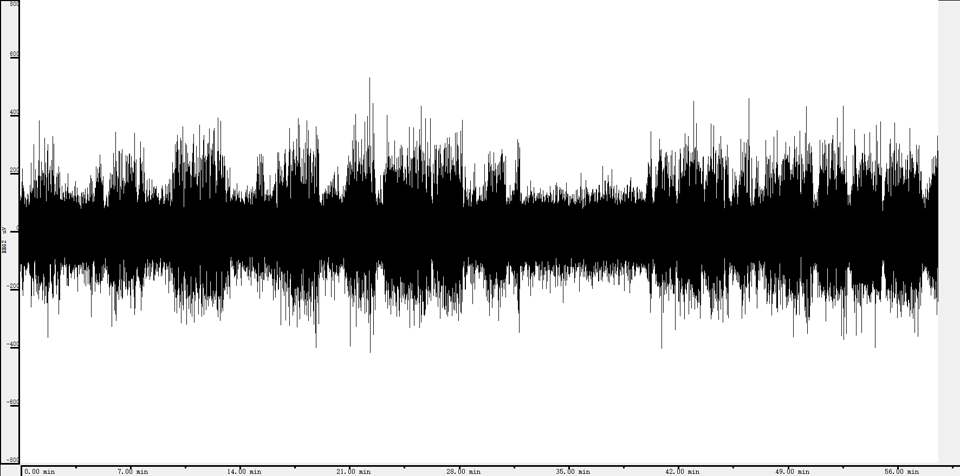

Supplement: Supplementary file 6 — Source data Fig. 3 [file 44318_2025_430_MOESM6_ESM.zip › Figure 3/Figure3I/selenotflfl Dat-cre.tif]

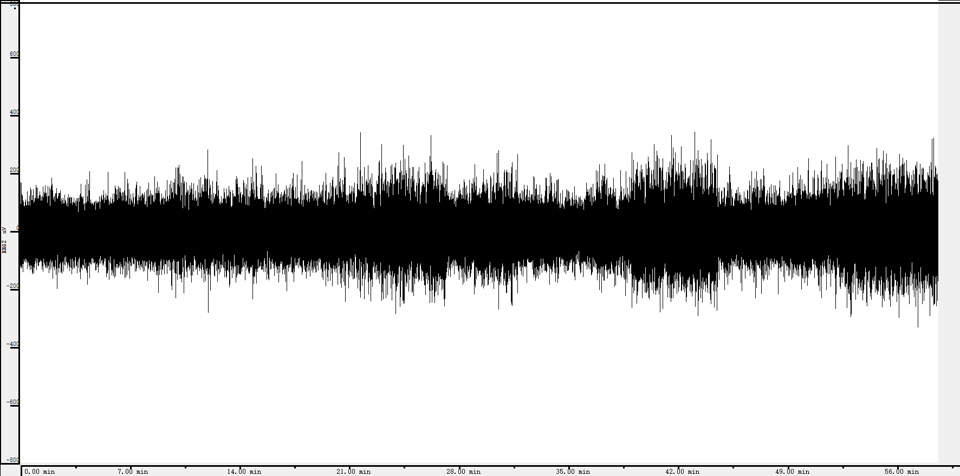

Supplement: Supplementary file 6 — Source data Fig. 3 [file 44318_2025_430_MOESM6_ESM.zip › Figure 3/Figure3I/selenotflfl.tif]

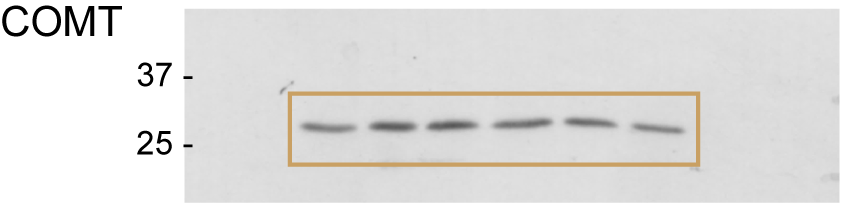

Supplement: Supplementary file 7 — Source data Fig. 4 [file 44318_2025_430_MOESM7_ESM.zip › Figure 4/Figure 4A/Dorsal striatum COMT.tif]

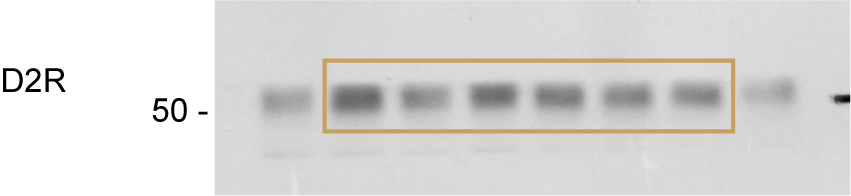

Supplement: Supplementary file 7 — Source data Fig. 4 [file 44318_2025_430_MOESM7_ESM.zip › Figure 4/Figure 4A/Dorsal striatum D2R.tif]

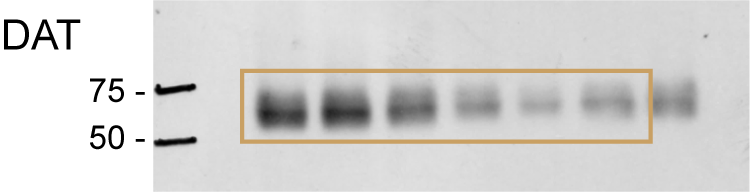

Supplement: Supplementary file 7 — Source data Fig. 4 [file 44318_2025_430_MOESM7_ESM.zip › Figure 4/Figure 4A/Dorsal striatum DAT.tif]

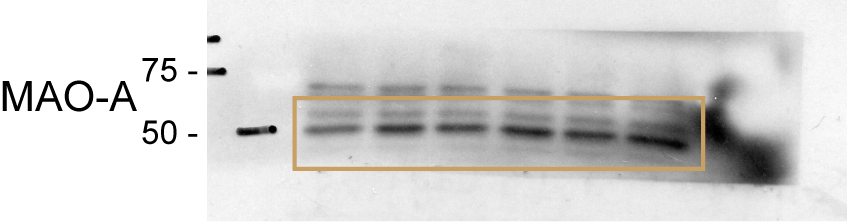

Supplement: Supplementary file 7 — Source data Fig. 4 [file 44318_2025_430_MOESM7_ESM.zip › Figure 4/Figure 4A/Dorsal striatum MAO-A.tif]

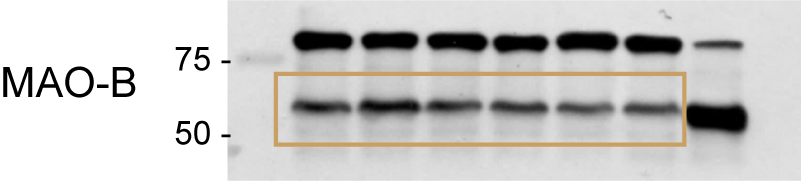

Supplement: Supplementary file 7 — Source data Fig. 4 [file 44318_2025_430_MOESM7_ESM.zip › Figure 4/Figure 4A/Dorsal striatum MAO-B.tif]

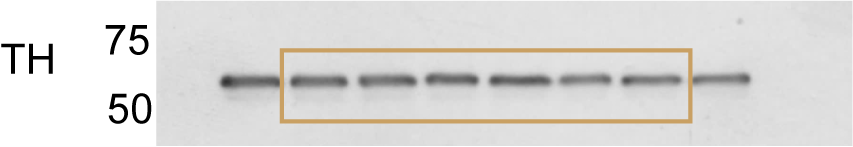

Supplement: Supplementary file 7 — Source data Fig. 4 [file 44318_2025_430_MOESM7_ESM.zip › Figure 4/Figure 4A/Dorsal striatum TH.tif]

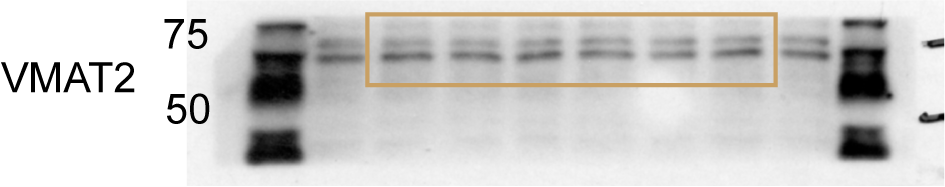

Supplement: Supplementary file 7 — Source data Fig. 4 [file 44318_2025_430_MOESM7_ESM.zip › Figure 4/Figure 4A/Dorsal striatum VMAT2.tif]

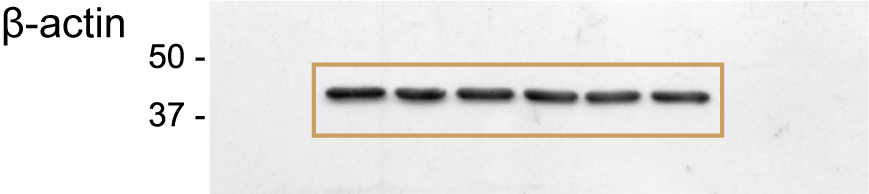

Supplement: Supplementary file 7 — Source data Fig. 4 [file 44318_2025_430_MOESM7_ESM.zip › Figure 4/Figure 4A/Dorsal striatum a┬-actin for MAO-A COMT.tif]

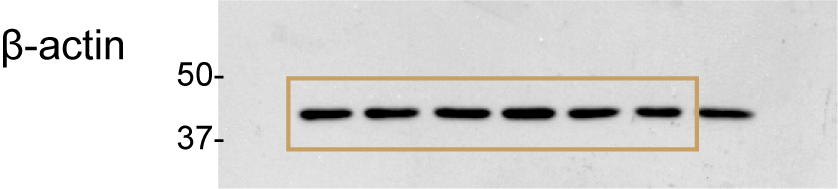

Supplement: Supplementary file 7 — Source data Fig. 4 [file 44318_2025_430_MOESM7_ESM.zip › Figure 4/Figure 4A/Dorsal striatum a┬-actin for DAT MAO-B.tif]

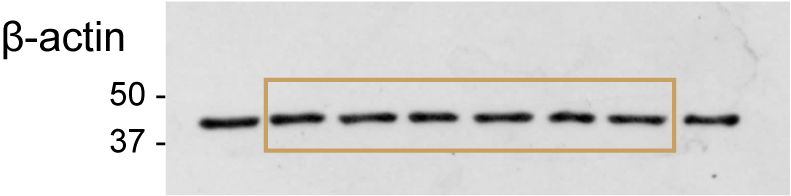

Supplement: Supplementary file 7 — Source data Fig. 4 [file 44318_2025_430_MOESM7_ESM.zip › Figure 4/Figure 4A/Dorsal striatum a┬-actin for TH VMAT2 D2R.tif]

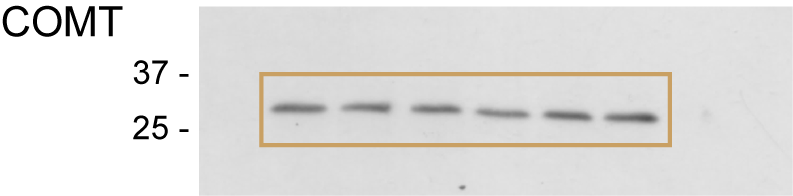

Supplement: Supplementary file 7 — Source data Fig. 4 [file 44318_2025_430_MOESM7_ESM.zip › Figure 4/Figure 4A/Ventral striatum COMT.tif]

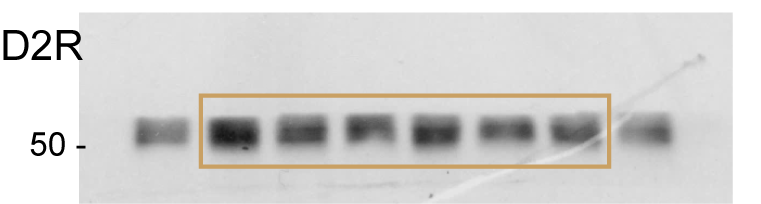

Supplement: Supplementary file 7 — Source data Fig. 4 [file 44318_2025_430_MOESM7_ESM.zip › Figure 4/Figure 4A/Ventral striatum D2R.tif]

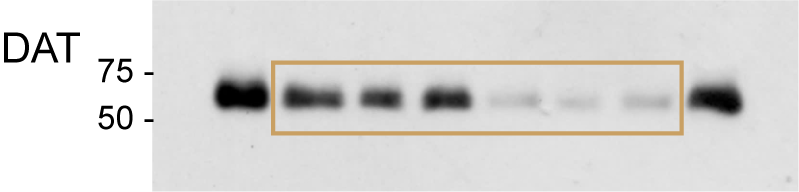

Supplement: Supplementary file 7 — Source data Fig. 4 [file 44318_2025_430_MOESM7_ESM.zip › Figure 4/Figure 4A/Ventral striatum DAT.tif]

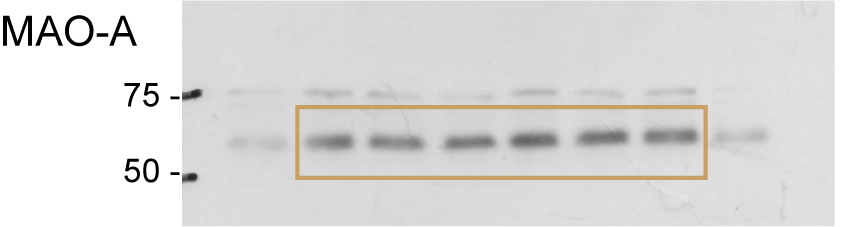

Supplement: Supplementary file 7 — Source data Fig. 4 [file 44318_2025_430_MOESM7_ESM.zip › Figure 4/Figure 4A/Ventral striatum MAO-A.tif]

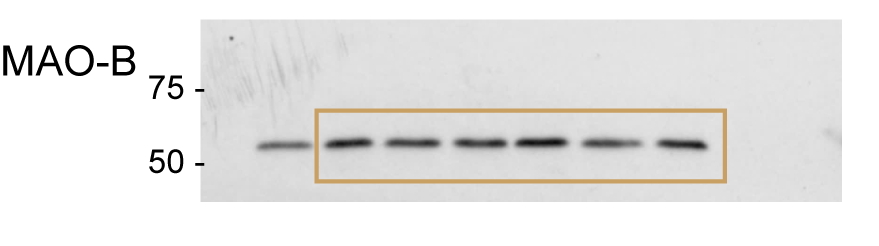

Supplement: Supplementary file 7 — Source data Fig. 4 [file 44318_2025_430_MOESM7_ESM.zip › Figure 4/Figure 4A/Ventral striatum MAO-B.tif]

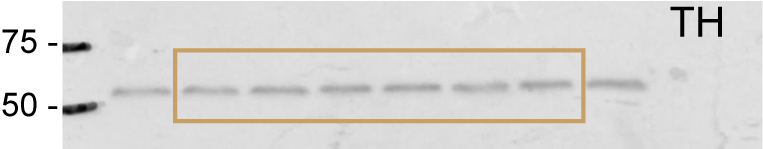

Supplement: Supplementary file 7 — Source data Fig. 4 [file 44318_2025_430_MOESM7_ESM.zip › Figure 4/Figure 4A/Ventral striatum TH.tif]

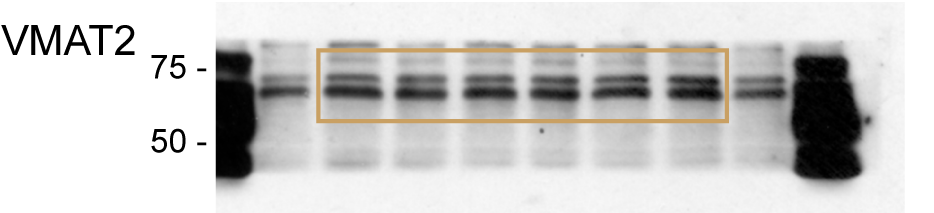

Supplement: Supplementary file 7 — Source data Fig. 4 [file 44318_2025_430_MOESM7_ESM.zip › Figure 4/Figure 4A/Ventral striatum VMAT2.tif]

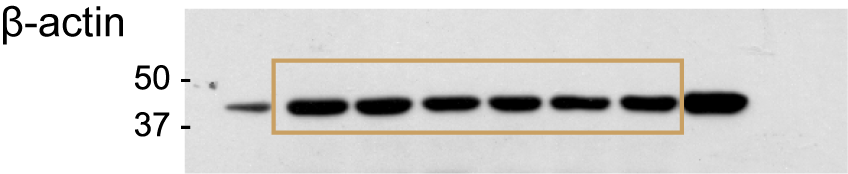

Supplement: Supplementary file 7 — Source data Fig. 4 [file 44318_2025_430_MOESM7_ESM.zip › Figure 4/Figure 4A/Ventral striatum a┬-actin for MAO-A DAT .tif]

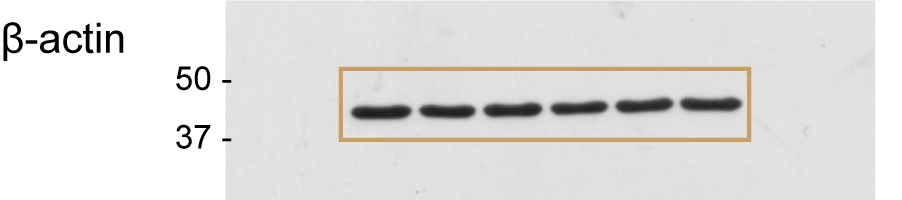

Supplement: Supplementary file 7 — Source data Fig. 4 [file 44318_2025_430_MOESM7_ESM.zip › Figure 4/Figure 4A/Ventral striatum a┬-actin for MAO-B COMT.tif]

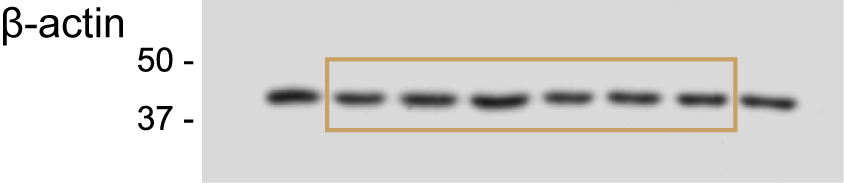

Supplement: Supplementary file 7 — Source data Fig. 4 [file 44318_2025_430_MOESM7_ESM.zip › Figure 4/Figure 4A/Ventral striatum a┬-actin forTH VMAT2 D2R.tif]

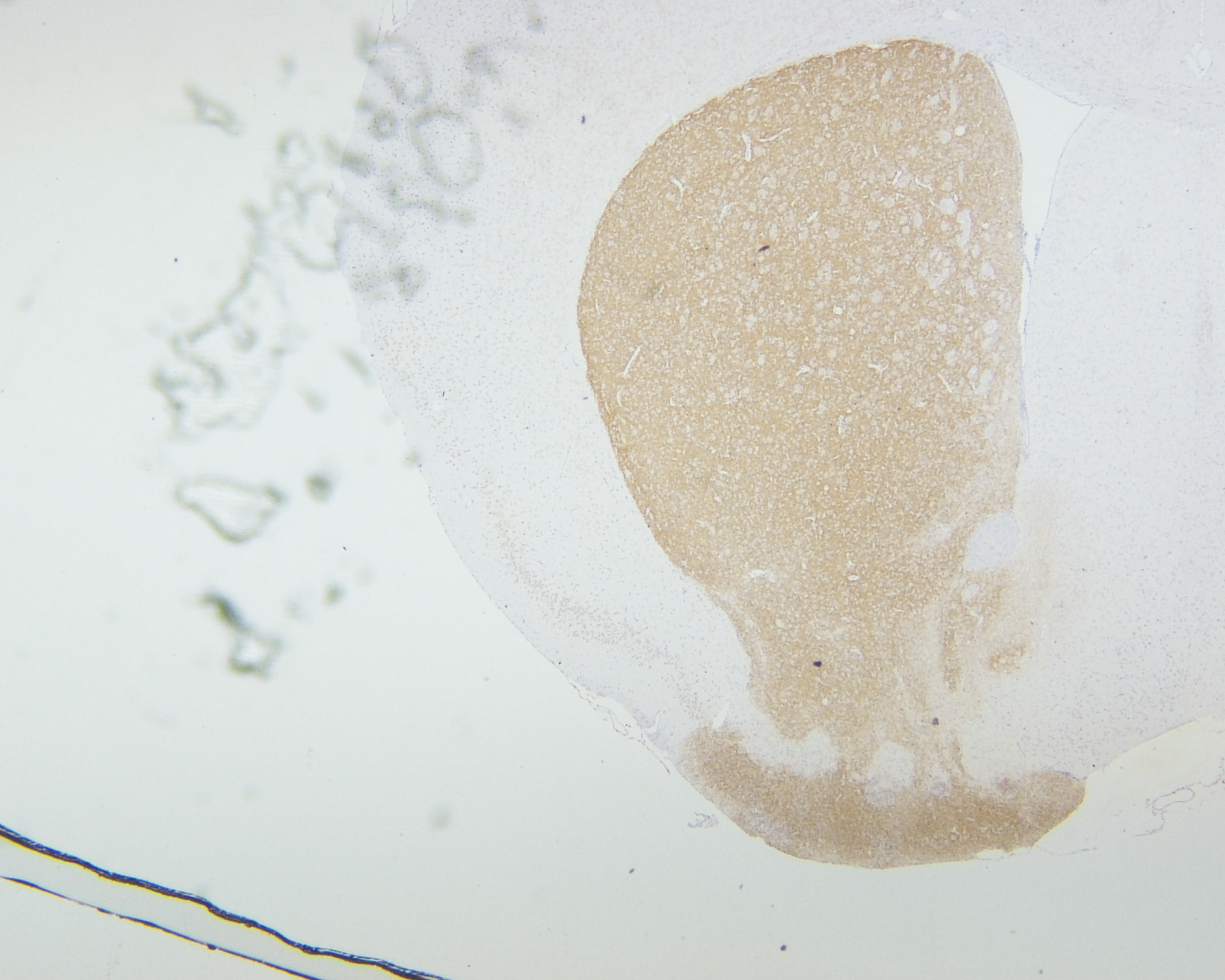

Supplement: Supplementary file 7 — Source data Fig. 4 [file 44318_2025_430_MOESM7_ESM.zip › Figure 4/Figure 4C/Selenot fl fl ;Dat-cre.tif]

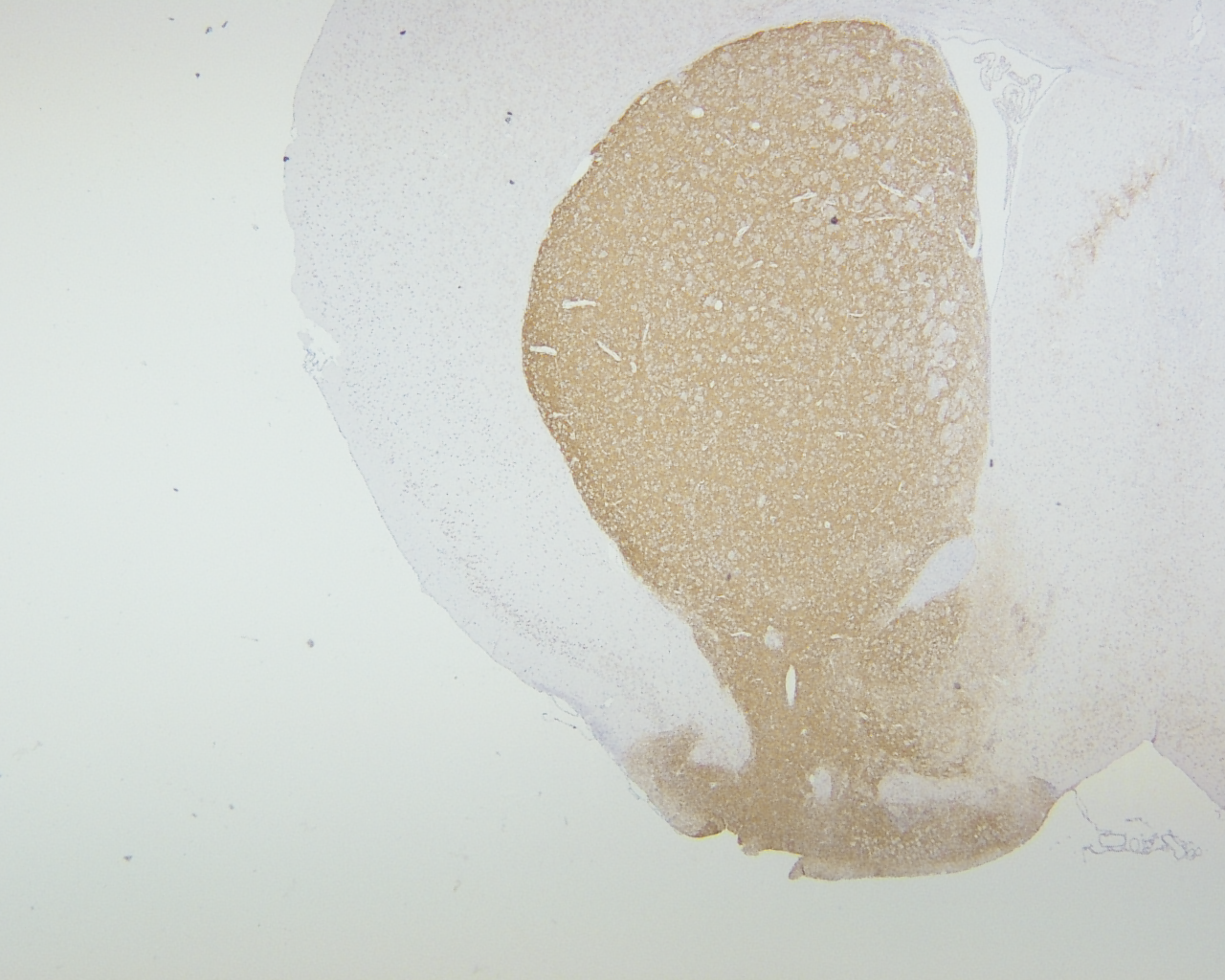

Supplement: Supplementary file 7 — Source data Fig. 4 [file 44318_2025_430_MOESM7_ESM.zip › Figure 4/Figure 4C/Selenot fl fl.tif]

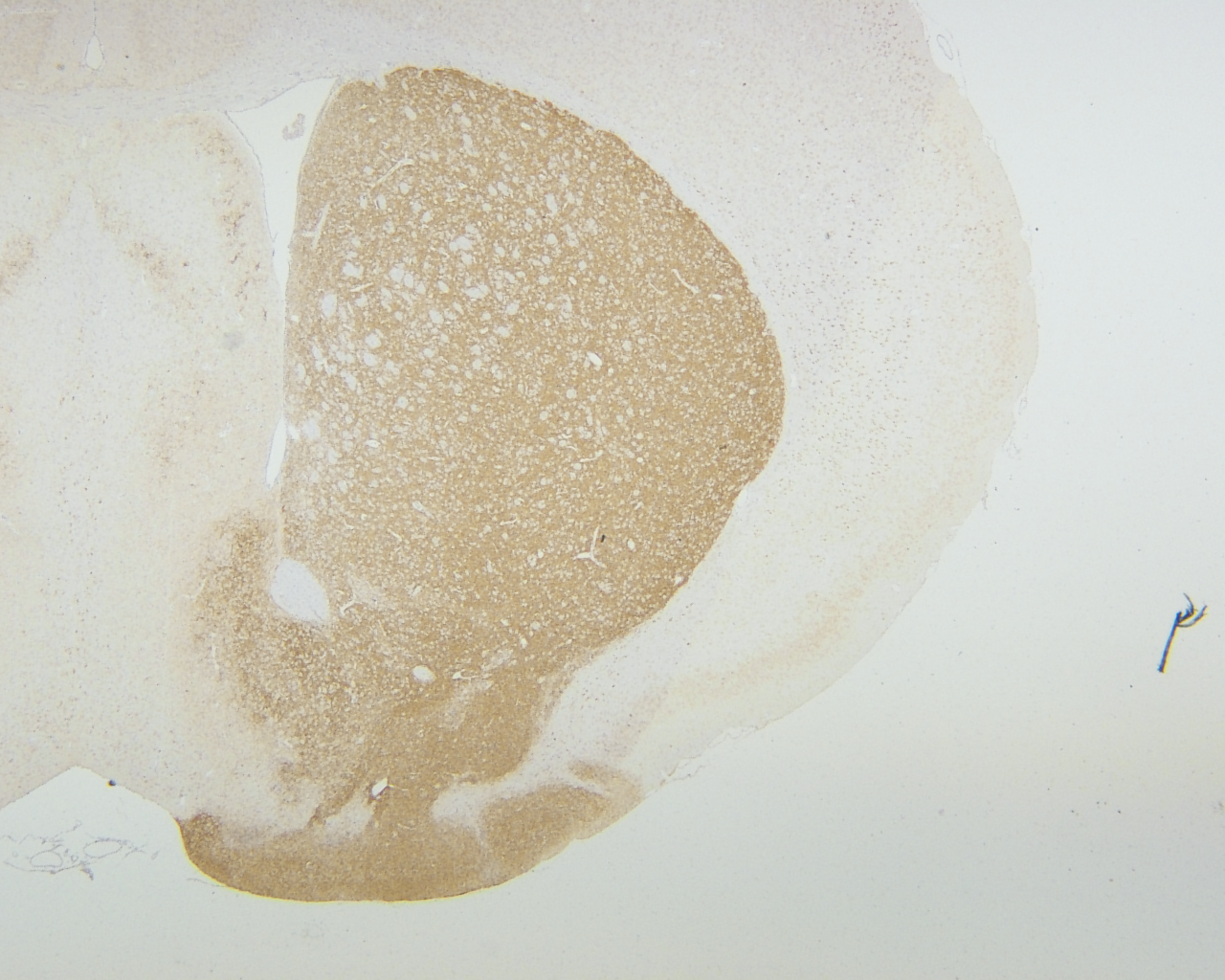

Supplement: Supplementary file 7 — Source data Fig. 4 [file 44318_2025_430_MOESM7_ESM.zip › Figure 4/Figure 4E/Selenot fl fl.tif]

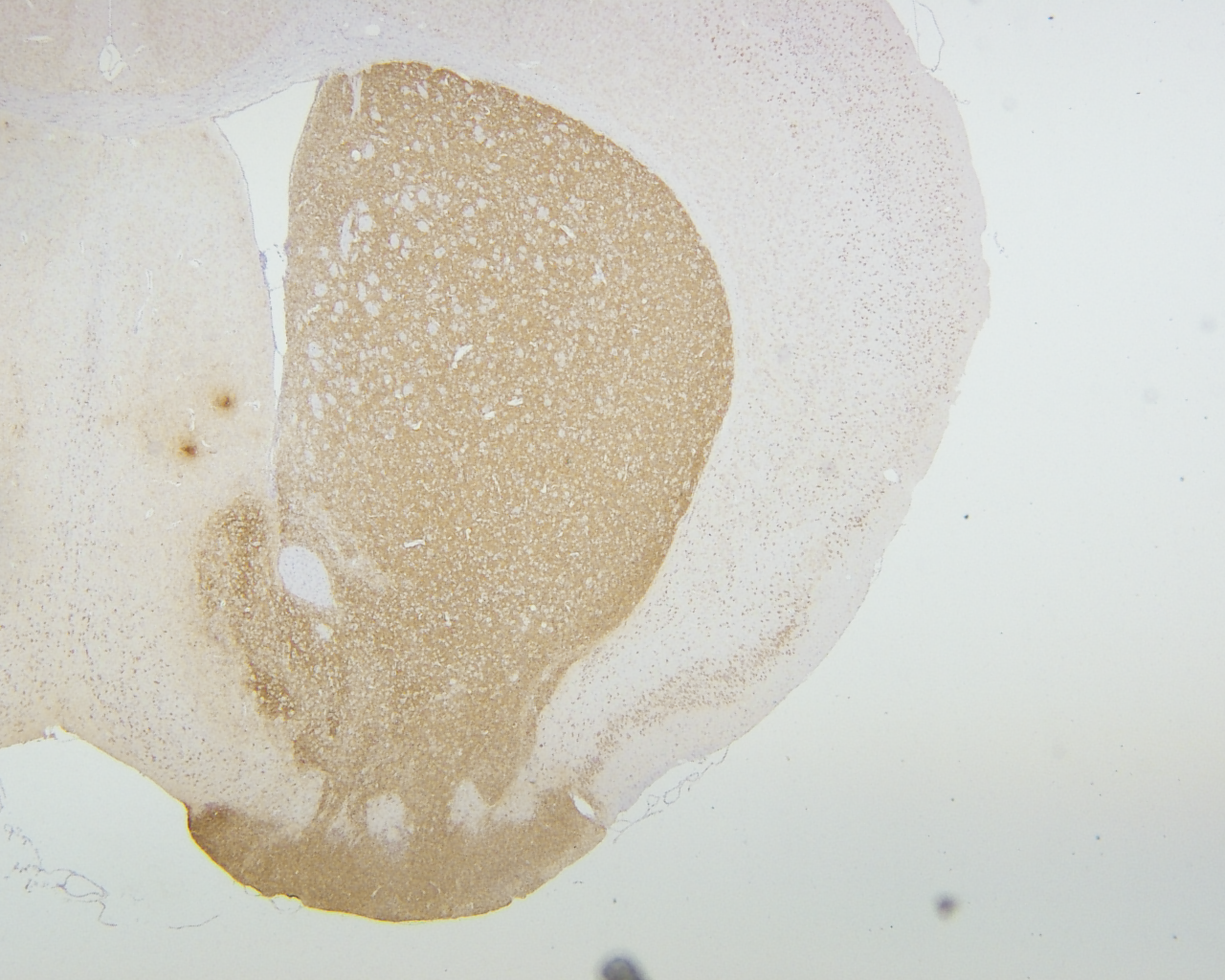

Supplement: Supplementary file 7 — Source data Fig. 4 [file 44318_2025_430_MOESM7_ESM.zip › Figure 4/Figure 4E/Selenot fl fl;Dat-cre.tif]

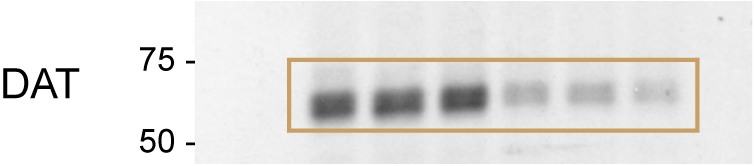

Supplement: Supplementary file 7 — Source data Fig. 4 [file 44318_2025_430_MOESM7_ESM.zip › Figure 4/Figure 4G/SN DAT.tif]

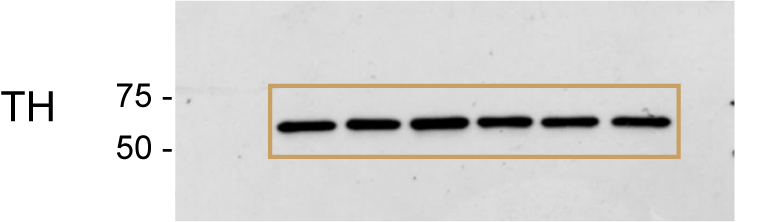

Supplement: Supplementary file 7 — Source data Fig. 4 [file 44318_2025_430_MOESM7_ESM.zip › Figure 4/Figure 4G/SN TH.tif]

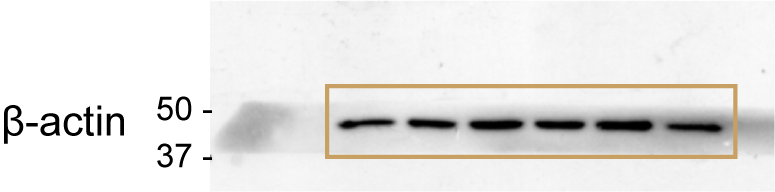

Supplement: Supplementary file 7 — Source data Fig. 4 [file 44318_2025_430_MOESM7_ESM.zip › Figure 4/Figure 4G/SN a┬-actin.tif]

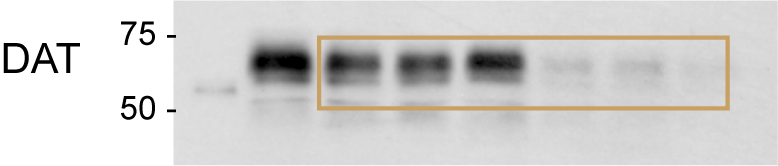

Supplement: Supplementary file 7 — Source data Fig. 4 [file 44318_2025_430_MOESM7_ESM.zip › Figure 4/Figure 4G/VTA DAT.tif]

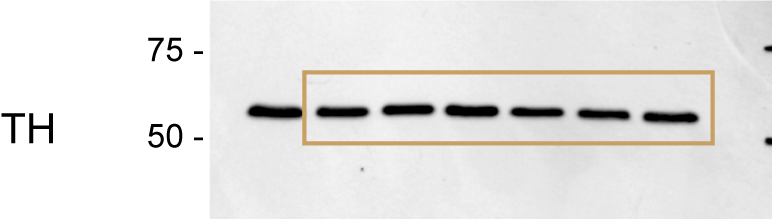

Supplement: Supplementary file 7 — Source data Fig. 4 [file 44318_2025_430_MOESM7_ESM.zip › Figure 4/Figure 4G/VTA TH.tif]

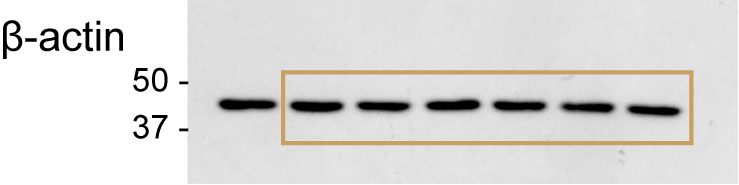

Supplement: Supplementary file 7 — Source data Fig. 4 [file 44318_2025_430_MOESM7_ESM.zip › Figure 4/Figure 4G/VTA a┬-actin.tif]

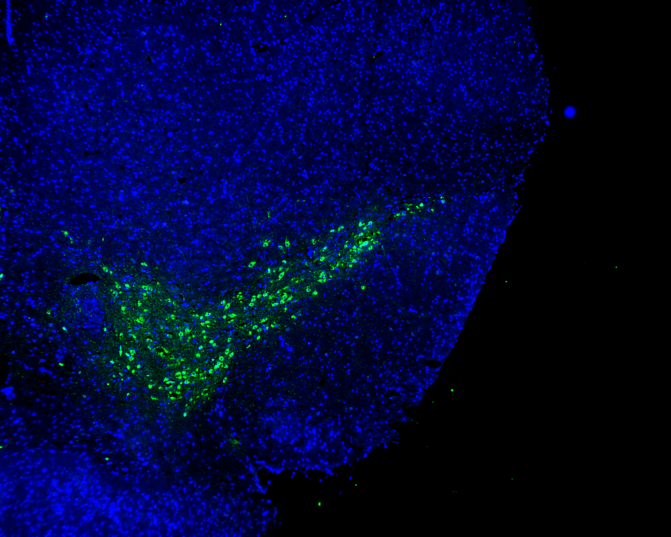

Supplement: Supplementary file 7 — Source data Fig. 4 [file 44318_2025_430_MOESM7_ESM.zip › Figure 4/Figure 4I/selenot DAT-cre.tif]

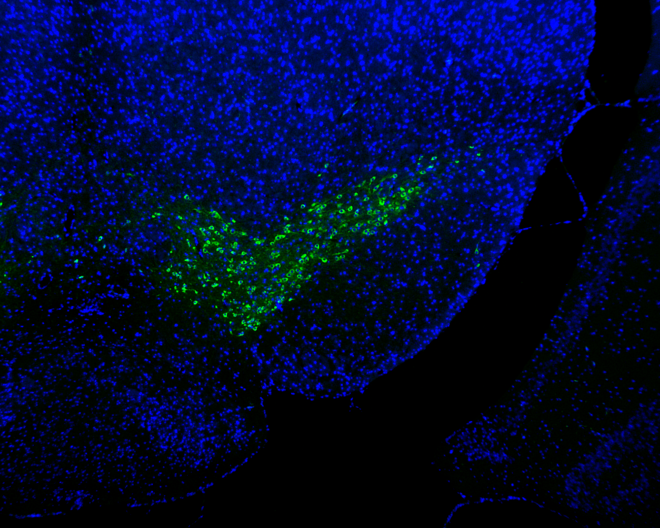

Supplement: Supplementary file 7 — Source data Fig. 4 [file 44318_2025_430_MOESM7_ESM.zip › Figure 4/Figure 4I/selenotflfl.tif]

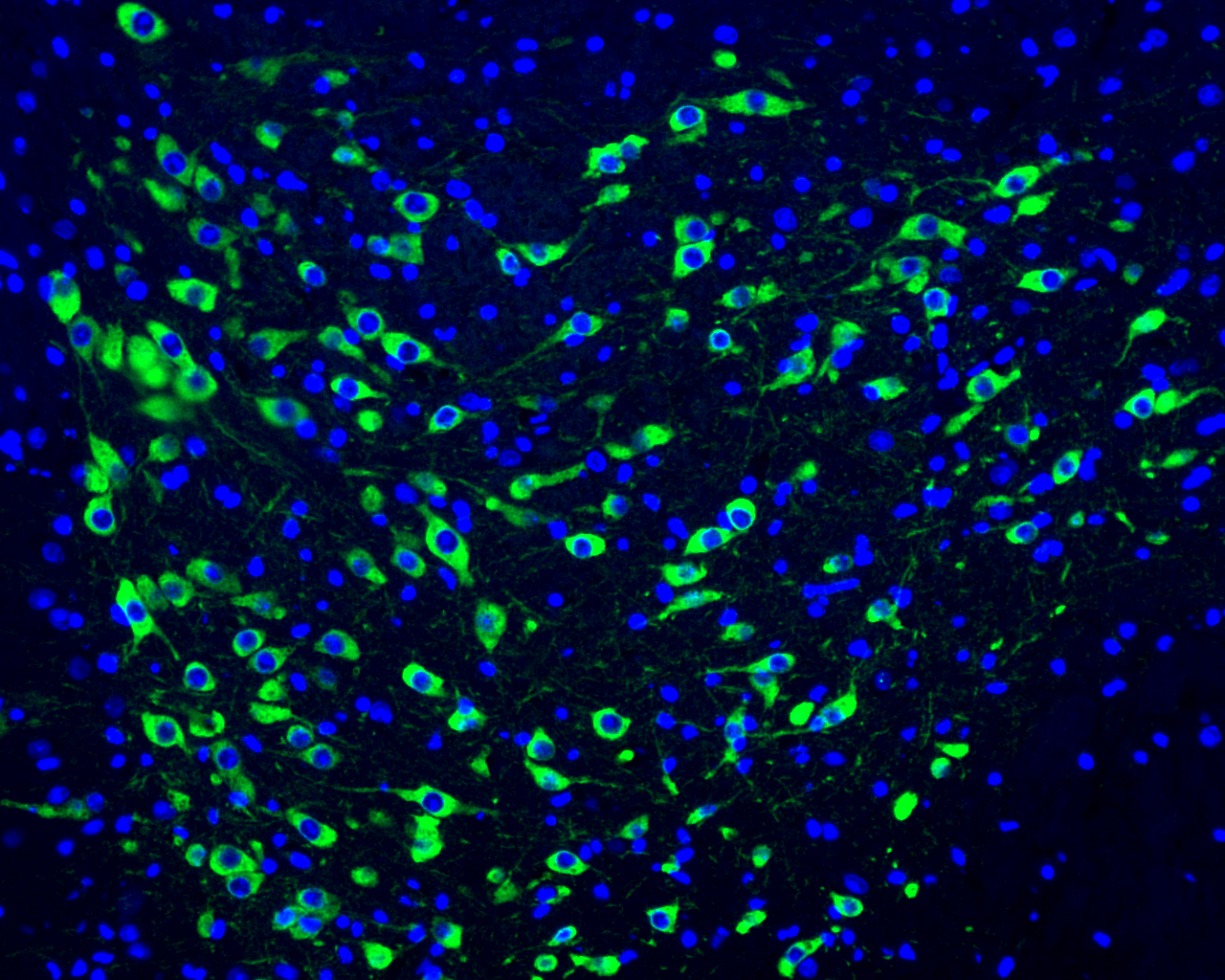

Supplement: Supplementary file 7 — Source data Fig. 4 [file 44318_2025_430_MOESM7_ESM.zip › Figure 4/Figure 4K/selenot flfl.tif]

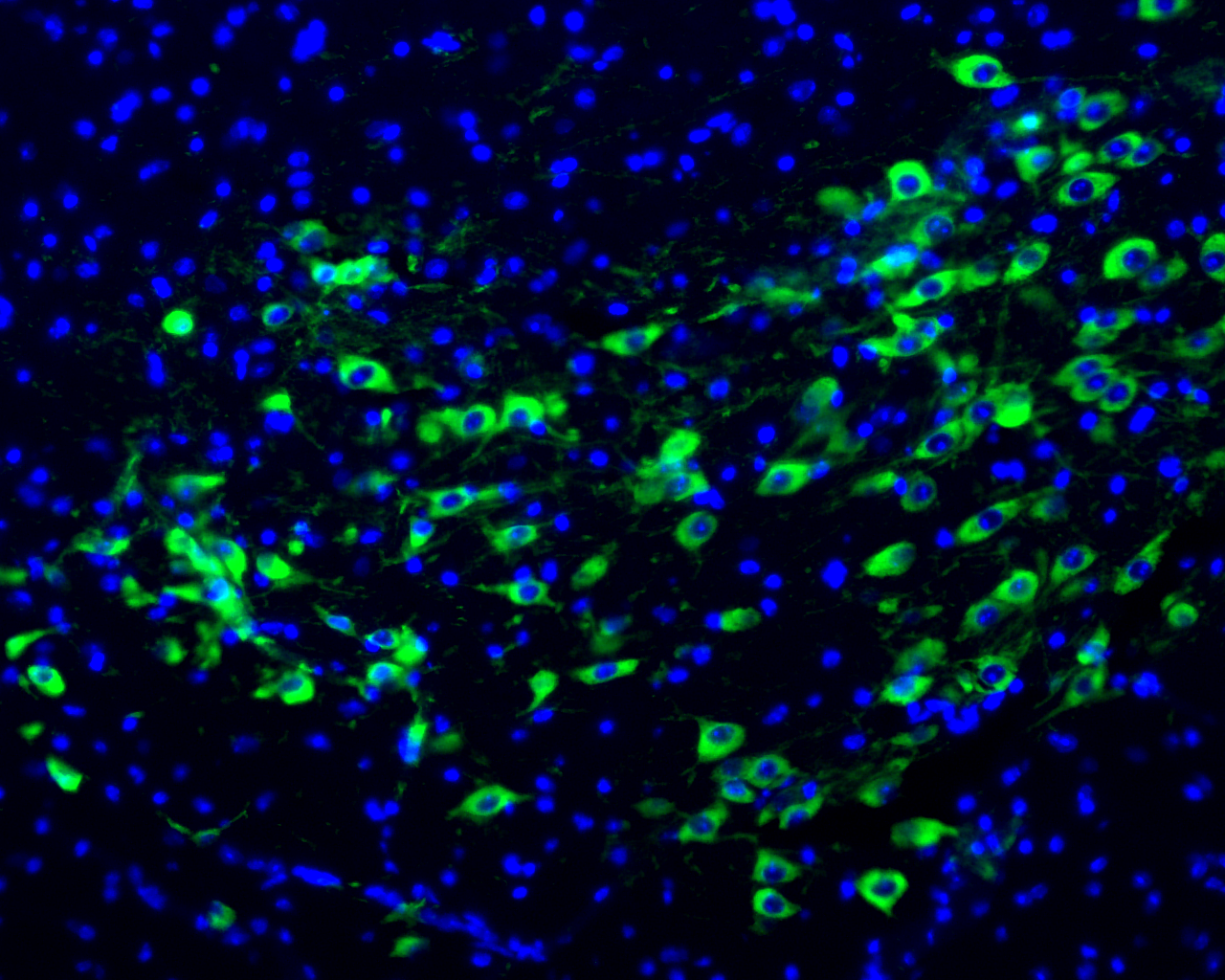

Supplement: Supplementary file 7 — Source data Fig. 4 [file 44318_2025_430_MOESM7_ESM.zip › Figure 4/Figure 4K/selenotflfl Dat-cre.tif]

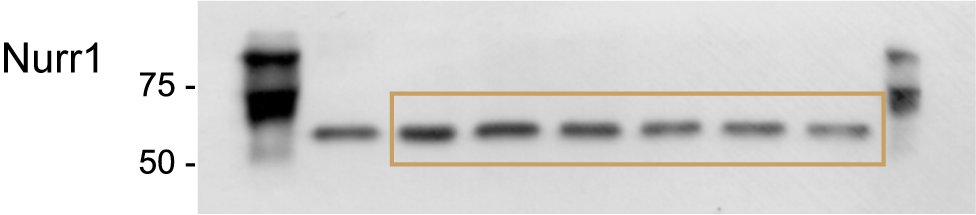

Supplement: Supplementary file 8 — Source data Fig. 5 [file 44318_2025_430_MOESM8_ESM.zip › Figure 5/Figure5G/NURR1.tif]

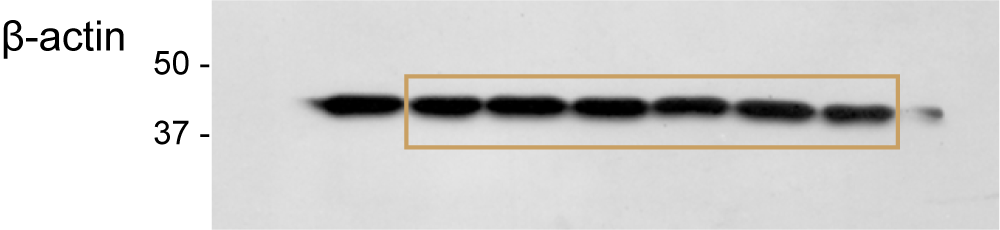

Supplement: Supplementary file 8 — Source data Fig. 5 [file 44318_2025_430_MOESM8_ESM.zip › Figure 5/Figure5G/a┬-actin.tif]

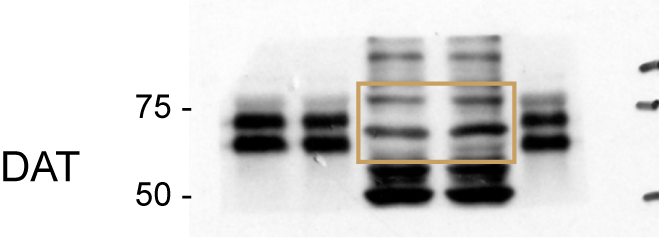

Supplement: Supplementary file 9 — Source data Fig. 6 [file 44318_2025_430_MOESM9_ESM.zip › Figure 6/Figure 6A/pMyc-SELT DAT.tif]

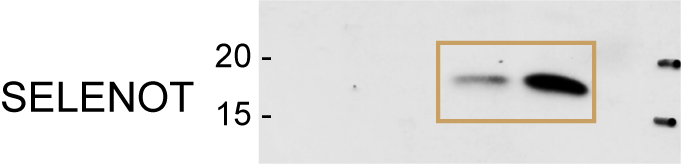

Supplement: Supplementary file 9 — Source data Fig. 6 [file 44318_2025_430_MOESM9_ESM.zip › Figure 6/Figure 6A/pMyc-SELT SELENOT.tif]

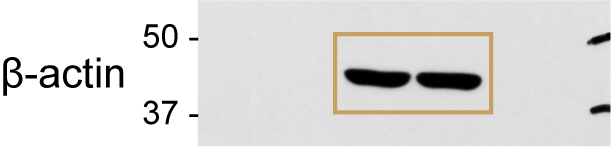

Supplement: Supplementary file 9 — Source data Fig. 6 [file 44318_2025_430_MOESM9_ESM.zip › Figure 6/Figure 6A/pMyc-SELT a┬-actin.tif]

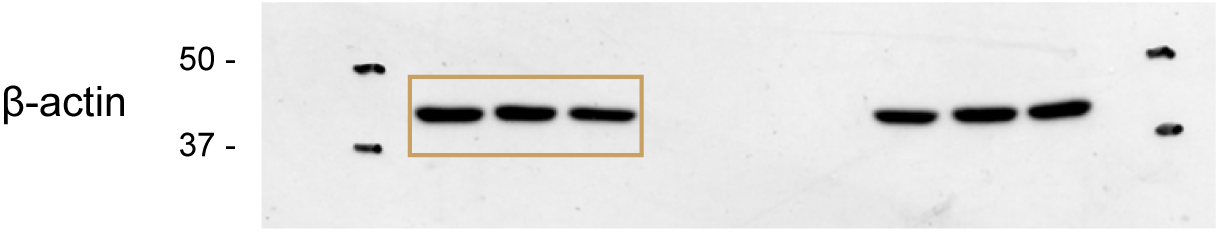

Supplement: Supplementary file 9 — Source data Fig. 6 [file 44318_2025_430_MOESM9_ESM.zip › Figure 6/Figure 6A/siSELT a┬-actin.tif]

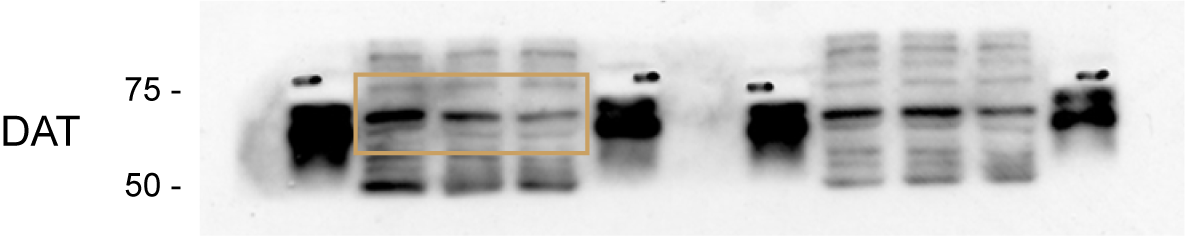

Supplement: Supplementary file 9 — Source data Fig. 6 [file 44318_2025_430_MOESM9_ESM.zip › Figure 6/Figure 6A/siSELT DAT.tif]

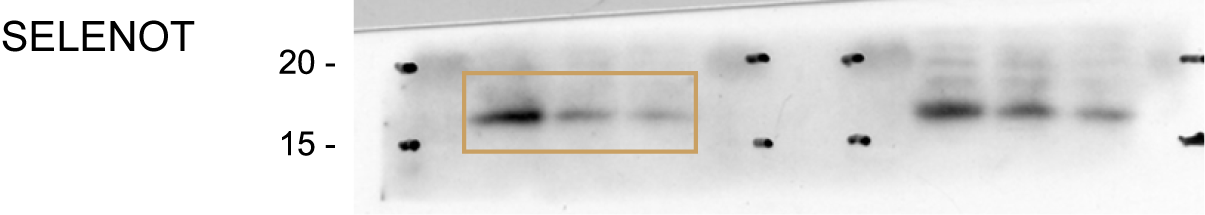

Supplement: Supplementary file 9 — Source data Fig. 6 [file 44318_2025_430_MOESM9_ESM.zip › Figure 6/Figure 6A/siSELT SELENOT.tif]

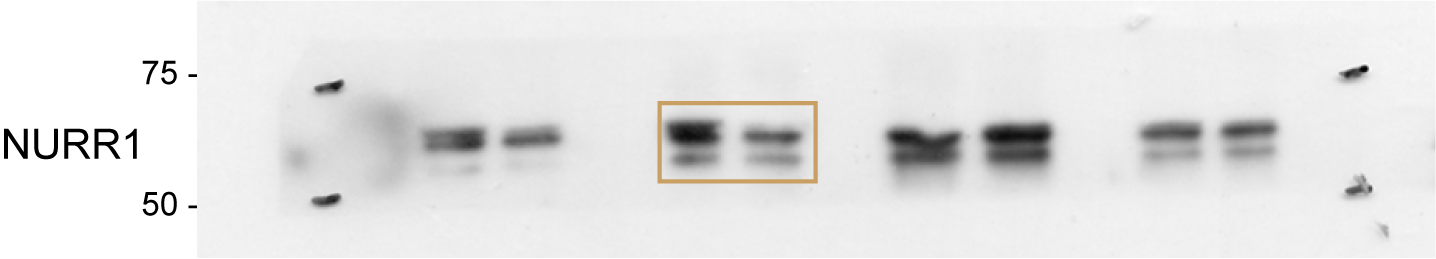

Supplement: Supplementary file 9 — Source data Fig. 6 [file 44318_2025_430_MOESM9_ESM.zip › Figure 6/Figure6E/NURR1.tif]

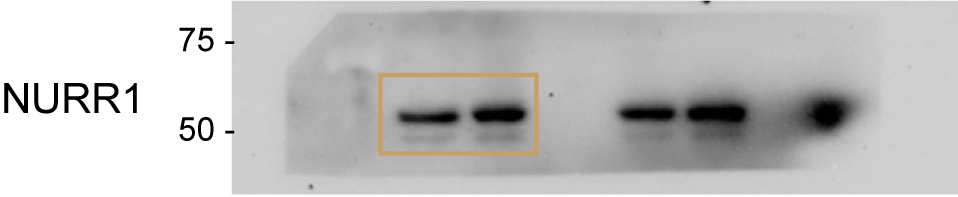

Supplement: Supplementary file 9 — Source data Fig. 6 [file 44318_2025_430_MOESM9_ESM.zip › Figure 6/Figure6E/pMyc-SELT NURR1.tif]

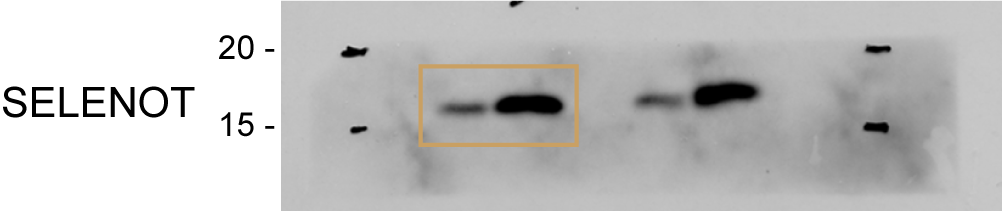

Supplement: Supplementary file 9 — Source data Fig. 6 [file 44318_2025_430_MOESM9_ESM.zip › Figure 6/Figure6E/pMyc-SELT SELENOT.tif]

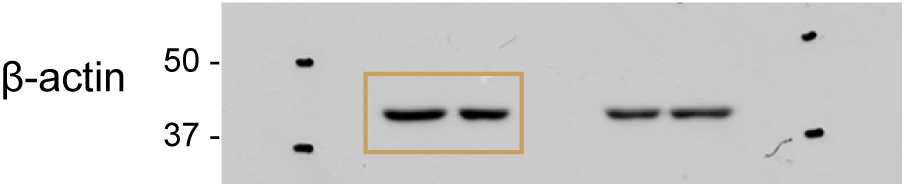

Supplement: Supplementary file 9 — Source data Fig. 6 [file 44318_2025_430_MOESM9_ESM.zip › Figure 6/Figure6E/pMyc-SELT a┬-actin.tif]

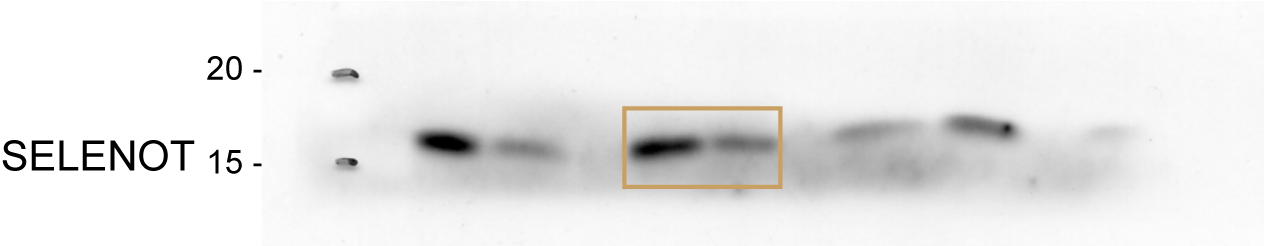

Supplement: Supplementary file 9 — Source data Fig. 6 [file 44318_2025_430_MOESM9_ESM.zip › Figure 6/Figure6E/SELENOT.tif]

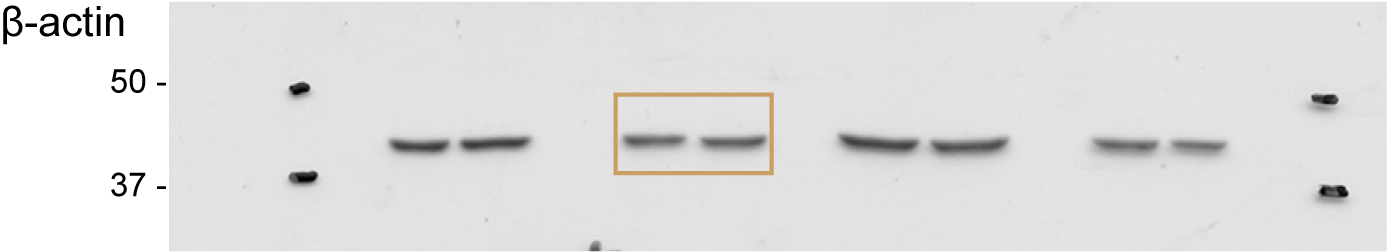

Supplement: Supplementary file 9 — Source data Fig. 6 [file 44318_2025_430_MOESM9_ESM.zip › Figure 6/Figure6E/a┬-actin.tif]

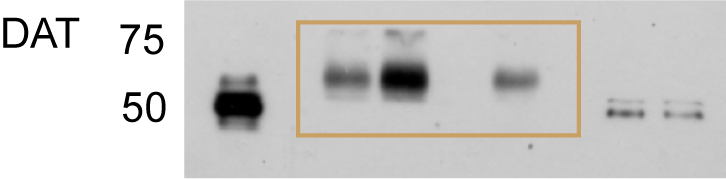

Supplement: Supplementary file 9 — Source data Fig. 6 [file 44318_2025_430_MOESM9_ESM.zip › Figure 6/Figure6I/DAT.tif]

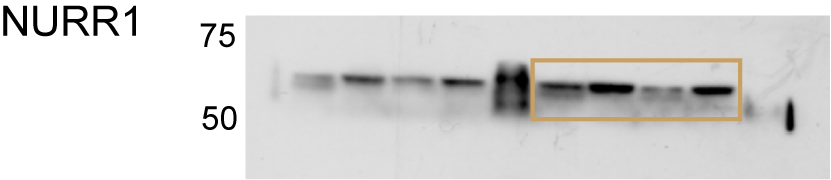

Supplement: Supplementary file 9 — Source data Fig. 6 [file 44318_2025_430_MOESM9_ESM.zip › Figure 6/Figure6I/NURR1.tif]

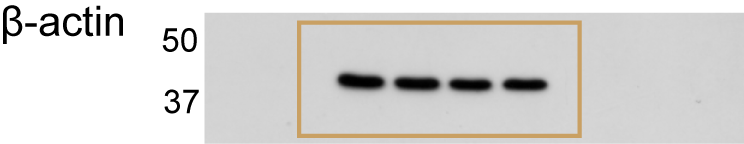

Supplement: Supplementary file 9 — Source data Fig. 6 [file 44318_2025_430_MOESM9_ESM.zip › Figure 6/Figure6I/a┬-actin.tif]

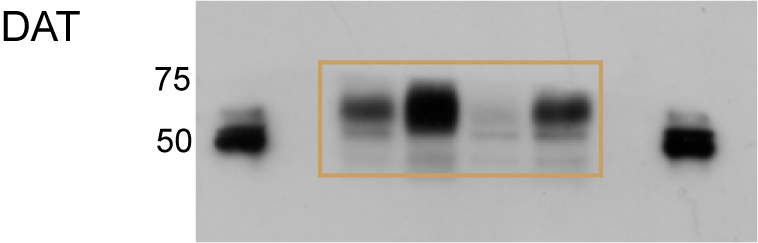

Supplement: Supplementary file 9 — Source data Fig. 6 [file 44318_2025_430_MOESM9_ESM.zip › Figure 6/Figure6K/DAT.tif]

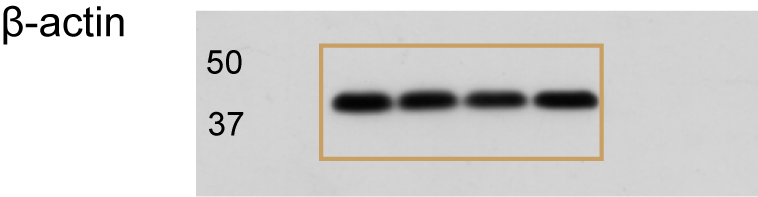

Supplement: Supplementary file 9 — Source data Fig. 6 [file 44318_2025_430_MOESM9_ESM.zip › Figure 6/Figure6K/a┬-actin.tif]

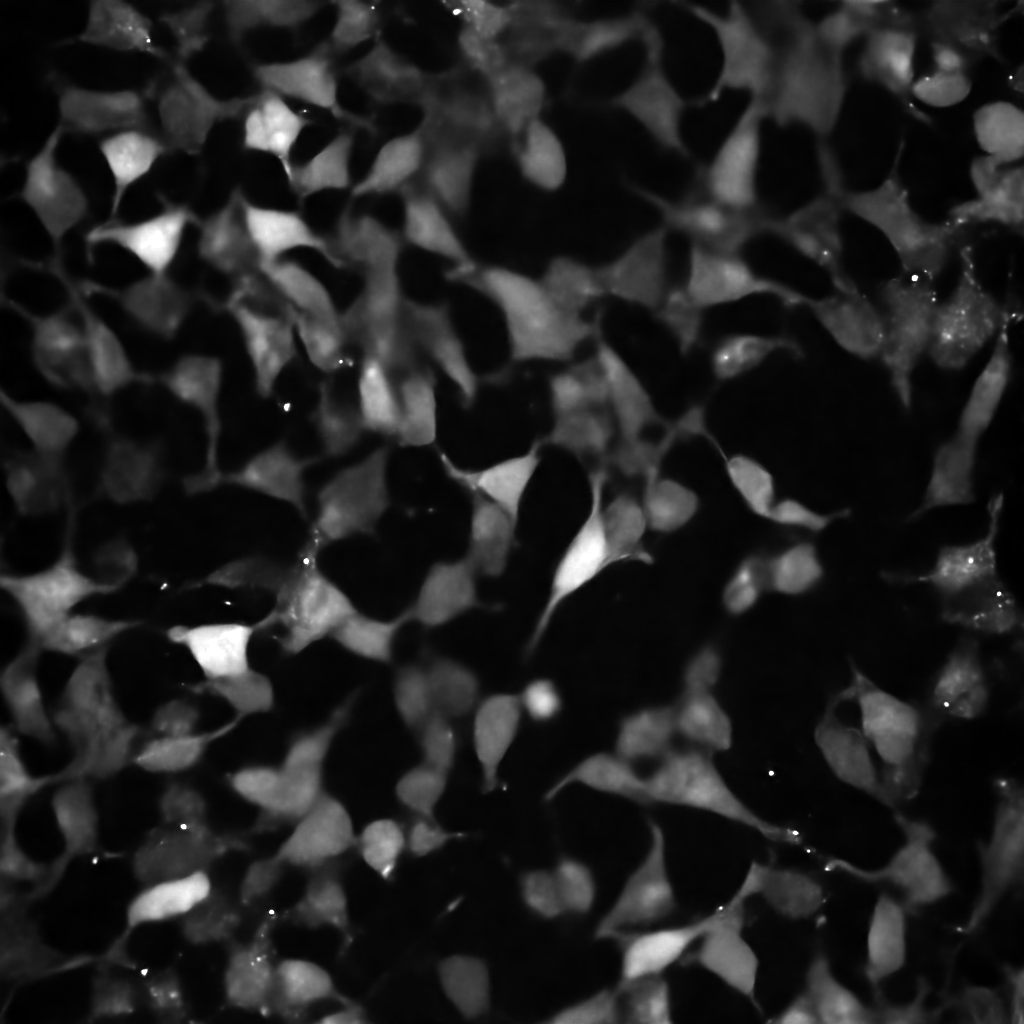

Supplement: Supplementary file 10 — Source data Fig. 7 [file 44318_2025_430_MOESM10_ESM.zip › Figure 7/Figure7A/NC-CPA_T0.tif]

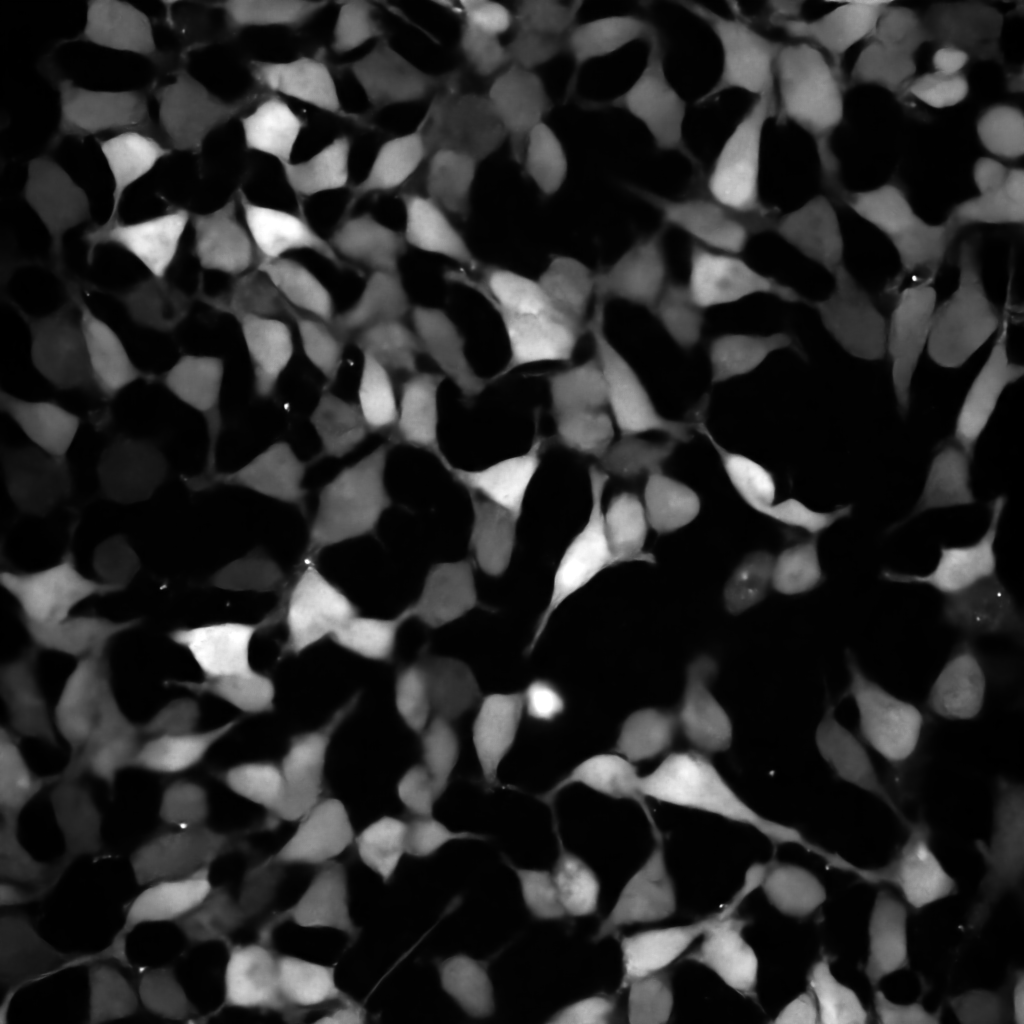

Supplement: Supplementary file 10 — Source data Fig. 7 [file 44318_2025_430_MOESM10_ESM.zip › Figure 7/Figure7A/NC-CPA_T100.tif]

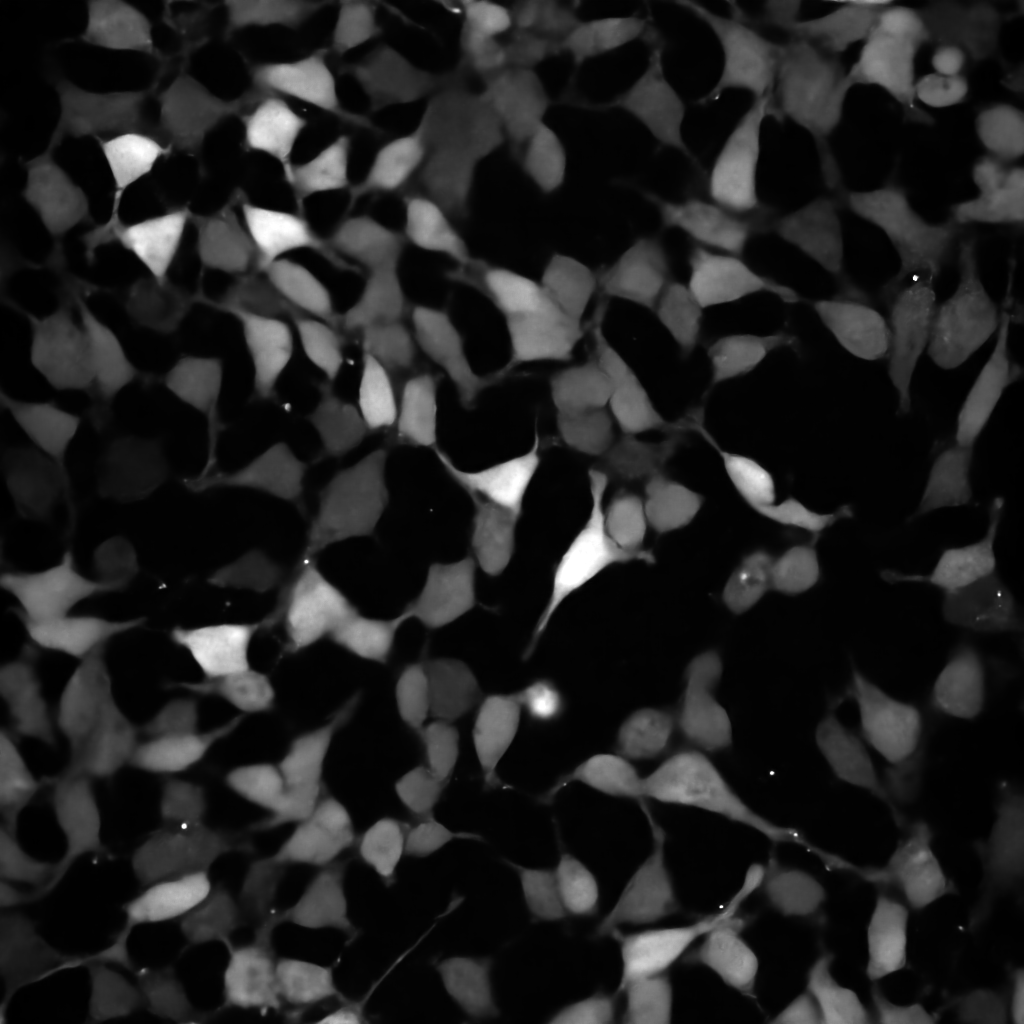

Supplement: Supplementary file 10 — Source data Fig. 7 [file 44318_2025_430_MOESM10_ESM.zip › Figure 7/Figure7A/NC-CPA_T50.tif]

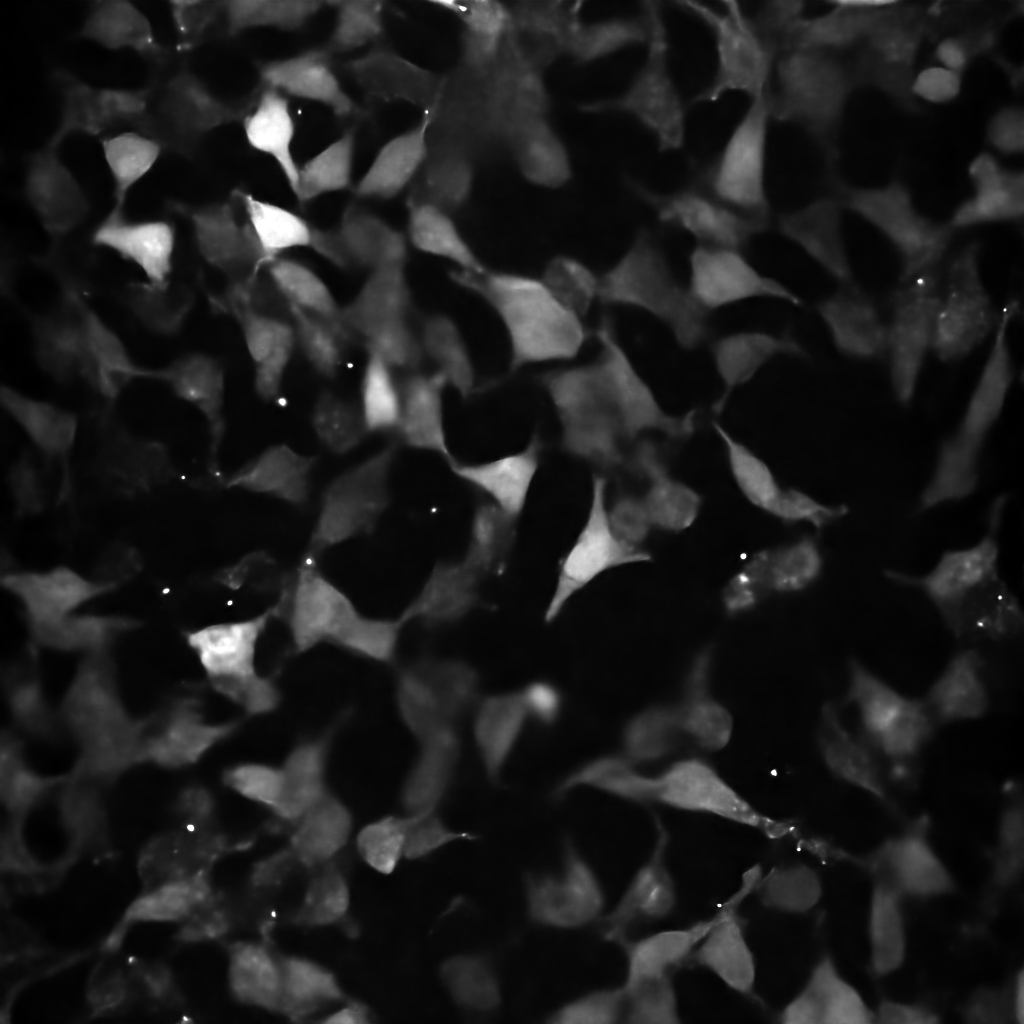

Supplement: Supplementary file 10 — Source data Fig. 7 [file 44318_2025_430_MOESM10_ESM.zip › Figure 7/Figure7A/NC-CPA_T500.tif]

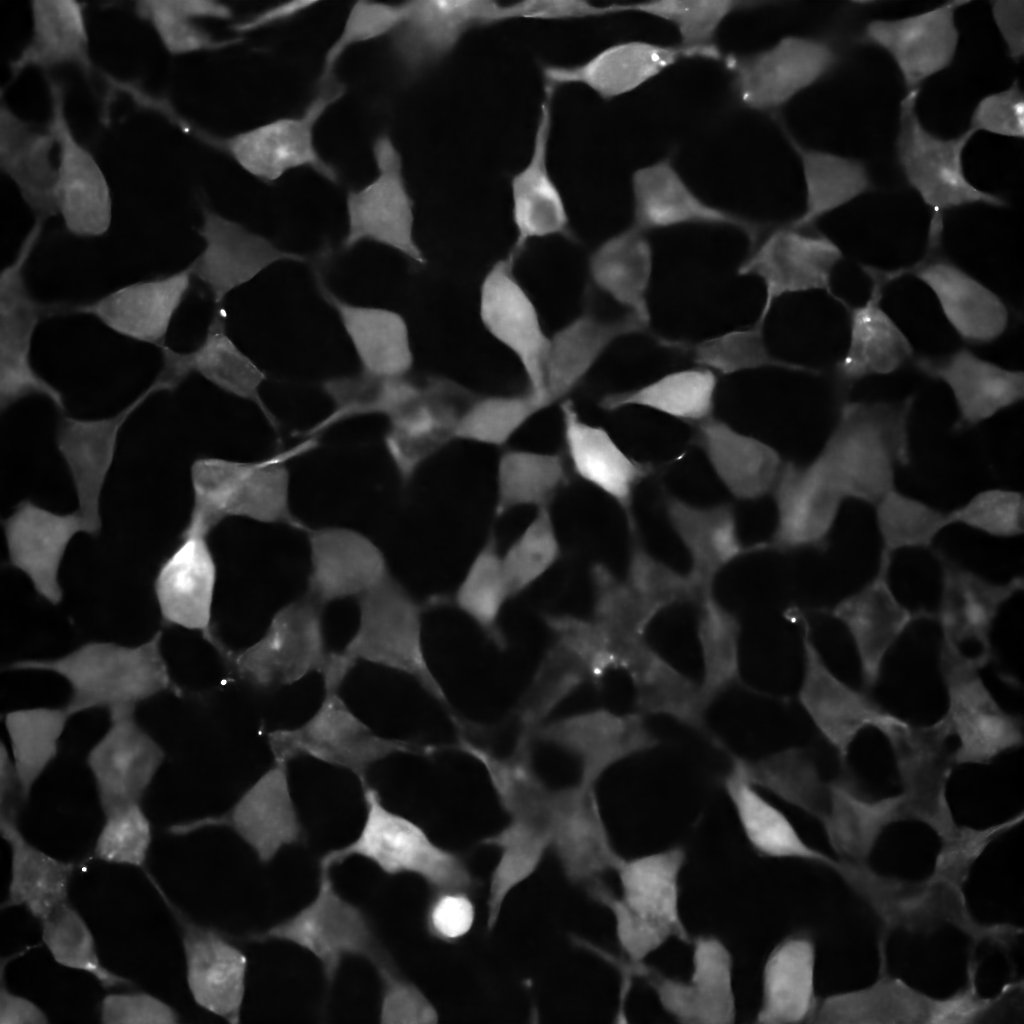

Supplement: Supplementary file 10 — Source data Fig. 7 [file 44318_2025_430_MOESM10_ESM.zip › Figure 7/Figure7A/siSELET-CPA_T0.tif]

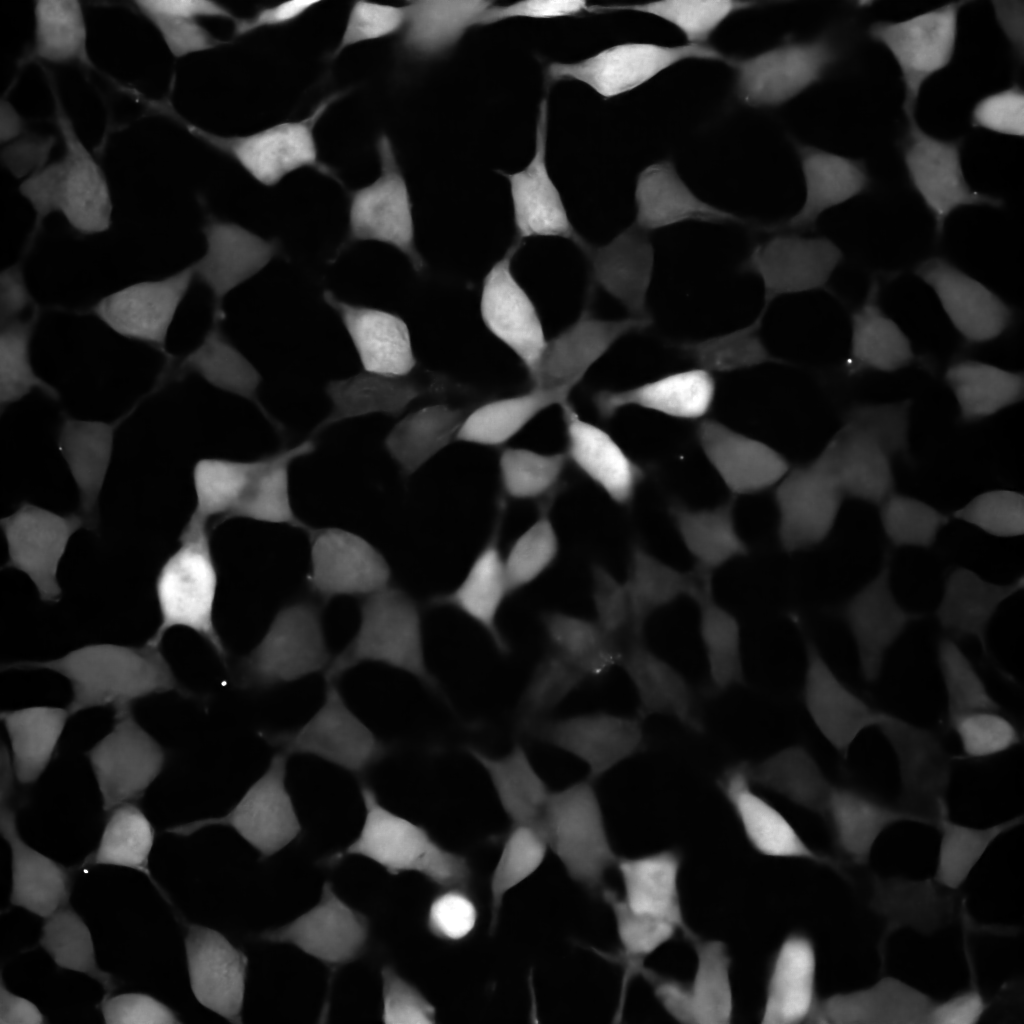

Supplement: Supplementary file 10 — Source data Fig. 7 [file 44318_2025_430_MOESM10_ESM.zip › Figure 7/Figure7A/siSELET-CPA_T100.tif]

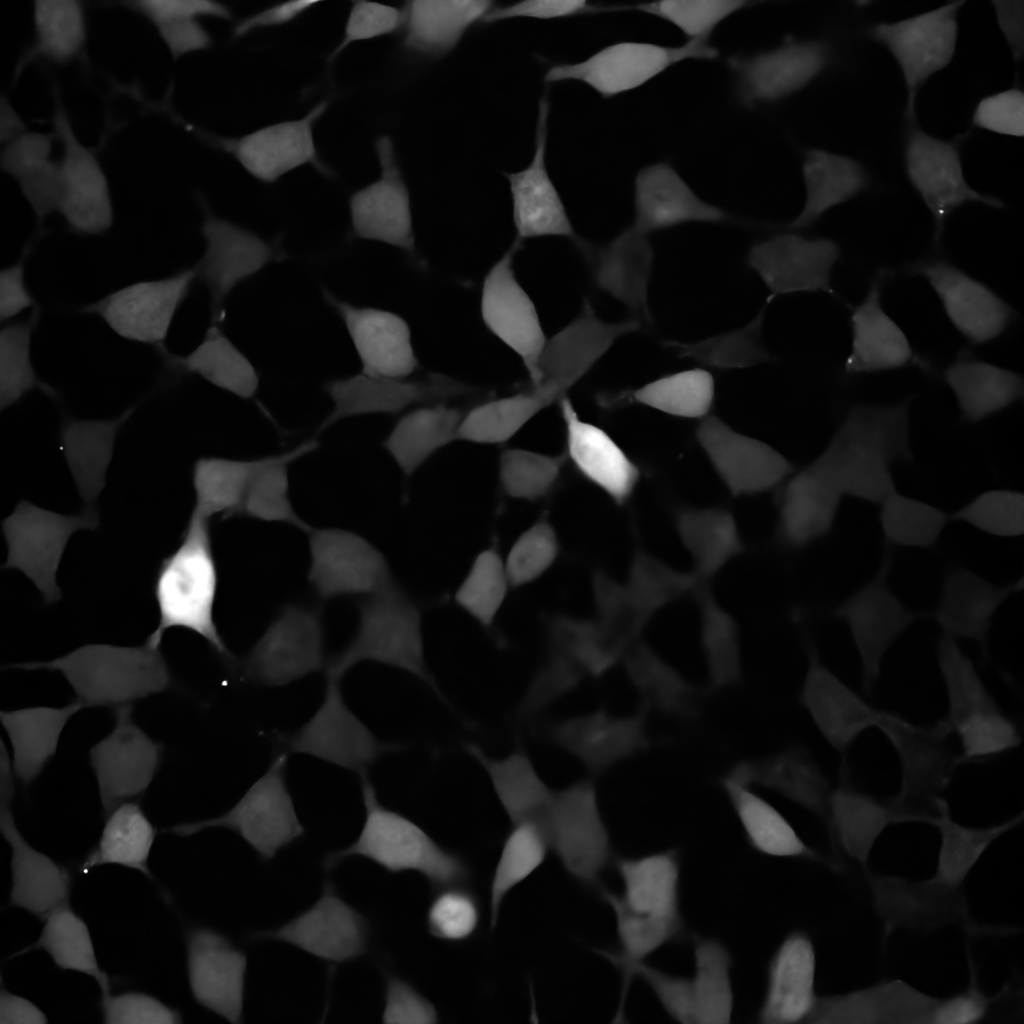

Supplement: Supplementary file 10 — Source data Fig. 7 [file 44318_2025_430_MOESM10_ESM.zip › Figure 7/Figure7A/siSELET-CPA_T50.tif]

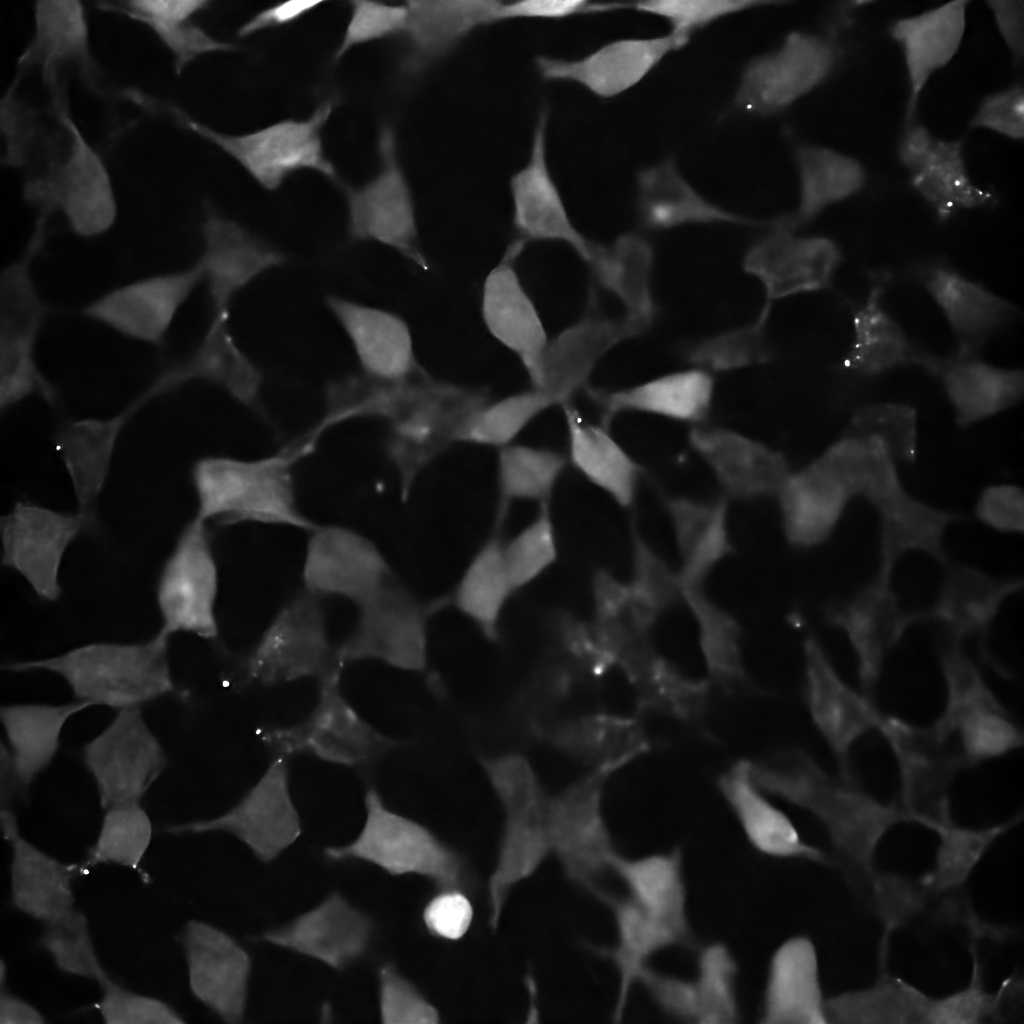

Supplement: Supplementary file 10 — Source data Fig. 7 [file 44318_2025_430_MOESM10_ESM.zip › Figure 7/Figure7A/siSELET-CPA_T500.tif]

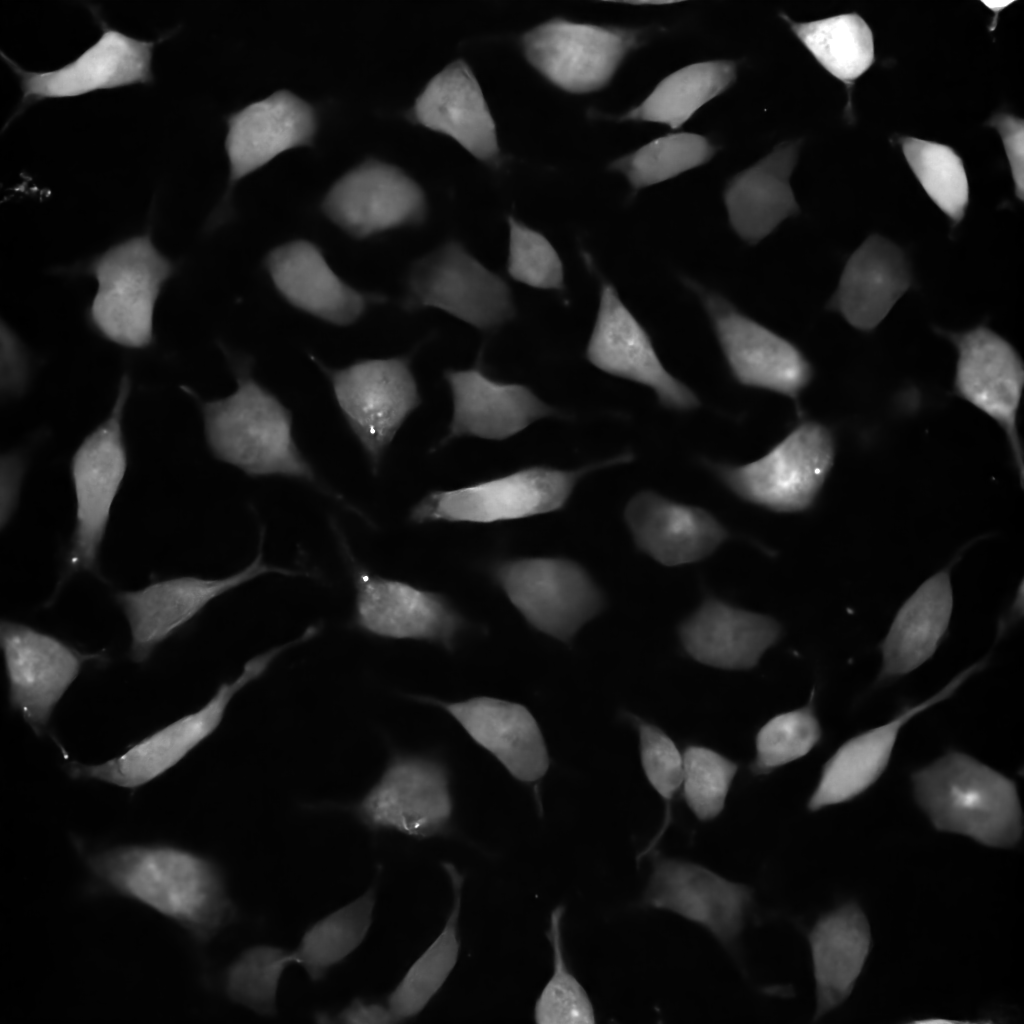

Supplement: Supplementary file 10 — Source data Fig. 7 [file 44318_2025_430_MOESM10_ESM.zip › Figure 7/Figure7F/MYC-SELT _T0.tif]

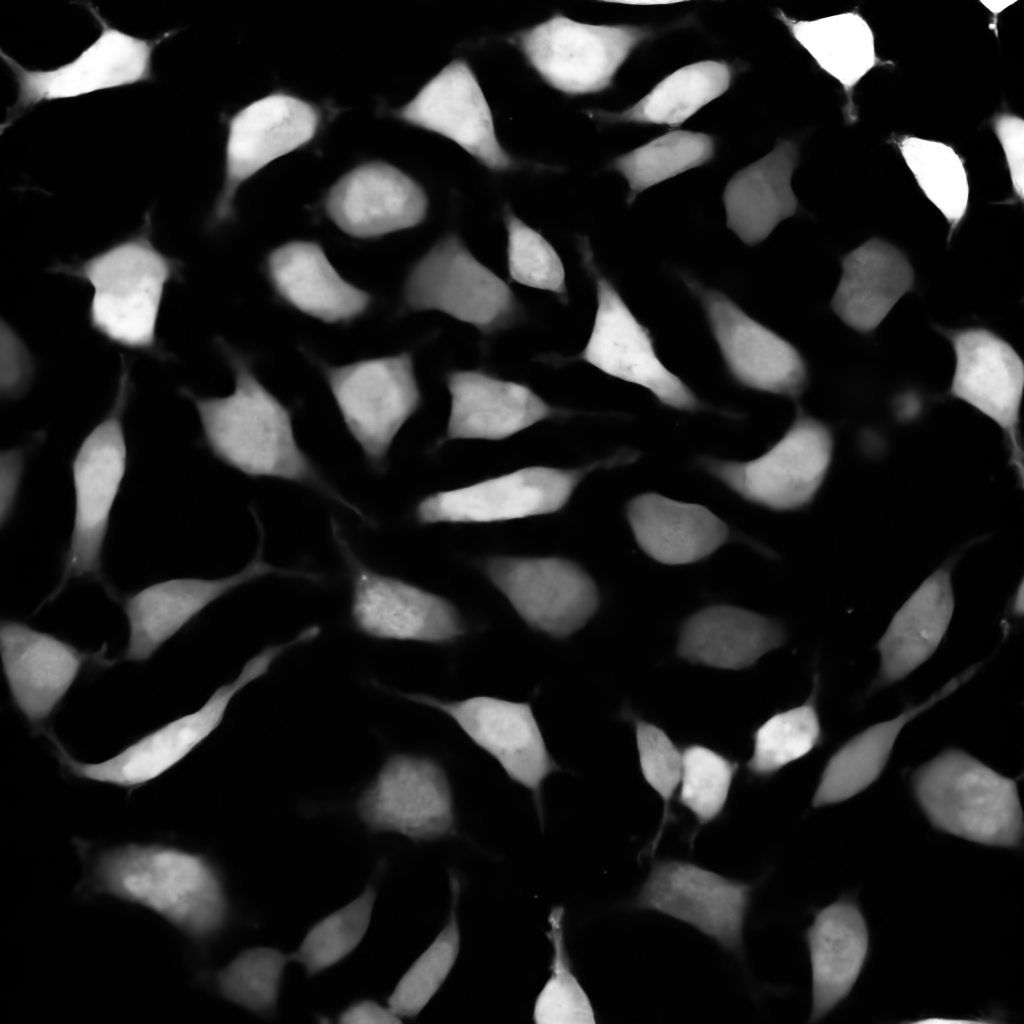

Supplement: Supplementary file 10 — Source data Fig. 7 [file 44318_2025_430_MOESM10_ESM.zip › Figure 7/Figure7F/MYC-SELT _T100.tif]

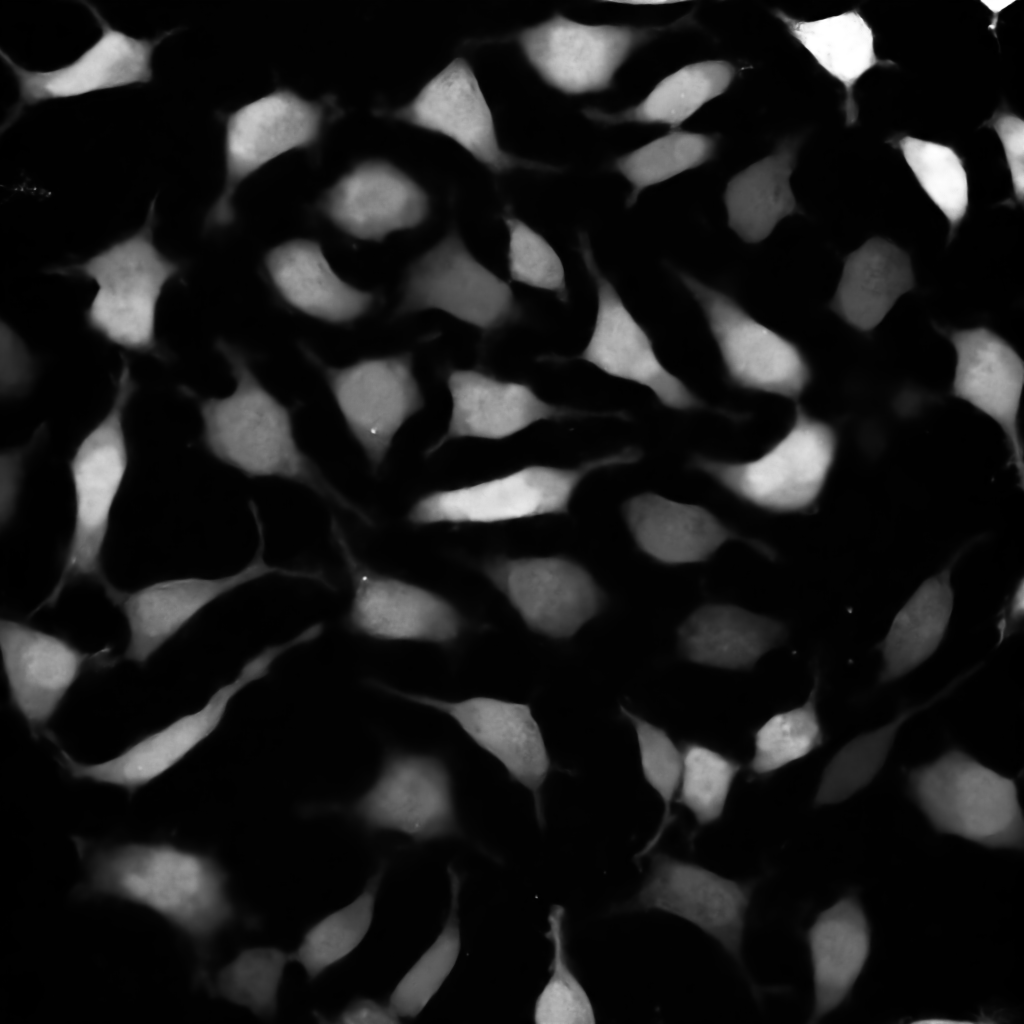

Supplement: Supplementary file 10 — Source data Fig. 7 [file 44318_2025_430_MOESM10_ESM.zip › Figure 7/Figure7F/MYC-SELT _T50.tif]

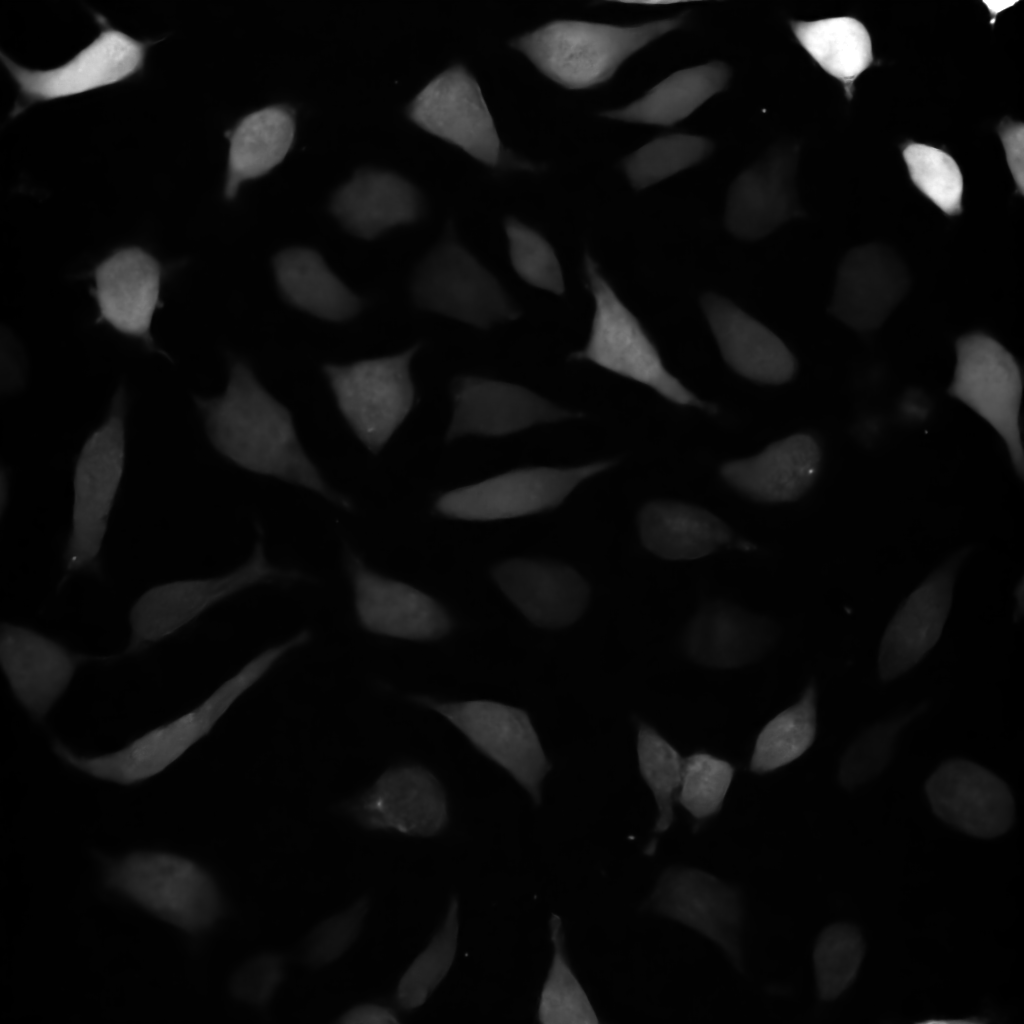

Supplement: Supplementary file 10 — Source data Fig. 7 [file 44318_2025_430_MOESM10_ESM.zip › Figure 7/Figure7F/MYC-SELT _T500.tif]

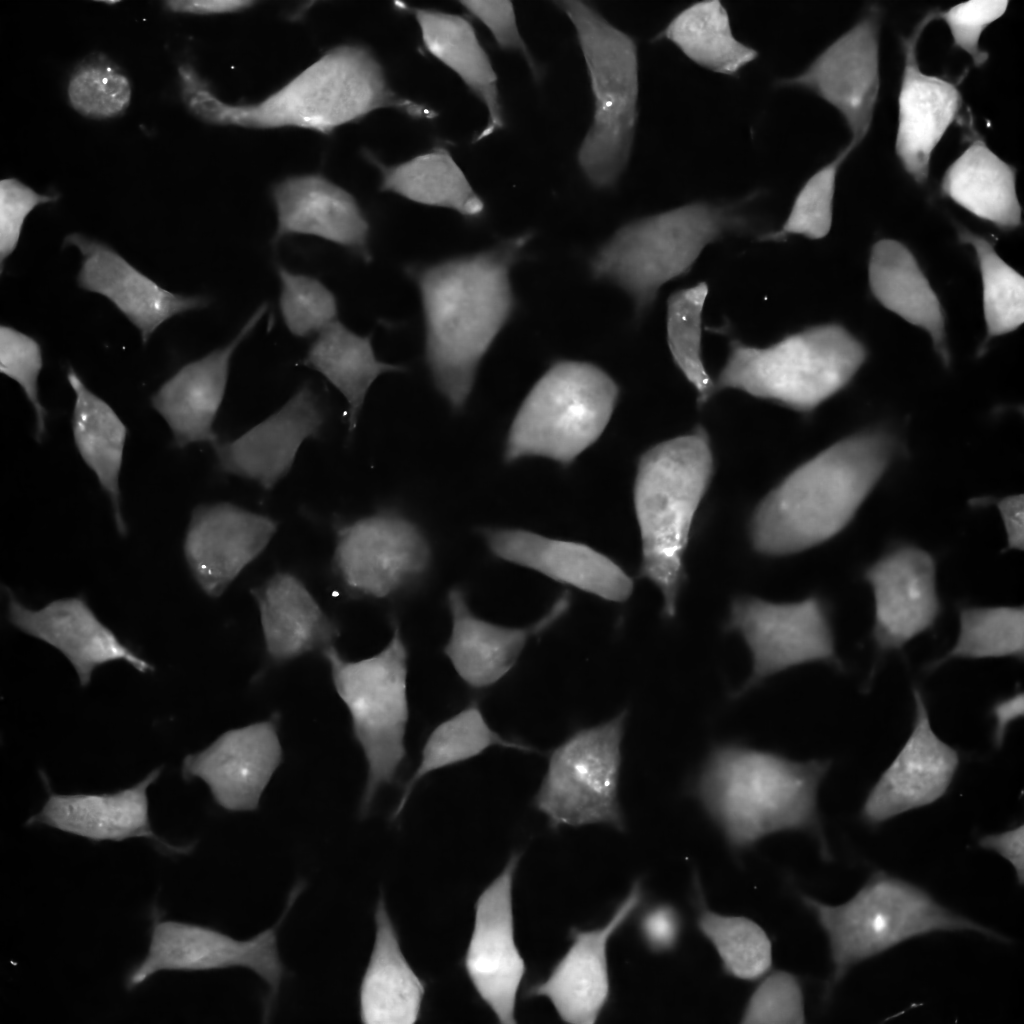

Supplement: Supplementary file 10 — Source data Fig. 7 [file 44318_2025_430_MOESM10_ESM.zip › Figure 7/Figure7F/MYC_T0.tif]

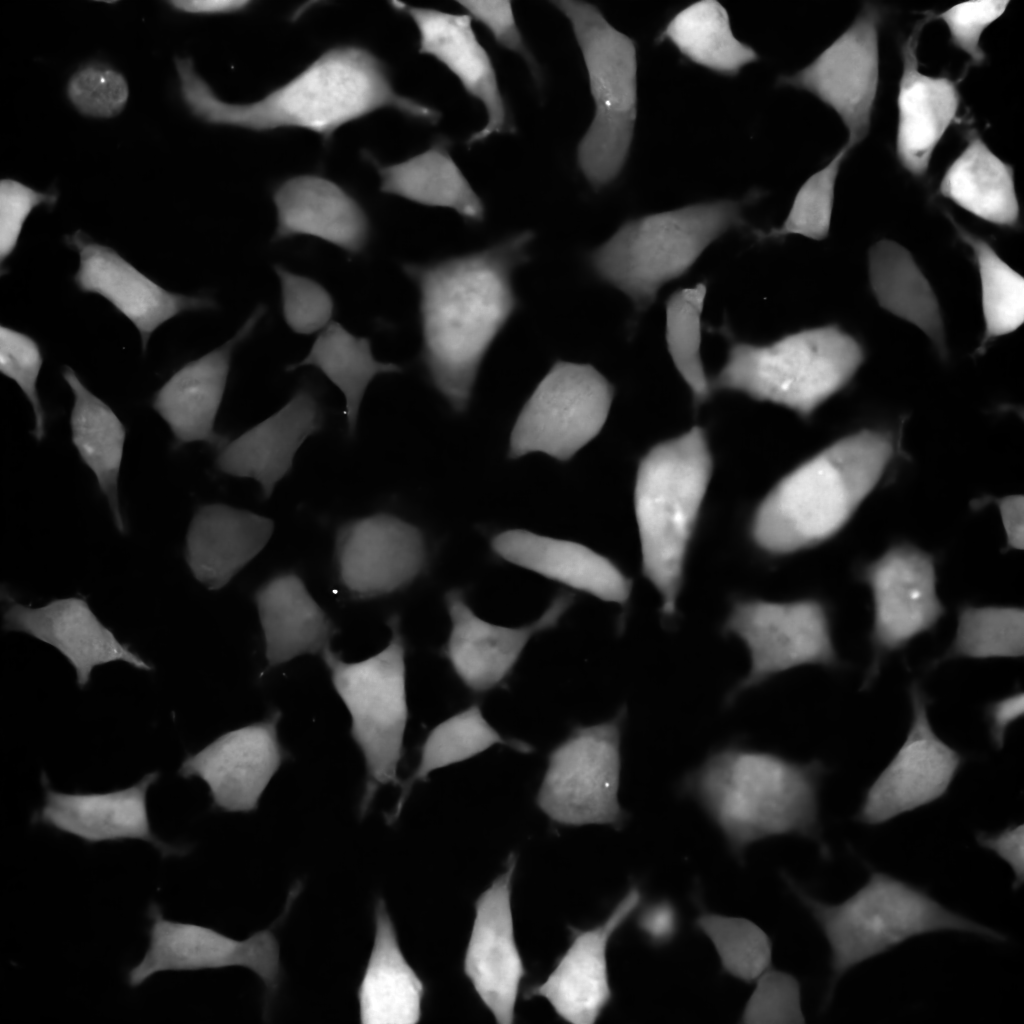

Supplement: Supplementary file 10 — Source data Fig. 7 [file 44318_2025_430_MOESM10_ESM.zip › Figure 7/Figure7F/MYC_T100.tif]

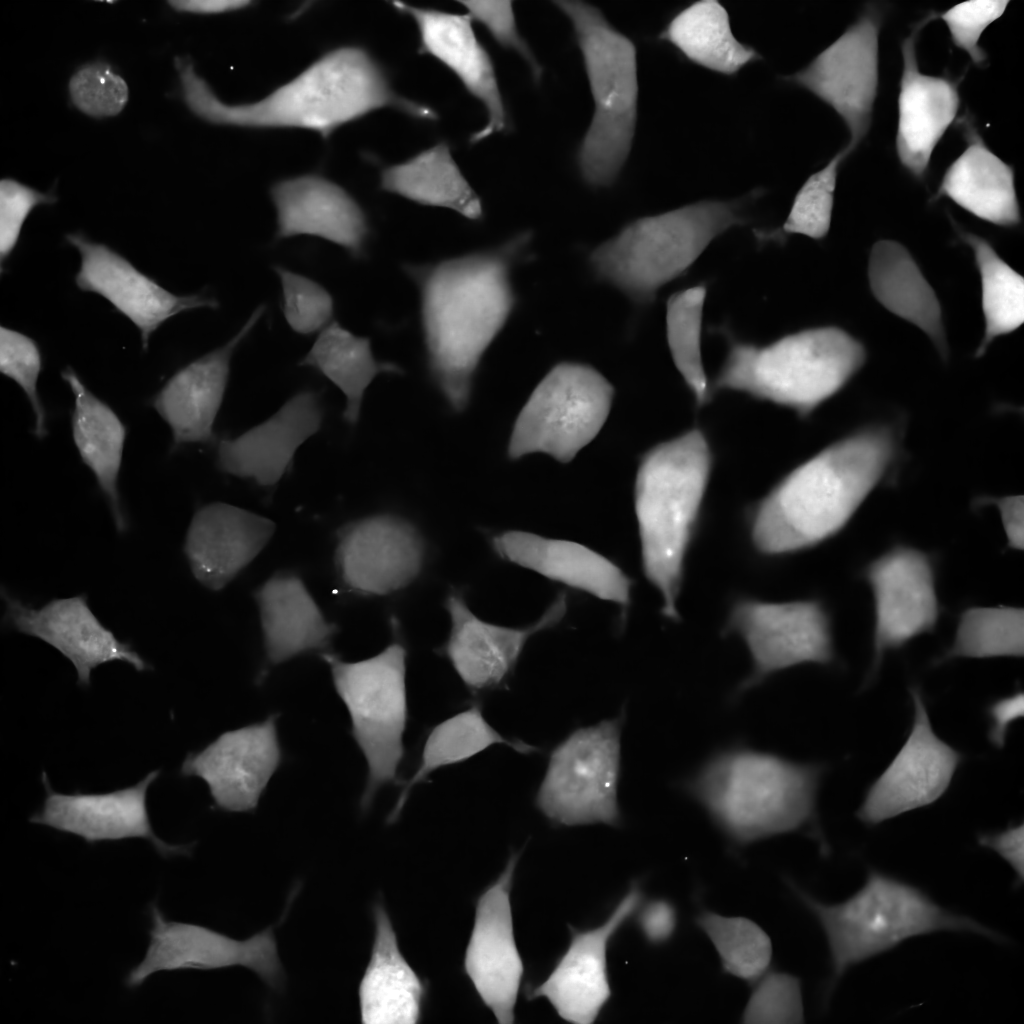

Supplement: Supplementary file 10 — Source data Fig. 7 [file 44318_2025_430_MOESM10_ESM.zip › Figure 7/Figure7F/MYC_T50.tif]

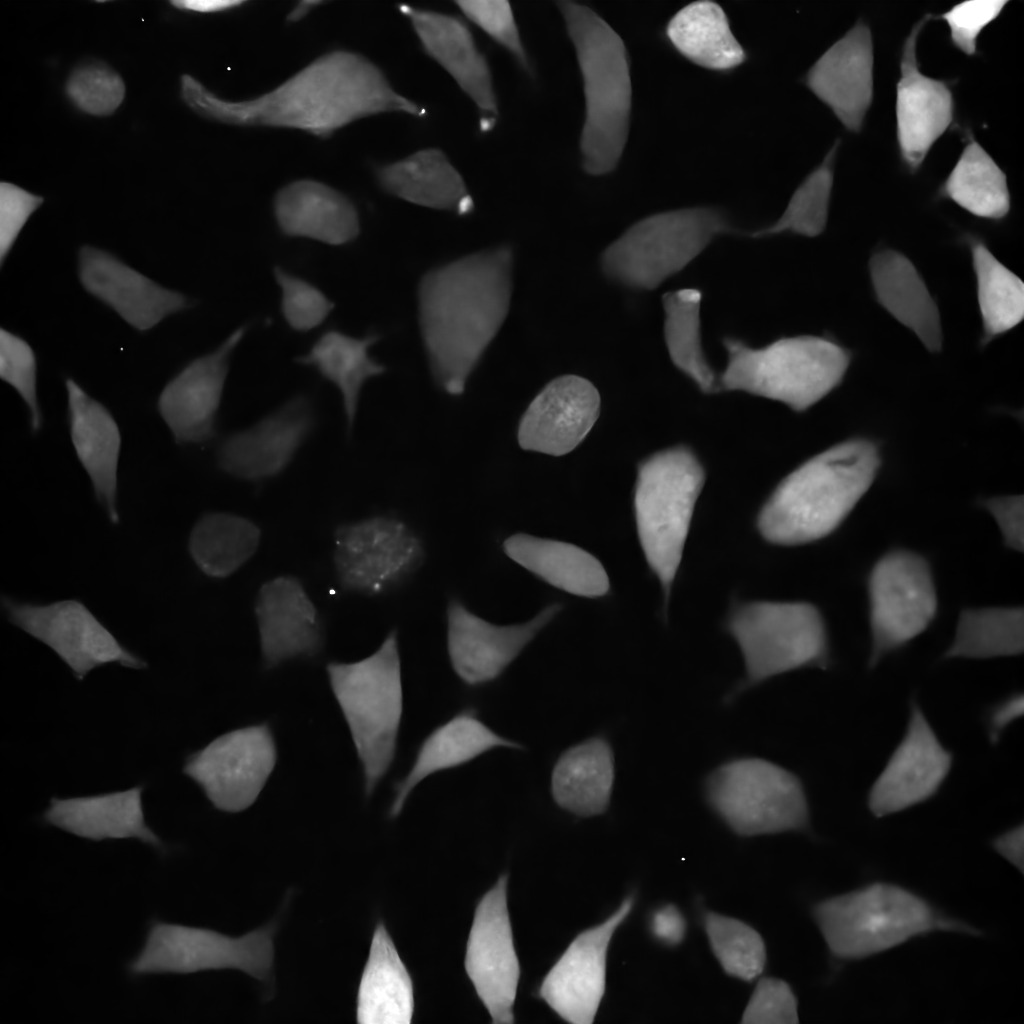

Supplement: Supplementary file 10 — Source data Fig. 7 [file 44318_2025_430_MOESM10_ESM.zip › Figure 7/Figure7F/MYC_T500.tif]

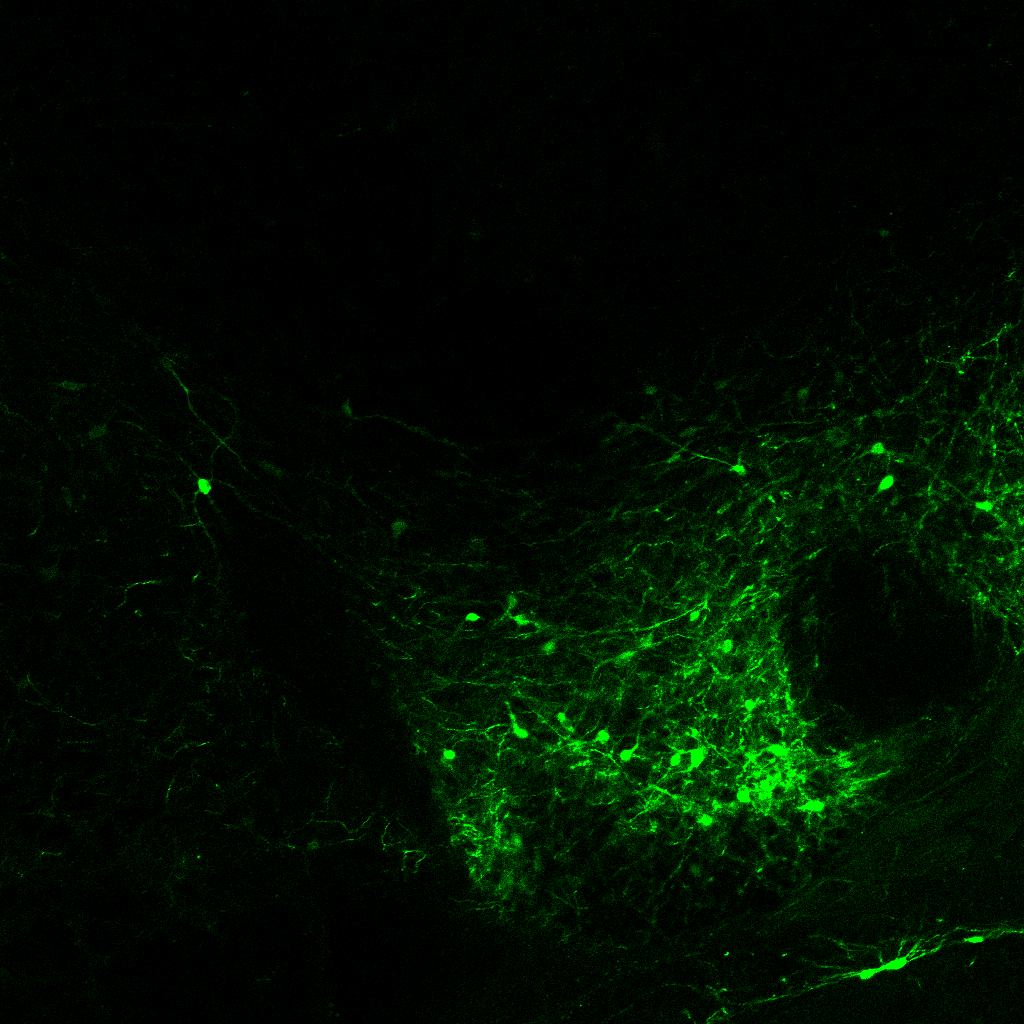

Supplement: Supplementary file 10 — Source data Fig. 7 [file 44318_2025_430_MOESM10_ESM.zip › Figure 7/Figure7L/GCaMP_RGB_FITC.tif]

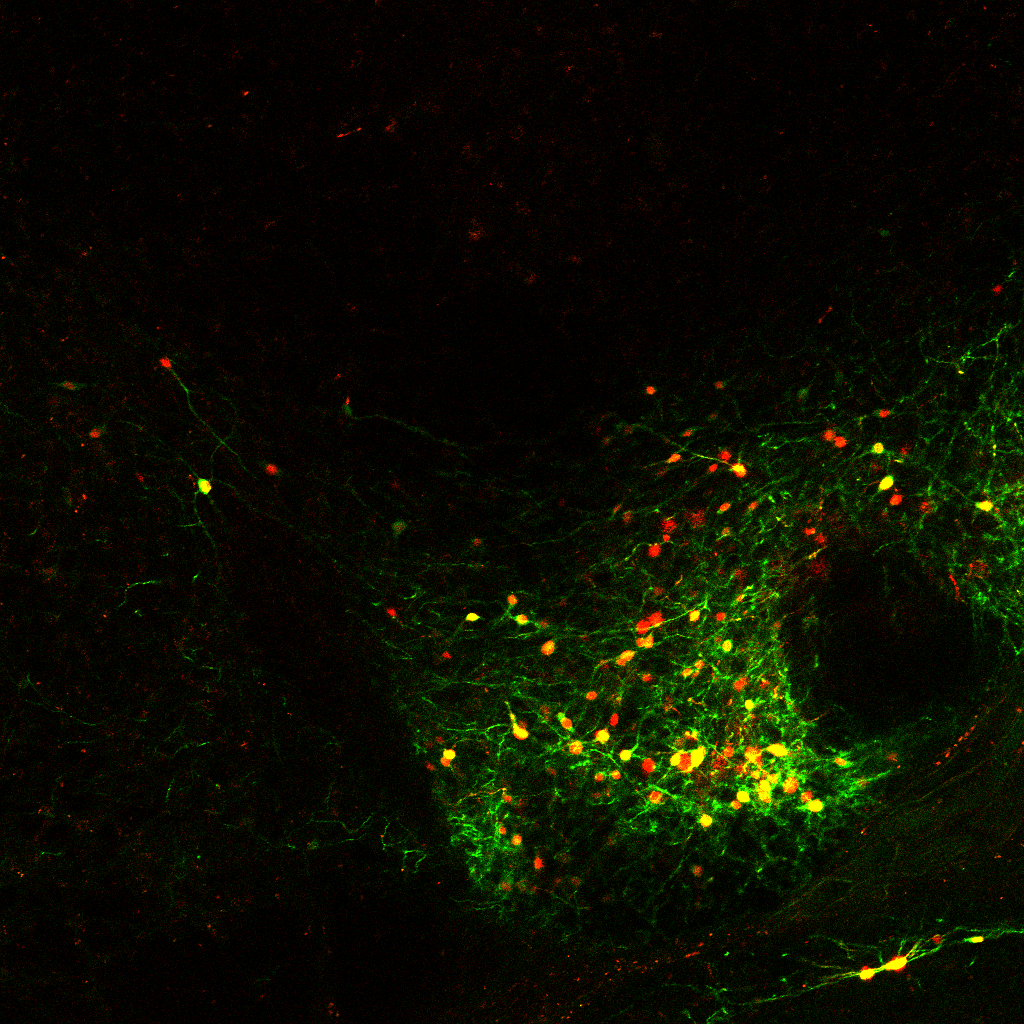

Supplement: Supplementary file 10 — Source data Fig. 7 [file 44318_2025_430_MOESM10_ESM.zip › Figure 7/Figure7L/Merge_RGB.tif]

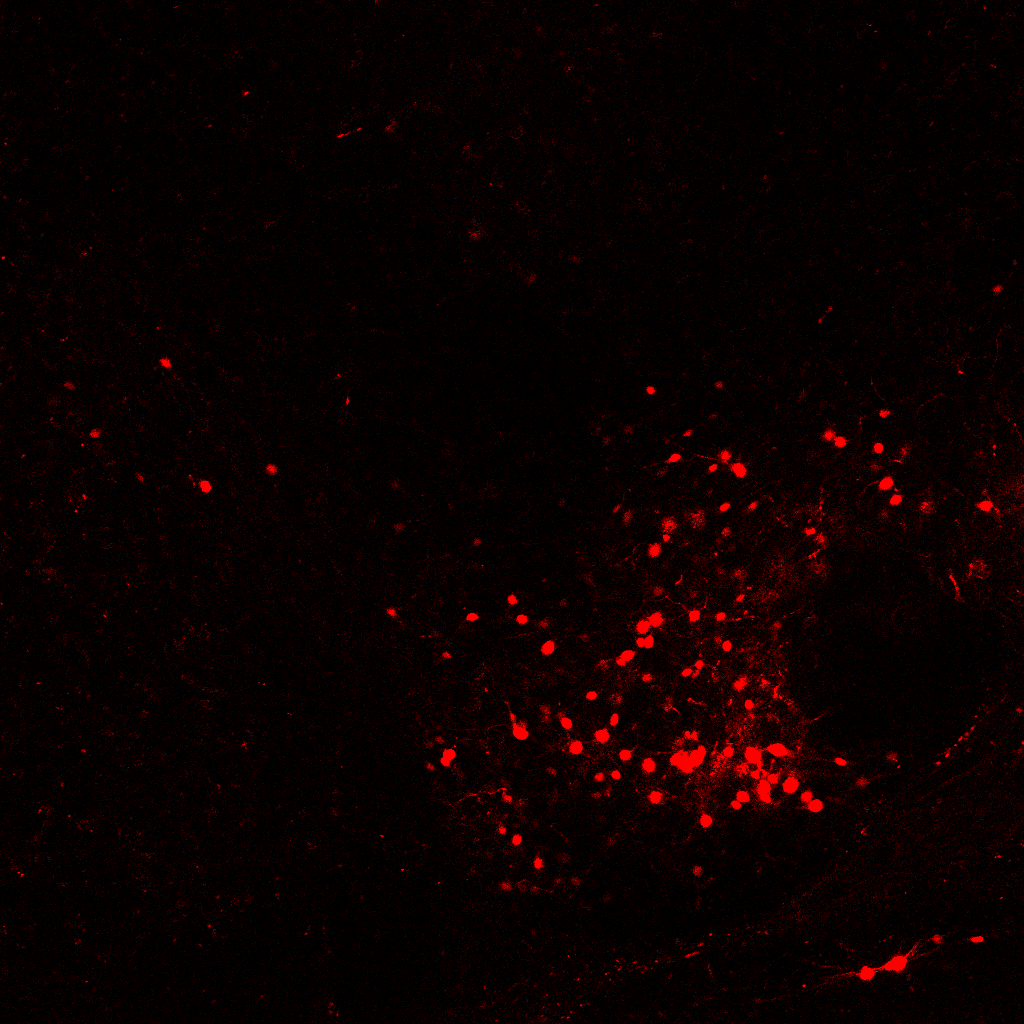

Supplement: Supplementary file 10 — Source data Fig. 7 [file 44318_2025_430_MOESM10_ESM.zip › Figure 7/Figure7L/Tdomato RGB_TRITC.tif]

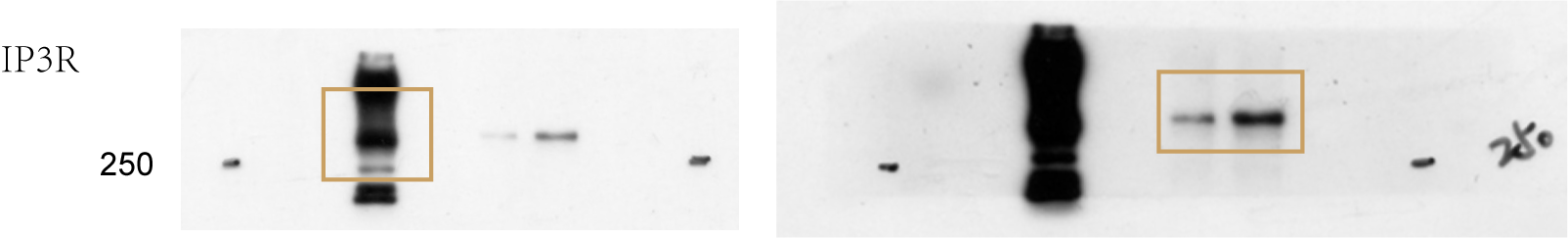

Supplement: Supplementary file 11 — Source data Fig. 8 [file 44318_2025_430_MOESM11_ESM.zip › Figure 8/Figure8E/IP3R1.tif]

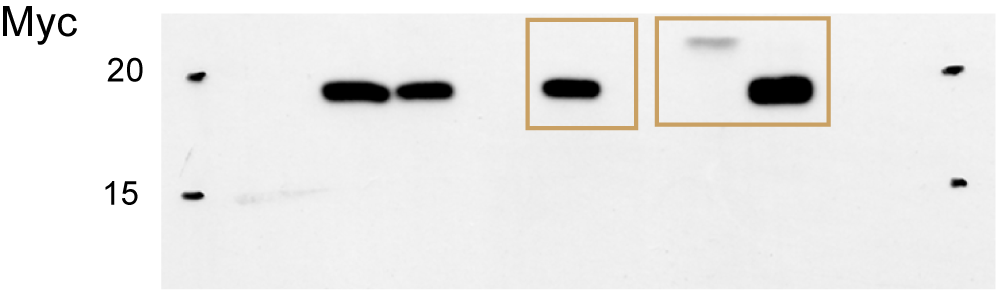

Supplement: Supplementary file 11 — Source data Fig. 8 [file 44318_2025_430_MOESM11_ESM.zip › Figure 8/Figure8E/Myc.tif]

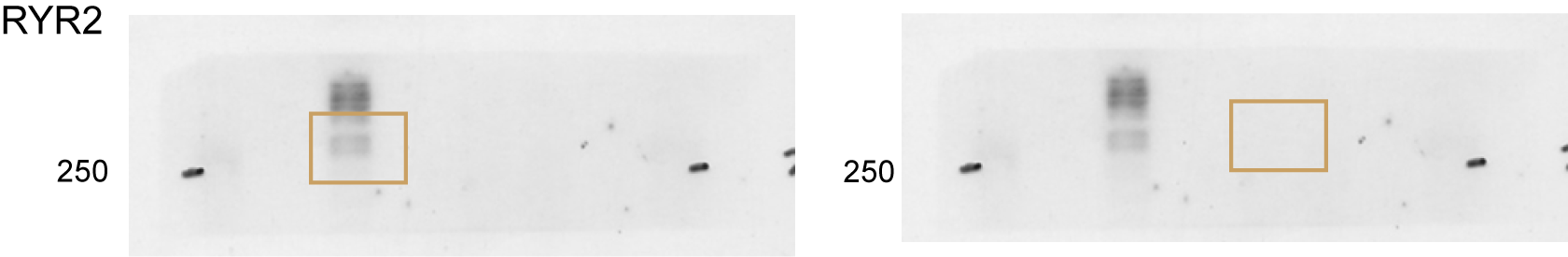

Supplement: Supplementary file 11 — Source data Fig. 8 [file 44318_2025_430_MOESM11_ESM.zip › Figure 8/Figure8E/RYR2.tif]

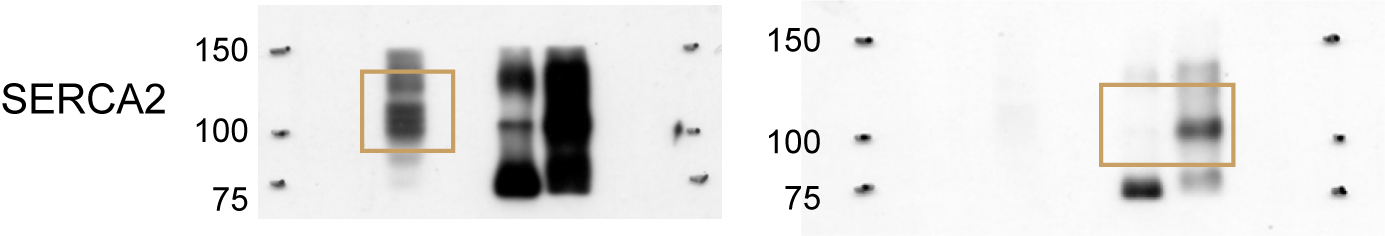

Supplement: Supplementary file 11 — Source data Fig. 8 [file 44318_2025_430_MOESM11_ESM.zip › Figure 8/Figure8E/SERCA2.tif]

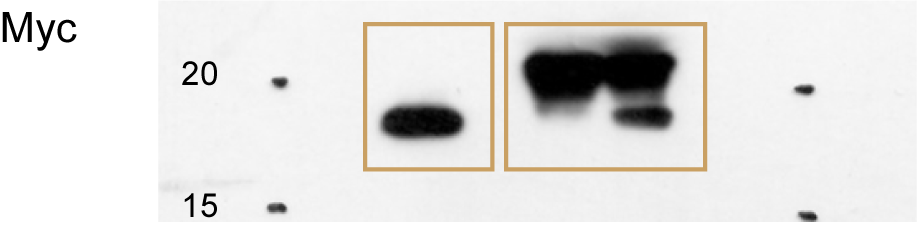

Supplement: Supplementary file 11 — Source data Fig. 8 [file 44318_2025_430_MOESM11_ESM.zip › Figure 8/Figure8G/Myc.tif]

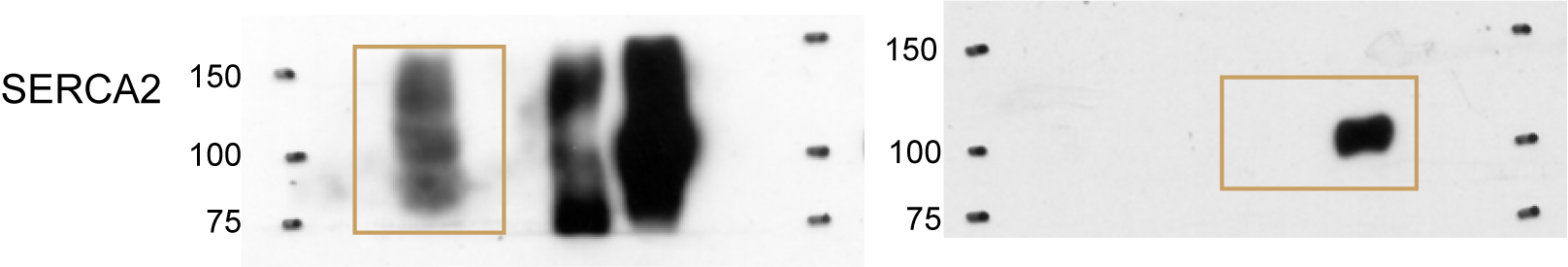

Supplement: Supplementary file 11 — Source data Fig. 8 [file 44318_2025_430_MOESM11_ESM.zip › Figure 8/Figure8G/SERCA2.tif]

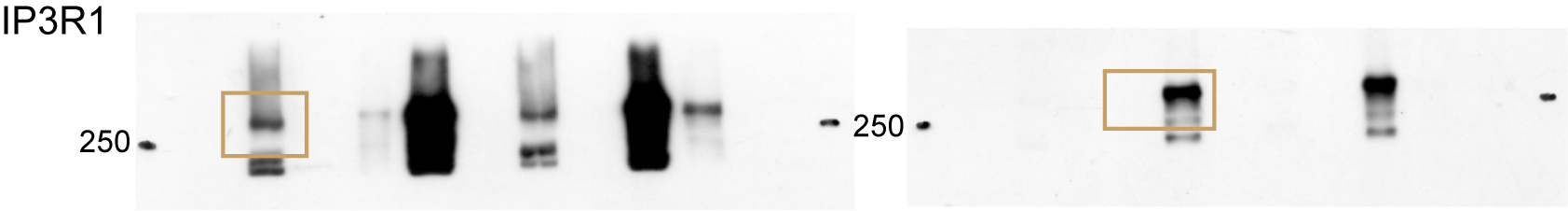

Supplement: Supplementary file 11 — Source data Fig. 8 [file 44318_2025_430_MOESM11_ESM.zip › Figure 8/Figure8I/IP3R1.tif]

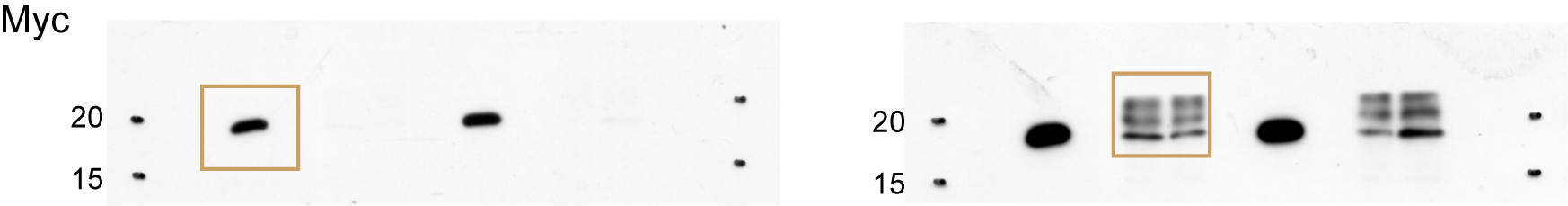

Supplement: Supplementary file 11 — Source data Fig. 8 [file 44318_2025_430_MOESM11_ESM.zip › Figure 8/Figure8I/Myc.tif]

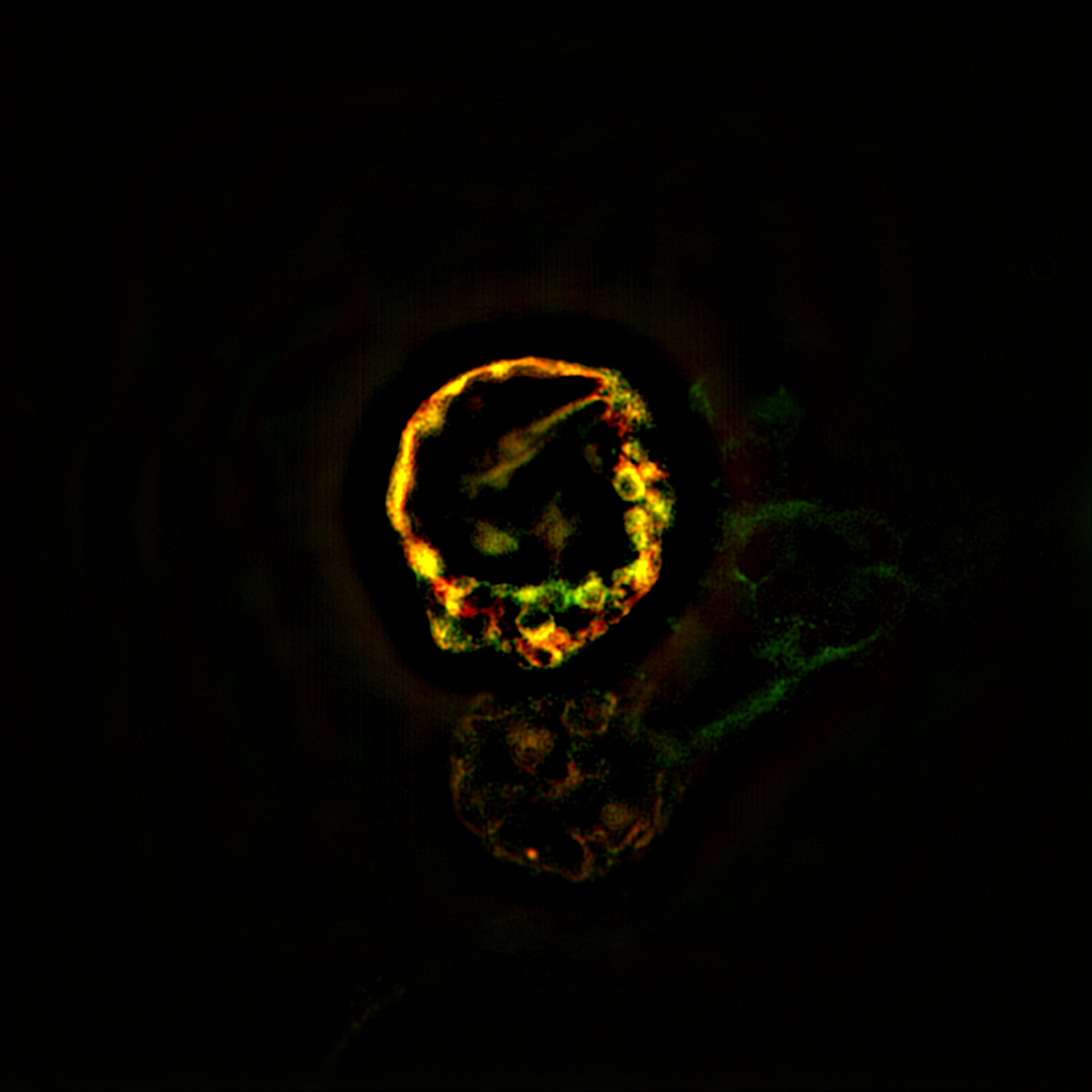

Supplement: Supplementary file 11 — Source data Fig. 8 [file 44318_2025_430_MOESM11_ESM.zip › Figure 8/Figure8K/selt u49c-4 reconstructed.tif]

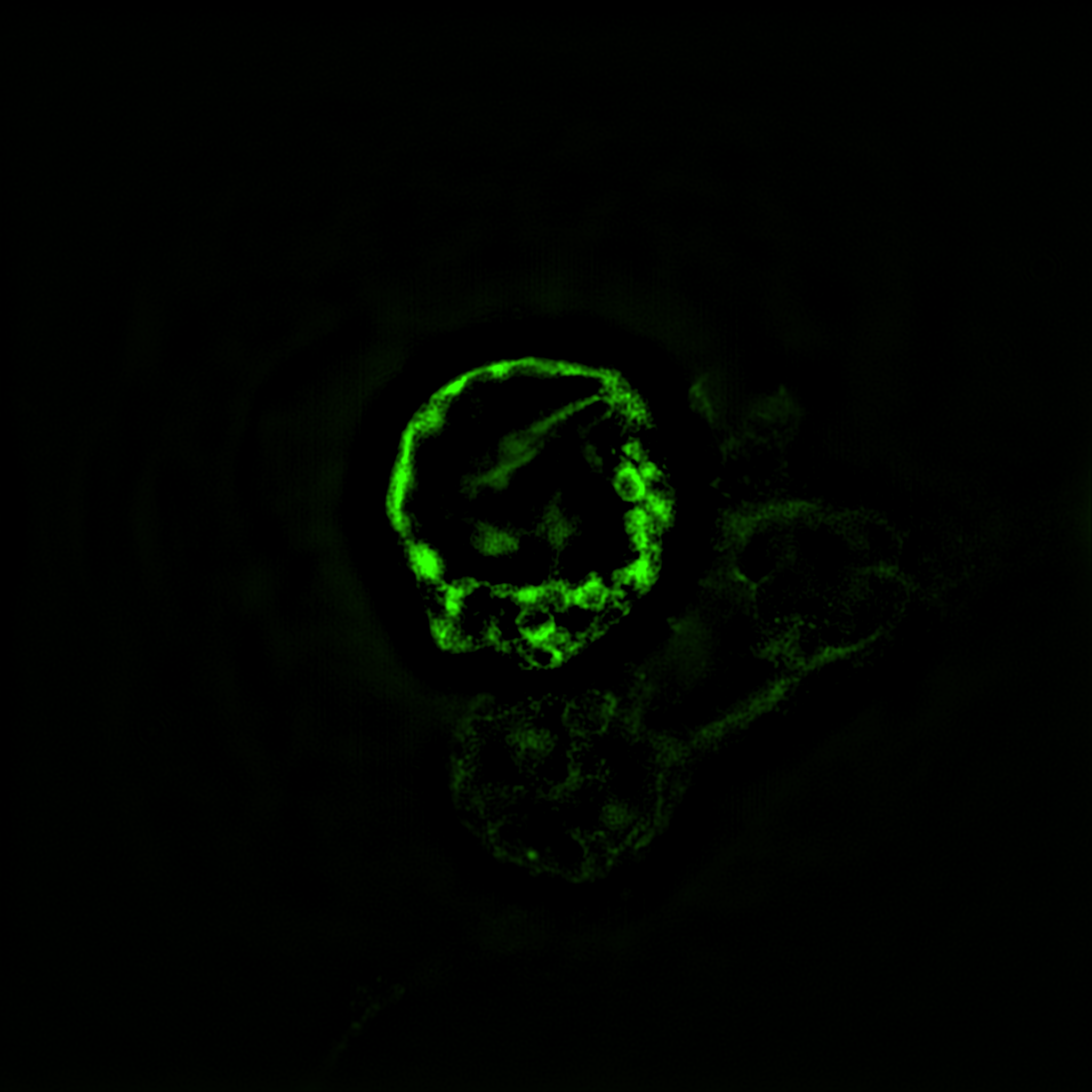

Supplement: Supplementary file 11 — Source data Fig. 8 [file 44318_2025_430_MOESM11_ESM.zip › Figure 8/Figure8K/selt u49c-4 reconstructedc1.tif]

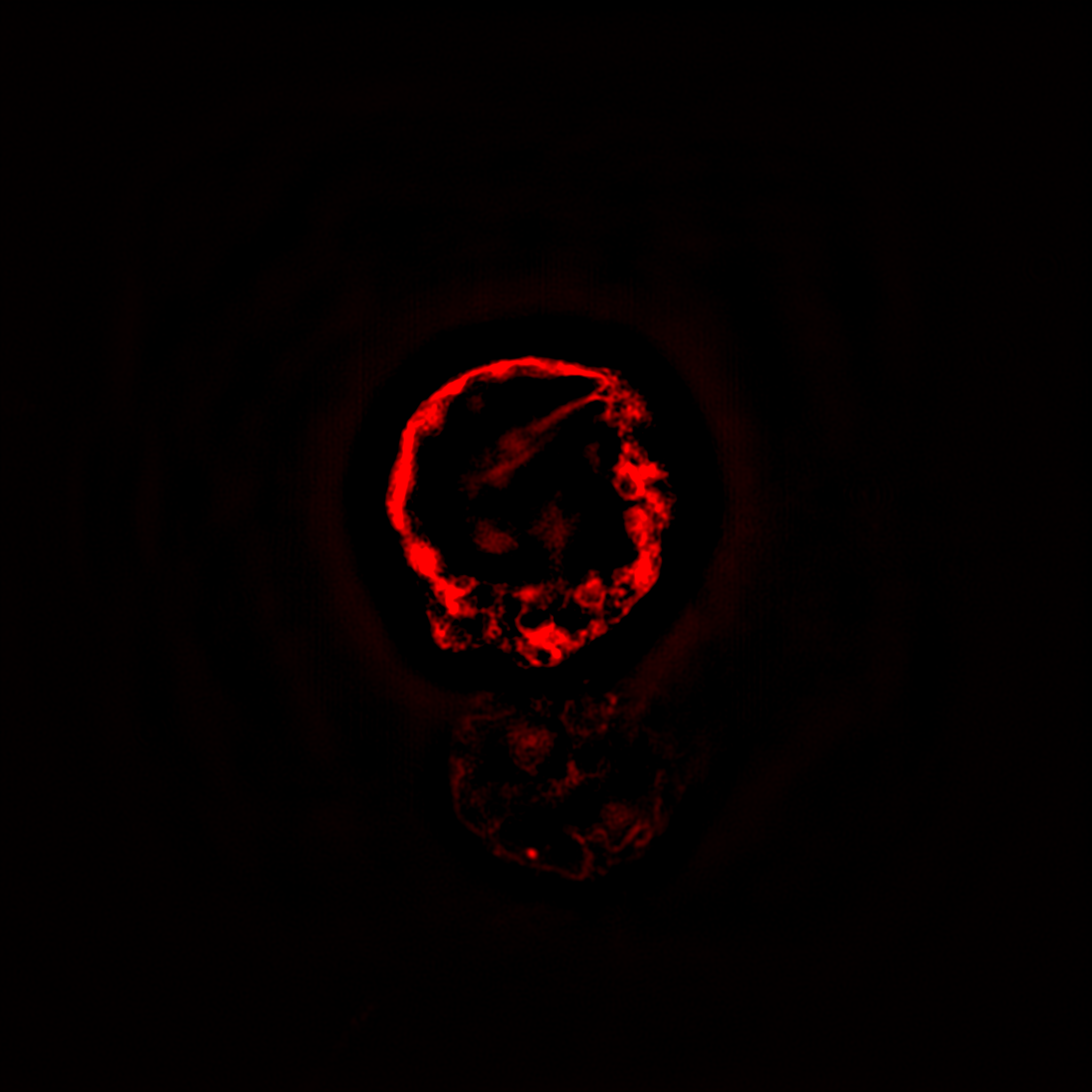

Supplement: Supplementary file 11 — Source data Fig. 8 [file 44318_2025_430_MOESM11_ESM.zip › Figure 8/Figure8K/selt u49c-4 reconstructedc2.tif]

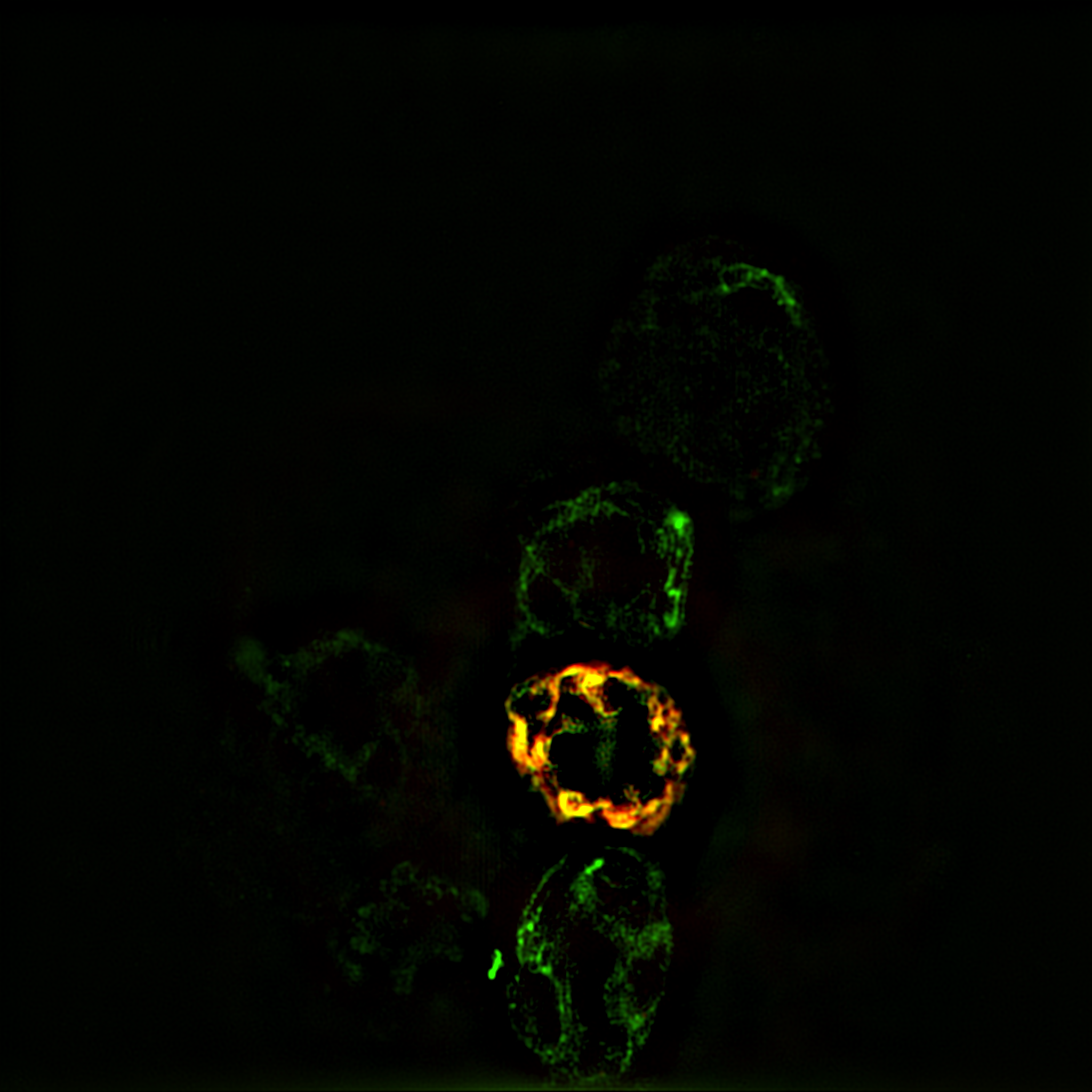

Supplement: Supplementary file 11 — Source data Fig. 8 [file 44318_2025_430_MOESM11_ESM.zip › Figure 8/Figure8M/selt wt constructed.tif]

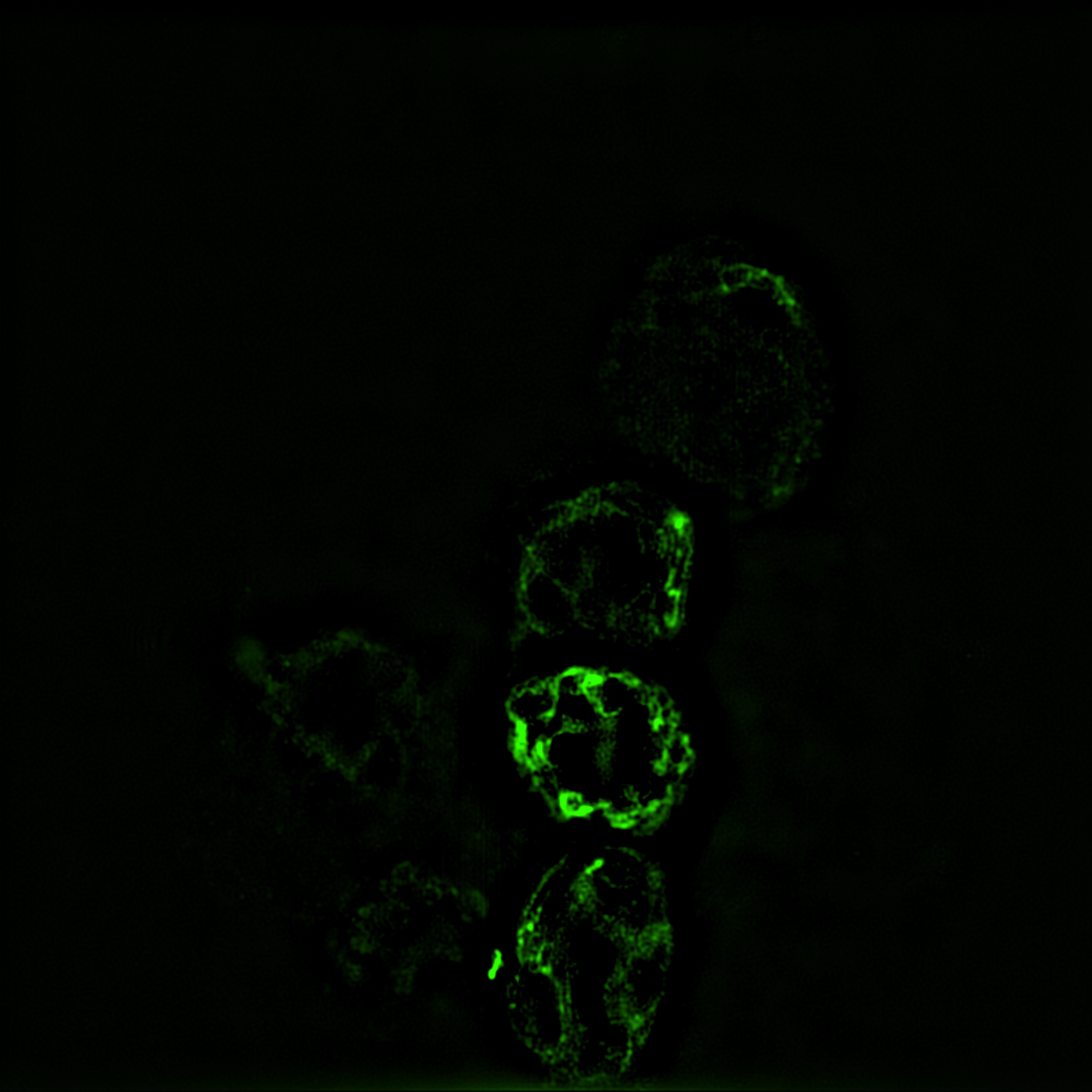

Supplement: Supplementary file 11 — Source data Fig. 8 [file 44318_2025_430_MOESM11_ESM.zip › Figure 8/Figure8M/selt wt constructedc1.tif]

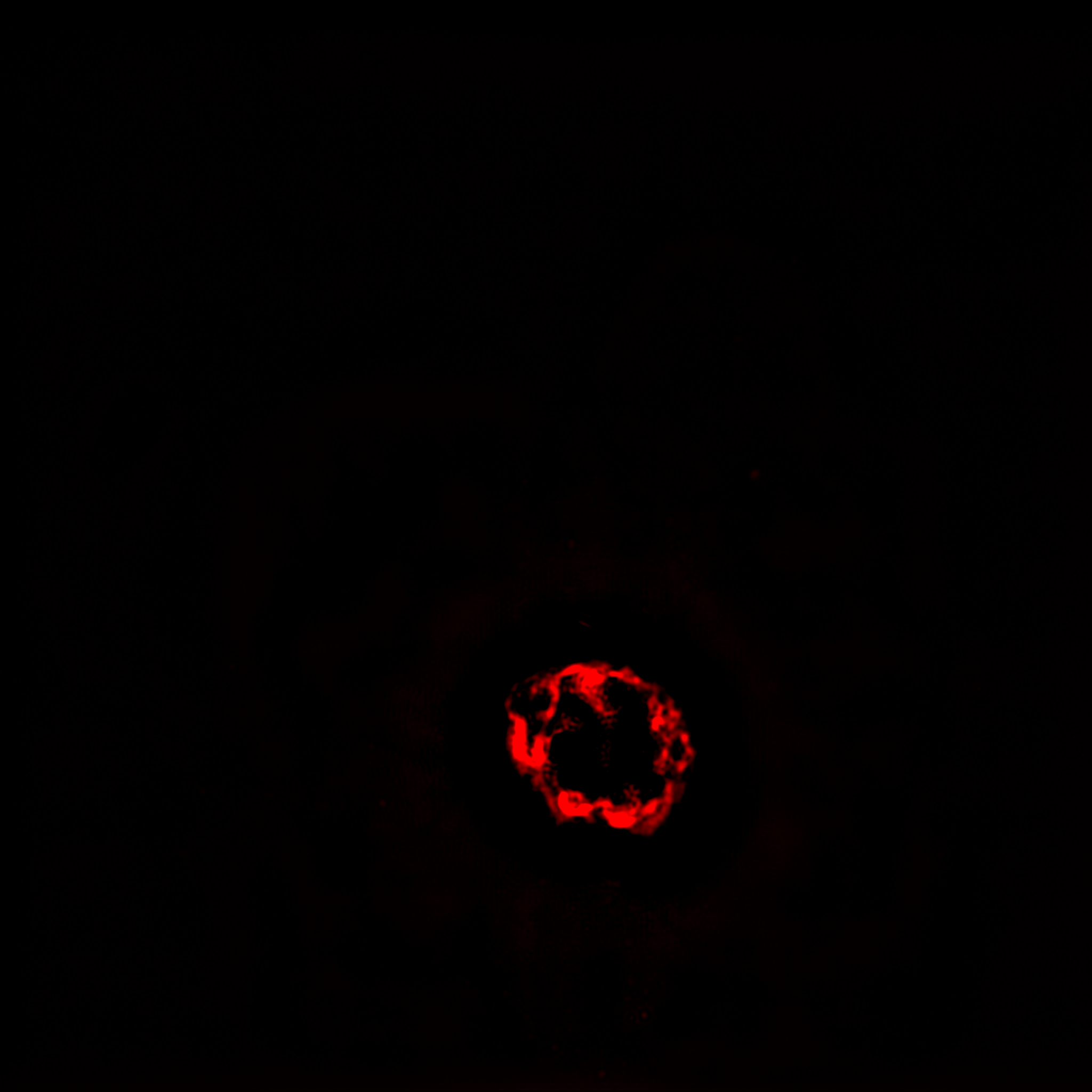

Supplement: Supplementary file 11 — Source data Fig. 8 [file 44318_2025_430_MOESM11_ESM.zip › Figure 8/Figure8M/selt wt constructedc2.tif]
